# Supplementary figures and images for: Diagnosed prevalence of Ehlers-Danlos syndrome and hypermobility spectrum disorder in Wales, UK: a national electronic cohort study and case–control comparison
Source: BMJ Open. 2019 Nov 4;9(11):e031365. doi: 10.1136/bmjopen-2019-031365 (PMC6858200; doi:10.1136/bmjopen-2019-031365)

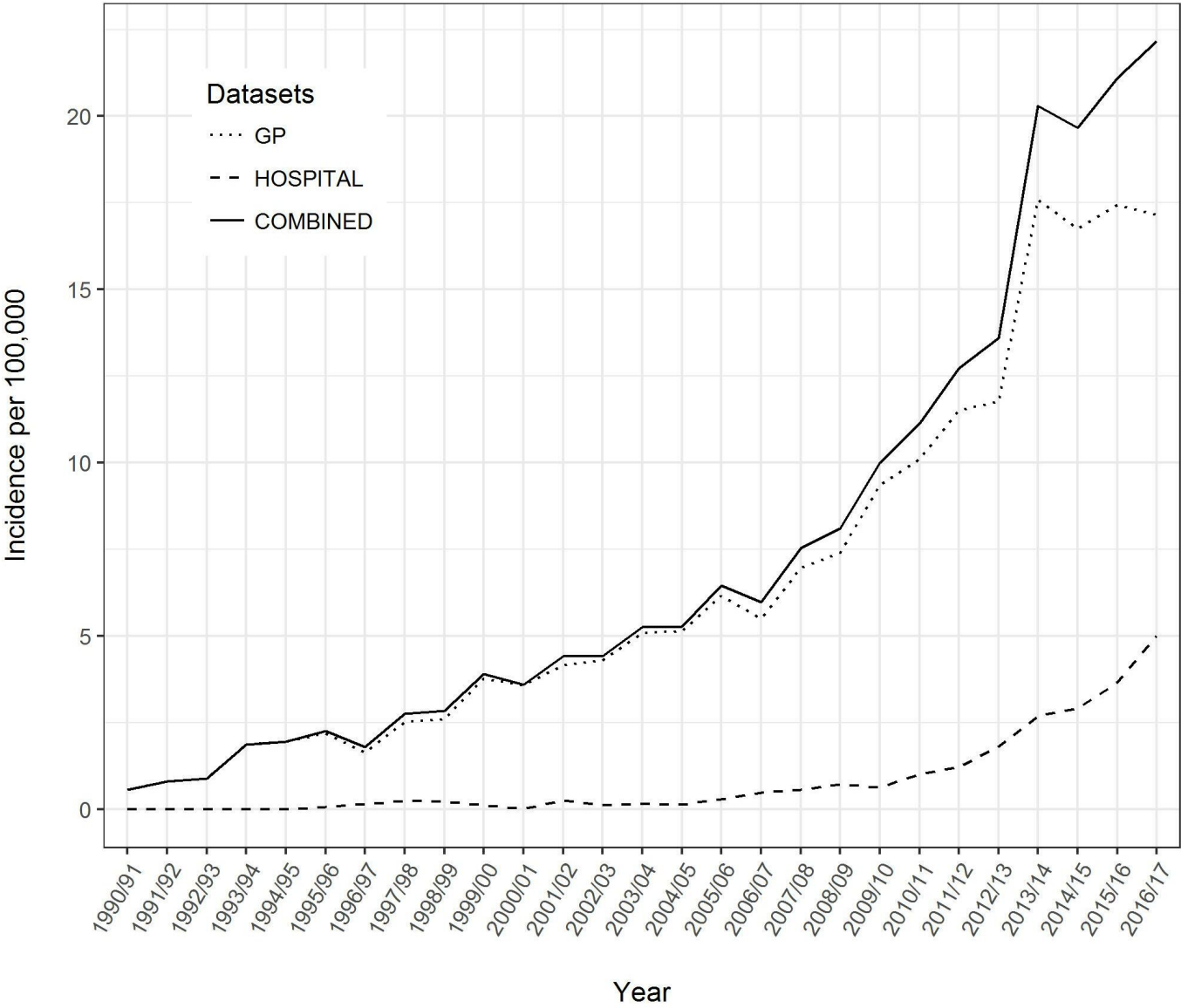

Supplement: Supplementary data [file bmjopen-2019-031365supp001.pdf]
