# Supplementary material for: Diagnosed prevalence of Ehlers-Danlos syndrome and hypermobility spectrum disorder in Wales, UK: a national electronic cohort study and case–control comparison
Source: BMJ Open. 2019 Nov 4;9(11):e031365. doi: 10.1136/bmjopen-2019-031365 (PMC6858200; doi:10.1136/bmjopen-2019-031365)
Supplement: Author's manuscript [file bmjopen-2019-031365.draft_revisions.pdf]

BMJ Open is committed to open peer review. As part of this commitment we make the peer review history of every article we publish publicly available.

When an article is published we post the peer reviewers' comments and the authors' responses online. We also post the versions of the paper that were used during peer review. These are the versions that the peer review comments apply to.

The versions of the paper that follow are the versions that were submitted during the peer review process. They are not the versions of record or the final published versions. They should not be cited or distributed as the published version of this manuscript.

BMJ Open is an open access journal and the full, final, typeset and author-corrected version of record of the manuscript is available on our site with no access controls, subscription charges or pay-per-view fees (http://bmjopen.bmj.com).

If you have any questions on BMJ Open's open peer review process please email info.bmjopen@bmj.com

BMJ Open

Diagnosed prevalence of Ehlers-Danlos syndrome and Hypermobility Spectrum Disorder: a national electronic cohort study and case-control comparison

Journal:	*BMJ Open*
Manuscript ID	bmjopen-2019-031365
Article Type:	Research
Date Submitted by the Author:	30-Apr-2019

Complete List of Authors:	Demmler, Joanne; Swansea University, Swansea University Medical School<br>Atkinson, Mark; Swansea University, Swansea University Medical School<br>Reinhold, Emma; Royal College of General Practitioners<br>Choy, Ernest; Cardiff University, School of Medicine<br>Lyons, Ronan; Swansea University, Swansea University Medical School<br>Brophy, Sinead; Swansea University, Swansea University Medical School

Keywords:	Heritable Disorders of Connective Tissue, Ehlers-Danlos Syndromes, Joint Hypermobility Syndrome, Hypermobility Spectrum Disorder, Health Data Linkage, Prevalence

SCHOLARONE™ Manuscripts

Diagnosed prevalence of Ehlers-Danlos syndrome and Hypermobility Spectrum Disorder: a national electronic cohort study and case-control comparison

Joanne C Demmler, Mark D Atkinson, Emma J Reinhold, Ernest Choy, Ronan A Lyons, Sinead T Brophy

Joanne C Demmler, Lecturer, Health Data Research UK, Swansea University, Singleton Park, Swansea SA2 8PP, UK. j.demmler@swansea.ac.uk.

Mark D Atkinson, Research Officer, Secure Anonymised Information Linkage (SAIL) Databank, Swansea University, Singleton Park, Swansea SA2 8PP, UK. m.atkinson@swansea.ac.uk.

Emma J Reinhold, RCGP Clinical Champion for the Ehlers-Danlos Syndromes, Clinical Innovation and Research Centre, Royal College of General Practitioners, 30 Euston Square, London, NW1 2FB. e.reinhold@doctors.org.uk.

Ernest Choy, Professor, Arthritis Research UK CREATE Centre, Section of Rheumatology, Division of Infection and Immunity, Cardiff University, Cardiff CF14 4YS, UK. ChoyEH@cardiff.ac.uk.

Ronan A Lyons, Professor, Health Data Research UK, Swansea University, Singleton Park, Swansea SA2 8PP, UK. r.a.lyons@swansea.ac.uk.

Sinead T Brophy, Professor, Health Data Research UK, Swansea University, Singleton Park, Swansea SA2 8PP, UK. s.brophy@swansea.ac.uk.

Correspondence to: JC Demmler j.demmler@swansea.ac.uk. Tel: 44 (0) 1792 295674

For peer review only

For peer review only - http://bmjopen.bmj.com/site/about/guidelines.xhtml

Abstract

Objectives: To describe the epidemiology of diagnosed Hypermobility Spectrum Disorder (HSD) and Ehlers-Danlos Syndromes (EDS) under the 2017 classification using linked electronic medical records. To examine whether these conditions remain rare and primarily affect the musculoskeletal system.

Design: Nationwide linked electronic cohort and nested case-control study

Setting: Routinely collected data from primary care and hospital admissions in Wales, UK.

Participants: People within the primary care or hospital data systems with a coded diagnosis of Ehlers-Danlos syndrome (EDS) or Joint Hypermobility Syndrome (JHS) between 01/07/1990 and 30/06/2017.

Main outcome measures: Combined prevalence of JHS and EDS in Wales. Additional diagnosis and prescription data in those diagnosed with EDS or JHS compared with matched controls.

Results: We found 6,021 individuals (male: 30%, female: 70%) with a diagnostic code of either EDS or JHS. This gives a diagnosed point prevalence of 194.2 per 100,000 in 2016/17 or roughly 10 cases in a practice of 5000 patients. There was a pronounced gender difference of 8.5 years (95% CI: 7.70 to 9.22) in the mean age at diagnosis. EDS or JHS was not only associated with high odds for other musculoskeletal diagnoses and drug prescriptions, but also with significantly higher odds of a diagnosis in other disease categories (e.g. mental health, nervous and digestive systems) and higher odds of a prescription in most disease categories (e.g. gastro-intestinal and cardiovascular drugs) within the 12 months before and after the first recorded diagnosis.

Conclusions: EDS and JHS (since March 2017 classified as EDS or HSD) have historically been considered rare diseases only affecting the musculoskeletal system and soft tissues. These data demonstrate that both of these assertions should be reconsidered.

For peer review only - http://bmjopen.bmj.com/site/about/guidelines.xhtml

Key Words: Heritable Disorders of Connective Tissue, Ehlers-Danlos Syndromes, Joint Hypermobility Syndrome, Hypermobility Spectrum Disorder, Health Data Linkage, Prevalence

Word Count: 3687

Article Summary

Article focus

- To provide physicians with an overview of the epidemiology of Ehlers-Danlos Syndromes; prevalence and co-morbidities.
- EDS and other hereditary connective tissue disorders have been considered rare.
- Early diagnosis is important, but there is often a long diagnostic delay.
- Connective tissue disorders are recognised as affecting more than the musculoskeletal system.

Key messages

- JHS/EDS are now diagnosed at a rate of 1 in 500 meaning these conditions are not rare.
- These figures will significantly underestimate the true prevalence as they only reflect diagnosed cases.
- The conditions are associated with a high probability of additional diagnoses affecting most body systems (e.g. mental health, nervous and digestive systems) in both adults and children.
- These findings have significant implications for policy makers and service planning.

Strengths and Limitations

For peer review only - http://bmjopen.bmj.com/site/about/guidelines.xhtml

- Large cohort and nested case-control studies based on whole population routinely collected health data from primary and secondary care
- We are unable to quantify how many people are suffering from hypermobile EDS (hEDS) or HSD but remain undiagnosed, nor can we make any statement on the reliability of the diagnoses
- Although we only compared codes at Read chapter level all diagnoses and prescriptions can be matched to conditions found in the EDS/JHS literature

Additional Resources

RCGP Clinical Toolkit on the Ehlers-Danlos Syndromes www.rcgp.org.uk/eds

RCGP Podcast “Introduction to Ehlers-Danlos Syndromes”

https://audioboom.com/posts/6896541-introduction-to-ehlers-danlos-syndromes

For peer review only

For peer review only - http://bmjopen.bmj.com/site/about/guidelines.xhtml

Introduction

The Ehlers-Danlos Syndromes (EDS) are a group of hereditary connective tissue disorders which mainly affect collagen. The nomenclature of these conditions has undergone a number of iterations which makes discussion of their prevalence complicated (see Textbox 1).

For many decades, studies have quoted a prevalence rate of 1 in 5000 for EDS, although the origin of this figure is unclear, seeming to appear first in a medical textbook [1 2] as an unreferenced "reasonable estimate". Thus, these syndromes have long been categorised as rare diseases, defined in the European Union as those affecting fewer than 50 in 100,000 people [3]. Kulas Søborg et al. [4] recently reported a prevalence of 20 per 100,000 for EDS in a nationwide Danish cohort based on secondary health care data up to 2012, but importantly, this data did not include patients who had received the considerably more common JHS diagnosis, now included in the latest revised classification. It is possible to extrapolate a combined population prevalence figure for JHS and EDS for Sweden [5] of around 120 per 100,000 from a study focussing on comorbid mental health issues, but no investigators have thus far set out to investigate the combined diagnosed prevalence of JHS/EDS within a population.

Although common features of these conditions are arthralgia, soft tissue injury and joint instability [6], over the last two decades it has become clear that their clinical features are not limited to musculoskeletal and cutaneous involvement, but are multisystemic [7-9]. In the special edition of the American Journal of Medical Genetics dedicated to EDS in March 2017, papers covered links to cardiovascular autonomic [10] and gastrointestinal dysfunction [11] as well as psychiatric and neurodevelopmental disorders [5 12]. Chronic disabling fatigue [13] and pain syndromes [14] were also recognised as common and multifactorial issues. Gynaecological [15 16] and obstetric [17] issues are also reported in this population. There is also an emerging link with the potentially life-threatening condition of Mast Cell Activation

For peer review only - http://bmjopen.bmj.com/site/about/guidelines.xhtml

Syndrome [18 19]. There is some emerging evidence hinting that nutritional deficiencies [20 21] may play a key role, both seeming to be more prevalent in these patients and possibly implicated in the development of some of the complications.

Early diagnosis is found to be crucial to patients [22] to enable the provision of appropriate treatment, as well as to prevent later onset complications [7]. Establishing the diagnosis of EDS/HSD is often problematic for patients, which interferes with the early detection, treatment and prevention of further escalations of recognised symptoms, disability and more elaborate complications. A mean of 14 years elapses between the first clinical manifestations and the actual diagnosis [23]. For 25% of patients this delay lasts over 28 years [23]. “A misdiagnosis was given to 56% of patients [resulting in] inappropriate treatment in 70% of the patients… For 86% of the patients, the delay in diagnosis was considered responsible for deleterious consequences.” [23(p.137)]

It is possible that some of these difficulties arise from the widespread belief amongst clinicians that EDS is rare. It is therefore of clinical importance to establish better estimates of current prevalence. Conventional studies tend to be based in restricted clinical settings, such as rheumatology clinics, and are therefore limited by the number of recruited patients and biased by severity/type of patients referred. It has been shown that using linked health data is an economic and effective alternative to performing *de novo* longitudinal studies, including rare conditions [24 25]. We used routinely held data from primary and secondary care sources to examine the epidemiology of people with a diagnostic code for EDS/JHS in Wales. We then conducted a nested case- control study to study the number of diagnoses across all body/disease systems and prescription usage in order to test the widespread belief that these conditions are primarily musculoskeletal in nature, rather than multi-system disorders.

Methods

For peer review only - http://bmjopen.bmj.com/site/about/guidelines.xhtml

Study design: Nationwide electronic cohort study

Anonymised record linkage and hosting is carried out in the Secure Anonymised Information Linkage (SAIL) databank [26] on routinely collected data held in health and social care datasets. All data within the SAIL gateway are treated in accordance with the Data Protection Act 2018 and complies with GDPR.

We used data from a variety of datasets between 01/07/1990 and 30/06/2017 to create the anonymised e-cohort and case control studies. The Primary Care data covers about 80% of all coded information held by General Practitioners (GPs) in Wales. The Welsh Demographics Service (WDS) contains key statistics, such as gender, week of birth, date of death and practice migration status for everyone in Wales registered with a GP. The Patient Episode Database for Wales (PEDW) contains all inpatient hospital admissions to a Welsh hospital. WDS and PEDW data are available for the whole of Wales. The SAIL databank enables the anonymised matching of individuals across these different datasets using a person level anonymised linkage field (ALF) [26].

Cohort preparation

We identified Welsh residents with a Read Version 2 [27] diagnostic code of EDS or JHS in primary care data or ICD-10 diagnostic codes [28] in secondary care data (hospital admissions) between 01/07/1990 (or the start of the dataset if later) and 30/06/2017. This date marks the end of maximum data coverage across all datasets. The EDS sub-classification in Read Version 2 contains some, but not all, of the subtypes which were in use prior to 1997 and as a result, the reliability of any subtype data must be highly questionable (see Table 1). ICD-10 codes do not distinguish between any subtypes of EDS (see Table 1). Only ALF's with good matching status were included in the study, i.e. direct match on either NHS number or on surname, first name, postcode, date of birth and gender; or fuzzy matching with a probability of >= 90%.

For peer review only - http://bmjopen.bmj.com/site/about/guidelines.xhtml

We created one dataset for diagnoses in the GP data and another for diagnoses in the hospital data. Both datasets were linked to the week of birth, gender and date of death information in WDS on their ALF and then combined to create a cohort of people with EDS/JHS in either GP or hospital data, identifying any duplications and keeping the earliest diagnosis date for any individual appearing in both datasets.

Analysis

Data linkage and data preparation within the SAIL databank were conducted using IBM DB2 10.5 SQL. Data were then imported into R (Version 3.4.1) [29], which was used for all statistical analyses. The mean age at first diagnosis between male and female subjects was compared and confidence intervals of the difference calculated.

The denominator of the diagnosed prevalence and incidence of EDS and JHS in secondary care was calculated based on the total number of individuals with recorded gender, registered and living in Wales between 01/07/1990 and 30/06/2017 for each full year of the study respectively. The prevalence and incidence in primary care denominator was further adjusted to include only people living in Wales and whose GP practice was contributing data to SAIL. The prevalence and incidence in primary and secondary care was then added together to create an overall estimate of the prevalence and incidence in Wales.

Case-Control Comparison

A nested case control method was used. Each case was matched to 4 controls with the same gender and similar age profiles (within 45 days of the week of birth). We implemented strict criteria for selection to the case-control cohort. Both cases and controls had to (a) have uninterrupted GP registrations for 1 year before and 1 year after the date of the relevant diagnosis (or died during follow-up); (b) be registered with a GP submitting data to SAIL either at the matching date or afterwards; (c) have been registered with a GP that consistently recorded data across their patient profile. The latter avoids diagnoses that were retrospectively entered for a time period when the GP practice did not fully implement the

For peer review only - http://bmjopen.bmj.com/site/about/guidelines.xhtml

use of electronic records (less than 10% of the data they recorded during 2009). Although this reduced the number of cases and controls we were able to analyse, it avoids data quality bias, especially during the early years of this study, when GPs were converting to the use of computer systems and databases. Controls with any type of diagnosed hereditary connective tissue disorder were excluded. Preliminary analysis of the combined cohort indicated that adjustment for deprivation was not necessary (i.e. equal distribution of people across deprivation quintiles). We then calculated odds ratios between cases and controls using Read chapters. All results that affected at least 5 cases or 20 controls were visualised using forest plots.

Ethical approval

The study design uses anonymised data and therefore the need for ethical approval and participant consent was waived by the approving Institutional Review Board, the UK National Health Service Research Ethics Committee. The SAIL independent Information Governance Review Panel (IGRP) approved the study.

Patient and Public Involvement

Two of the authors of this paper have been diagnosed with symptomatic joint hypermobility disorders. This study used routinely collected data, we were not able to involve members of the public but will be disseminating our findings widely, including directly to patients via social media and through our links with patient organisations.

Results

EDS/JHS in Primary Care data

5,355 individuals with a diagnosis of either EDS or JHS with valid birth and gender information were identified. Of these, 4,654 (87%) had a diagnosis of JHS and 701 (13%) of EDS. The Read code for the EDS subtype was only used for 136 (19%) individuals with 114

For peer review only - http://bmjopen.bmj.com/site/about/guidelines.xhtml

For peer review only

(16%) identified as EDS-Hypermobility Type and 22 (3%) as other EDS subtypes. 3,759 (70%) of those diagnosed with EDS/JHS were female (see Figure 1).

EDS/JHS in Hospital Data

A total of 1,298 individuals were found in the hospital data of whom 970 (75%) were female: 745 (57%) had a diagnosis of JHS and 553 (43%) EDS (see Figure 1).

Demographics of combined EDS/JHS cohort

5,355 (89%) of the cases could be found in the primary care data with the remainder in the hospital cohort. Combining the results from primary and secondary care led to a cohort of 6,021 distinct individuals. 5,064 (84%) were coded with JHS and 957 (16%) with EDS. 4,244 (70%) of patients were female. The age at first diagnosis peaked in the age group 5-9 years for males and 15-19 years for females (see Figure 2). There was a significant difference of 8.5 years in the mean age of diagnosis between males and females (95% CI: 7.70 to 9.22): 9.6 years in EDS (95% CI: 6.85 to 12.31) and 8.3 years in JHS (95% CI: 7.58 to 9.11). 72% of males were diagnosed during childhood (age < 18 years) in contrast to only 41% of females.

2016/17 is the latest year for which we have complete data and could therefore derive prevalence. During this year, 2,668,902 people were registered with a GP in Wales submitting data to SAIL, of whom 4,598 had a diagnostic code of EDS/JHS which first appeared in the primary care data (172 in 100,000). A further 711 people out of the 3,239,153 registered with any GP in Wales during 2016/17 had an EDS/JHS diagnosis which first appears in secondary care data (22 in 100,000). There is an increasing rate of coded diagnoses throughout the period. Assuming that the GP data is representative of the whole of Wales this leads to a combined point prevalence of 194 in 100,000 at the end of the study period. This corresponds to about 10 cases in a practice of 5000 patients (see Figure 3). The incidence of EDS/JHS over this time period is shown in Supplement Figure 1.

Factors associated with JHS/EDS

For peer review only - http://bmjopen.bmj.com/site/about/guidelines.xhtml

2,597 cases had good GP data coverage at the age of diagnosis and could be matched by age and gender with controls (see Figure 1). 1,340 cases (male: 561; female: 779) were first diagnosed before the age of 18 years and 1,254 cases (male: 229; female: 1,025) above this age. The people in the nested case-control cohort were slightly older than the overall cohort (data not shown here).

Looking at the time frame of 1 year either side of the first coded diagnosis of EDS/JHS amongst young people (age < 18 years) there were significantly more additional diagnoses in 16 out of 20 Read code disease categories compared with their controls (see Figure 4a). The top three Read diagnosis chapters with increased odds for the EDS/JHS cohort were for musculoskeletal conditions (OR 9.36, 95% CI: 7.98 to 11.00), congenital anomalies (OR 5.89; 95% CI: 3.98 to 8.80) and mental disorders (OR 4.16; 95% CI: 3.29 to 5.27).

People that were diagnosed as adults (age >= 18 years) had also significantly more diagnoses in 16 out of 20 Read code categories than their controls. The top three Read diagnosis chapters for adults with higher odds in the EDS/JHS cohort were musculoskeletal disorders (OR 7.95; 95% CI: 6.95 to 9.12), congenital anomalies (OR 5.18; 95% CI: 2.78 to 9.78) and symptoms, signs and ill-defined conditions (OR 2.9; 95% CI: 2.5 to 3.37). Circulatory system disease (OR 2.29; 95% CI: 1.83 to 2.86) and mental disorders remained significant (OR 1.87; 95% CI: 1.57 to 2.22), but not to the same extend as they were for young people.

Young people showed significantly higher odds for prescriptions in 14 out of 17 Read code categories then their controls (see Figure 5a). The top three prescriptions Read chapters with increased odds for the EDS/JHS cohort were for (i) musculoskeletal drugs (OR 3.65; 95% CI: 3.18 to 4.18), (ii) gastro-intestinal drugs (OR 3.02; 95% CI: 2.54 to 3.58) and (iii) haematology/dietetic drugs (OR 2.54; 95% CI: 2.06 to 3.11).

Adults had significantly higher odds of prescriptions for 15 out of 17 Read code categories (see Figure 5b). The top three prescriptions with higher odds for EDS/JHS people were for

For peer review only - http://bmjopen.bmj.com/site/about/guidelines.xhtml

(i) musculoskeletal drugs (OR 5.17; 95% CI: 4.53 to 5.9), (ii) central nervous system drugs (OR 3.9; 95% CI: 3.41 to 4.46) and (iii) chemotherapy/immunosuppressant drugs (OR 3.03; 95% CI: 1.89 to 4.8). Gastro-intestinal drugs (OR 2.85; 95% CI: 2.5 to 3.24) and haematology/dietetic drugs (OR 2.21; 95% CI: 1.9 to 2.57) remain significant, but at slightly lower levels than in the young EDS/JHS population.

Discussion

This work examined the epidemiology of EDS and JHS and found a combined diagnosed prevalence of 194.2 per 100,000 (0.19%), or 1 in 500 people in Wales; hEDS or HSD within the 2017 classification. We found a steadily increasing rate of diagnosis over the past 27 years (see Supplement Figure 1), as well as higher rates of diagnoses for other conditions and prescriptions within 12 months (pre and post) of the recorded first diagnosis in most categories. This suggests that hEDS/HSD, when considered together, do not meet the definition of rare conditions [23], and have widespread effects across multiple body systems. It is well-known that EDS is poorly recognised in children [30 31]. Furthermore, children with hEDS often present with symptoms that can lead to a misdiagnosis of mental illness or consideration of child abuse [12 32]. Suspicion of abuse has been shown to be extremely damaging to the mental health of the parent(s) and can lead to an avoidance of accessing health care or other public services, such as schools [33]. The prolonged and sometimes traumatic diagnosis and/or misdiagnosis process in EDS can lead to further disengagement with services [34]. The lack of a timely diagnosis has great implications for disease management and progression and impedes the appropriate consideration of surgical interventions [7 35-38] as well as pregnancy and birth planning [17].

Strengths and Limitations

The strength of this study is that we were able to combine diagnostic codes from several primary and secondary health care providers to create a large cohort of individuals with EDS/JHS. We have 27 years of data with at least 11 years of very good data coverage in the

For peer review only - http://bmjopen.bmj.com/site/about/guidelines.xhtml

key datasets, which further improves with each data update of the SAIL databank, however data coverage for the first couple of years is less comprehensive.

The majority of subjects were identified via their primary care data, which is a strength and a weakness. As 89% of cases were identified through primary care data studies not using primary care data may underestimate the prevalence of hEDS/HSD. We are unable to quantify how many people are suffering from hEDS or HSD but remain undiagnosed. However, we cannot comment on the reliability of the diagnoses in the primary care dataset. It is also likely that the majority of cases were not actually diagnosed in primary care, but their entries were created through secondary care contacts, such as outpatient appointments or musculoskeletal assessment clinics, but coded data are lacking from these sources.

Although a snapshot of Read chapters codes that are more prevalent in our JHS/EDS cohort does not allow us to look at specific diagnoses and prescriptions, they can all be matched to conditions associated with EDS/JHS in the literature, for instance pain, fatigue, cardiovascular, gastrointestinal and gynaecological disorders, dysautonomia, mast cell activation as well as urinary tract infections [7].

We conclude that EDS/HSD are not rare conditions and are associated with significantly increased odds of additional diagnoses and use of medications across many body systems. There is a large gender difference in the age of diagnosis, with many women not diagnosed until adulthood. Early diagnosis, however, is crucial to patients, the administration of preventive therapies, the investigation of comorbid conditions and the overall management process. Further research is needed to understand patient pathways, comorbidities and progression of associated symptoms and diseases. Health services should be aware of these findings for the provision of training, diagnostic and treatment services for the many tens of thousands of patients living with these life-changing conditions throughout the United Kingdom and beyond.

Author contributions

For peer review only - http://bmjopen.bmj.com/site/about/guidelines.xhtml

JD conceived the project. SB and MA contributed to the study design and analysis plan. RAL validated clinical codes in primary care. EC validated clinical codes in secondary care. JD undertook the analysis. JD and ER carried out the literature reviews and drafted the manuscript. All authors reviewed the manuscript and approved the final version for submission.

Funding

This work was supported by The Farr Institute. The Farr Institute was supported by a 10-funder consortium: Arthritis Research UK, the British Heart Foundation, Cancer Research UK, the Economic and Social Research Council, the Engineering and Physical Sciences Research Council, the Medical Research Council, the National Institute of Health Research, the National Institute for Social Care and Health Research (Welsh Assembly Government), the Chief Scientist Office (Scottish Government Health Directorates), the Wellcome Trust, (MRC Grant No: MR/K006525/1).

This work was also supported by Health Data Research UK which receives its funding from HDR UK Ltd (NIWA1) funded by the UK Medical Research Council, Engineering and Physical Sciences Research Council, Economic and Social Research Council, Department of Health and Social Care (England), Chief Scientist Office of the Scottish Government Health and Social Care Directorates, Health and Social Care Research and Development Division (Welsh Government), Public Health Agency (Northern Ireland), British Heart Foundation (BHF) and the Wellcome Trust.

The above funders played no role in the study design, in the collection, analysis, or interpretation of data; in the writing of the report; or in the decision to submit the article for publication. The researchers are independent from the funders and all authors, external and internal, had full access to all of the data in the study and take responsibility for the integrity of the data and the accuracy of the data analysis.

For peer review only - http://bmjopen.bmj.com/site/about/guidelines.xhtml

Competing interests: All authors have completed the ICMJE uniform disclosure form at www.icmje.org/coi_disclosure.pdf and declare: no support from any organisation for the submitted work; no financial relationships with any organisations that might have an interest in the submitted work in the previous three years; no other relationships or activities that could appear to have influenced the submitted work.

Exclusive licence: The Corresponding Author has the right to grant on behalf of all authors and does grant on behalf of all authors, a worldwide licence (http://www.bmj.com/sites/default/files/BMJ%20Author%20Licence%20March%202013.doc) to the Publishers and its licensees in perpetuity, in all forms, formats and media (whether known now or created in the future), to i) publish, reproduce, distribute, display and store the Contribution, ii) translate the Contribution into other languages, create adaptations, reprints, include within collections and create summaries, extracts and/or, abstracts of the Contribution and convert or allow conversion into any format including without limitation audio, iii) create any other derivative work(s) based in whole or part on the on the Contribution, iv) to exploit all subsidiary rights to exploit all subsidiary rights that currently exist or as may exist in the future in the Contribution, v) the inclusion of electronic links from the Contribution to third party material wherever it may be located; and, vi) licence any third party to do any or all of the above. All research articles will be made available on an open access basis (with authors being asked to pay an open access fee—see http://www.bmj.com/about-bmj/resources-authors/forms-policies-and-checklists/copyright-open-access-and-permission-reuse). The terms of such open access shall be governed by a Creative Commons licence—details as to which Creative Commons licence will apply to the research article are set out in our worldwide licence referred to above.

Transparency declaration: The lead author affirms that this manuscript is an honest, accurate, and transparent account of the study being reported; that no important aspects of

For peer review only

the study have been omitted; and that any discrepancies from the study as planned (and, if relevant, registered) have been explained.

Dissemination statement: We are planning to disseminate our results to patient groups using social media.

Data sharing statement: The data used in this study are available in the SAIL databank at Swansea University, Swansea, UK. All proposals to use SAIL data are subject to review by an independent Information Governance Review Panel (IGRP). Before any data can be accessed, approval must be given by the IGRP.

The IGRP gives careful consideration to each project to ensure proper and appropriate use of SAIL data. When access has been granted, it is gained through a privacy-protecting safe haven and remote access system referred to as the SAIL Gateway. SAIL has established an application process to be followed by anyone who would like to access data via SAIL https://www.saildatabank.com/application-process

For peer review only

For peer review only - http://bmjopen.bmj.com/site/about/guidelines.xhtml

References

1. Cecil RLF, Goldman L, Bennett JC. Cecil Textbook of Medicine: W.B. Saunders 2000.
2. Mulvey MR, Macfarlane GJ, Beasley M, et al. Modest Association of Joint Hypermobility With Disabling and Limiting Musculoskeletal Pain: Results From a Large-Scale General Population-Based Survey. *Arthritis Care & Research* 2013;65(8):1325-33. doi: 10.1002/acr.21979
3. Commission of the European Communities Communication from the Commission to the European Parliament, the Council, the European Economic and Social Committee and the Committee of the regions on Rare Diseases: Europe's challenges. Brussels: Commission of the European Communities, 2008.
4. Søborg M-LK, Leganger J, Mortensen LQ, et al. Establishment and baseline characteristics of a nationwide Danish cohort of patients with Ehlers–Danlos syndrome. *Rheumatology (Oxford)* 2017;56(5):763-67. doi: 10.1093/rheumatology/kew478
5. Cederlöf M, Larsson H, Lichtenstein P, et al. Nationwide population-based cohort study of psychiatric disorders in individuals with Ehlers-Danlos syndrome or hypermobility syndrome and their siblings. *Bmc Psychiatry* 2016;16 doi: 10.1186/s12888-016-0922-6
6. Hakim AJ, Grahame R. A simple questionnaire to detect hypermobility: An adjunct to the assessment of patients with diffuse musculoskeletal pain. *International Journal of Clinical Practice* 2003;57(3):163-66.
7. Tinkle B, Castori M, Berglund B, et al. Hypermobile Ehlers-Danlos Syndrome (a.k.a. Ehlers-Danlos Syndrome Type III and Ehlers-Danlos Syndrome Hypermobility Type): Clinical Description and Natural History. *American Journal of Medical Genetics Part C-Seminars in Medical Genetics* 2017;175(1):48-69. doi: 10.1002/ajmg.c.31538
8. Gazit Y, Jacob G, Grahame R. Ehlers-Danlos Syndrome-Hypermobility Type: A Much Neglected Multisystemic Disorder. *Rambam Maimonides Medical Journal* 2016;7(4) doi: 10.5041/rmmj.10261
9. De Paepe A, Malfait F. The Ehlers-Danlos syndrome, a disorder with many faces. *Clinical Genetics* 2012;82(1):1-11. doi: 10.1111/j.1399-0004.2012.01858.x
10. Hakim A, O'Callaghan C, De Wandele I, et al. Cardiovascular Autonomic Dysfunction in Ehlers-Danlos Syndrome-Hypermobile Type. *American Journal of Medical Genetics Part C-Seminars in Medical Genetics* 2017;175(1):168-74. doi: 10.1002/ajmg.c.31543
11. Fikree A, Chelimsky G, Collins H, et al. Gastrointestinal Involvement in the Ehlers-Danlos Syndromes. *American Journal of Medical Genetics Part C-Seminars in Medical Genetics* 2017;175(1):181-87. doi: 10.1002/ajmg.c.31546
12. Bulbena A, Baeza-Velasco C, Bulbena-Cabre A, et al. Psychiatric and Psychological Aspects in the Ehlers-Danlos Syndromes. *American Journal of Medical Genetics Part C-Seminars in Medical Genetics* 2017;175(1):237-45. doi: 10.1002/ajmg.c.31544
13. Hakim A, De Wandele I, O'Callaghan C, et al. Chronic Fatigue in Ehlers-Danlos Syndrome-Hypermobile Type. *American Journal of Medical Genetics Part C-Seminars in Medical Genetics* 2017;175(1):175-80. doi: 10.1002/ajmg.c.31542
14. Chopra P, Tinkle B, Hamonet C, et al. Pain Management in the Ehlers-Danlos Syndromes. *American Journal of Medical Genetics Part C-Seminars in Medical Genetics* 2017;175(1):212-19. doi: 10.1002/ajmg.c.31554
15. Veit-Rubin N, Cartwright R, Singh AU, et al. Association between joint hypermobility and pelvic organ prolapse in women: a systematic review and meta-analysis. *International Urogynecology Journal* 2016;27(10):1469-78. doi: 10.1007/s00192-015-2896-1
16. Hugon-Rodin J, Lebegue G, Becourt S, et al. Gynecologic symptoms and the influence on reproductive life in 386 women with hypermobility type ehlers-danlos syndrome: a

For peer review only - http://bmjopen.bmj.com/site/about/guidelines.xhtml

cohort study. *Orphanet Journal of Rare Diseases* 2016;11 doi: 10.1186/s13023-016-0511-2

17. Pezaro S, Pearce G, Reinhold E. Hypermobile Ehlers-Danlos Syndrome during pregnancy, birth and beyond. *British Journal of Midwifery* 2018;26(4):217-23. doi: 10.12968/bjom.2018.26.4.217
18. Seneviratne SL, Maitland A, Afrin L. Mast Cell Disorders in Ehlers-Danlos Syndrome. *American Journal of Medical Genetics Part C-Seminars in Medical Genetics* 2017;175(1):226-36. doi: 10.1002/ajmg.c.31555
19. Cheung I, Vadas P. A New Disease Cluster: Mast Cell Activation Syndrome, Postural Orthostatic Tachycardia Syndrome, and Ehlers-Danlos Syndrome. *Journal of Allergy and Clinical Immunology* 2015;135(2):AB65-AB65.
20. Mantle D, Wilkins RM, Preedy V. A novel therapeutic strategy for Ehlers-Dantos syndrome based on nutritional supplements. *Medical Hypotheses* 2005;64(2):279-83. doi: 10.1016/j.mehy.2004.07.023
21. Castori M, Morlino S, Pascolini G, et al. Gastrointestinal and Nutritional Issues in Joint Hypermobility Syndrome/Ehlers-Danlos Syndrome, Hypermobility Type. *American Journal of Medical Genetics Part C-Seminars in Medical Genetics* 2015;169(1):54-75. doi: 10.1002/ajmg.c.31431
22. Terry RH, Palmer ST, Rimes KA, et al. Living with joint hypermobility syndrome: patient experiences of diagnosis, referral and self-care. *Family Practice* 2015;32(3):354-58. doi: 10.1093/fampra/cmv026
23. EURORDIS AKFF. The Voice of 12,000 Patients. Experiences and Expectations of Rare Disease Patients on Diagnosis and Care in Europe: Eurordis 2009.
24. Holman CDJ, Bass AJ, Rosman DL, et al. A decade of data linkage in Western Australia: strategic design, applications and benefits of the WA data linkage system. *Australian Health Review* 2008;32(4):766-77.
25. Cooksey R, Husain MJ, Brophy S, et al. The Cost of Ankylosing Spondylitis in the UK Using Linked Routine and Patient-Reported Survey Data. *Plos One* 2015;10(7) doi: 10.1371/journal.pone.0126105
26. Lyons RA, Jones KH, John G, et al. The SAIL databank: linking multiple health and social care datasets. *Bmc Medical Informatics and Decision Making* 2009;9 doi: 10.1186/1472-6947-9-3
27. Chisholm J. The READ Clinical Classification. *Brit Med J* 1990;300(6732):1092-92.
28. World Health Organization. ICD-10: International statistical classification of diseases and related health problems: World Health Organization 2016.
29. R: A Language and Environment for Statistical Computing [program]. Vienna, Austria: R Foundation for Statistical Computing, 2017.
30. Adib N, Davies K, Grahame R, et al. Joint hypermobility syndrome in childhood. A not so benign multisystem disorder? *Rheumatology* 2005;44(6):744-50. doi: 10.1093/rheumatology/keh557
31. Grahame R, Hakim AJ. Joint Hypermobility Syndrome is highly prevalent in general rheumatology clinics, its occurrence and clinical presentation being gender, age and race-related. *Annals of the Rheumatic Diseases* 2006;65:263-63.
32. Castori M. Ehlers-Danlos Syndrome(s) Mimicking Child Abuse: Is There an Impact on Clinical Practice? (vol 169, pg 289, 2015). *American Journal of Medical Genetics Part A* 2016;170(7):1947-47. doi: 10.1002/ajmg.a.37733
33. Association for Improvements in Maternity Services (AIMS). The Child Protection System in England: Written evidence submitted by the Association for Improvements in the Maternity Services (AIMS) 2011 [Available from: http://www.publications.parliament.uk/pa/cm201012/cmselect/cmeduc/writev/1514/cp29.htm accessed 14/02/2019.
34. Berglund B, Anne-Cathrine M, Randers I. Dignity not fully upheld when seeking health care: Experiences expressed by individuals suffering from Ehlers–Danlos syndrome. *Disability and Rehabilitation* 2010;32(1):1-7. doi: 10.3109/09638280903178407

For peer review only - http://bmjopen.bmj.com/site/about/guidelines.xhtml

35. Scheper MC, Nicholson LL, Adams RD, et al. The natural history of children with joint hypermobility syndrome and Ehlers-Danlos hypermobility type: a longitudinal cohort study. *Rheumatology* 2017;56(12):2073-83. doi: 10.1093/rheumatology/kex148

36. Engelbert RHH, Juul-Kristensen B, Pacey V, et al. The Evidence-Based Rationale for Physical Therapy Treatment of Children, Adolescents, and Adults Diagnosed With Joint Hypermobility Syndrome/Hypermobile Ehlers Danlos Syndrome. *American Journal of Medical Genetics Part C-Seminars in Medical Genetics* 2017;175(1):158-67. doi: 10.1002/ajmg.c.31545

37. Ericson WB, Wolman R. Orthopaedic Management of the Ehlers-Danlos Syndromes. *American Journal of Medical Genetics Part C-Seminars in Medical Genetics* 2017;175(1):188-94. doi: 10.1002/ajmg.c.31551

38. Soborg MLK, Leganger J, Rosenberg J, et al. Increased Need for Gastrointestinal Surgery and Increased Risk of Surgery-Related Complications in Patients with Ehlers-Danlos Syndrome: A Systematic Review. *Digestive Surgery* 2017;34(2):161-70. doi: 10.1159/000449106

39. Sobey G. Ehlers-Danlos syndrome: how to diagnose and when to perform genetic tests. *Archives of Disease in Childhood* 2015;100(1):57-61. doi: 10.1136/archdischild-2013-304822

40. Grahame R, Bird HA, Child A, et al. The revised (Brighton 1998) criteria for the diagnosis of benign joint hypermobility syndrome (BJHS). *Journal of Rheumatology* 2000;27(7):1777-79.

41. Beighton P, De Paepe A, Steinmann B, et al. Ehlers-Danlos syndromes: Revised nosology, Villefranche, 1997. *American Journal of Medical Genetics* 1998;77(1):31-37. doi: 10.1002/(sici)1096-8628(19980428)77:1<31::aid-ajmg8>3.0.co;2-o

42. Grahame R. Joint hypermobility and genetic collagen disorders: are they related? *Archives of Disease in Childhood* 1999;80(2):188-91.

43. Malfait F, Francomano C, Byers P, et al. The 2017 International Classification of the Ehlers-Danlos Syndromes. *American Journal of Medical Genetics Part C-Seminars in Medical Genetics* 2017;175(1):8-26. doi: 10.1002/ajmg.c.31552

44. Castori M, Dordoni C, Morlino S, et al. Spectrum of Mucocutaneous Manifestations in 277 Patients with Joint Hypermobility Syndrome/Ehlers-Danlos Syndrome, Hypermobility Type. *American Journal of Medical Genetics Part C-Seminars in Medical Genetics* 2015;169(1):43-53. doi: 10.1002/ajmg.c.31425

For peer review only

For peer review only - http://bmjopen.bmj.com/site/about/guidelines.xhtml

Table 1: Clinical coding for Ehlers-Danlos Syndromes and Joint Hypermobility Syndrome.

Read code descriptions (based on pre-1997 nomenclature)	EDS type according to the Villefranche Criteria	EDS Type according to the March 2017 Criteria	Read code version 2	ICD10 code
Ehlers-Danlos syndrome			PGy2.	Q79.6
Ehlers-Danlos syndrome type I	Classical type	Classical EDS	PGy20	
Ehlers-Danlos syndrome type II			PGy21	
Ehlers-Danlos syndrome type III	Hypermobility type	Hypermobile EDS or Hypermobility Spectrum Disorder	PGy22	
Ehlers-Danlos syndrome type IV	Vascular type	Vascular EDS	PGy23	
Ehlers-Danlos syndrome type V	X-linked type	No longer classified as EDS	PGy24	
Ehlers-Danlos syndrome type VI	Kyphoscoliotic type	Kyphoscoliotic EDS	PGy25	
Ehlers-Danlos syndrome type VII	Arthrochalasia type Dermatosparaxis type	Arthrochalasia EDS Dermatosparaxis EDS	PGy26	
Ehlers-Danlos syndrome type VIII	Periodontitis type	Periodontal EDS	PGy27	
Hypermobility Syndrome (JHS according to the Brighton Criteria)	(Hypermobility type)	Hypermobile EDS or Hypermobility Spectrum Disorder	N235.	728.5

For peer review only - http://bmjopen.bmj.com/site/about/guidelines.xhtml

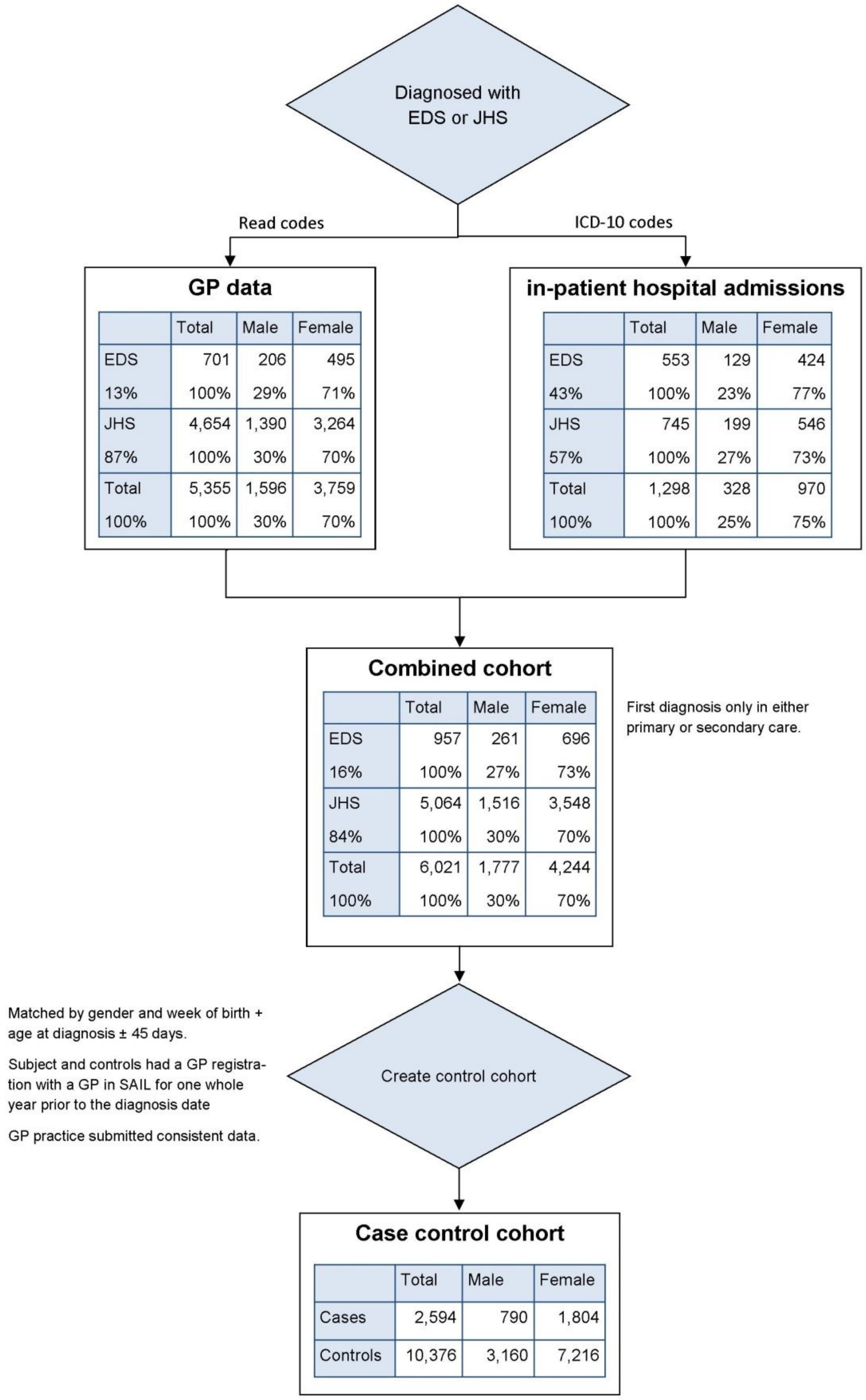

Figure 1: Flow diagram of EDS/JHS cohort and case-control cohort creation

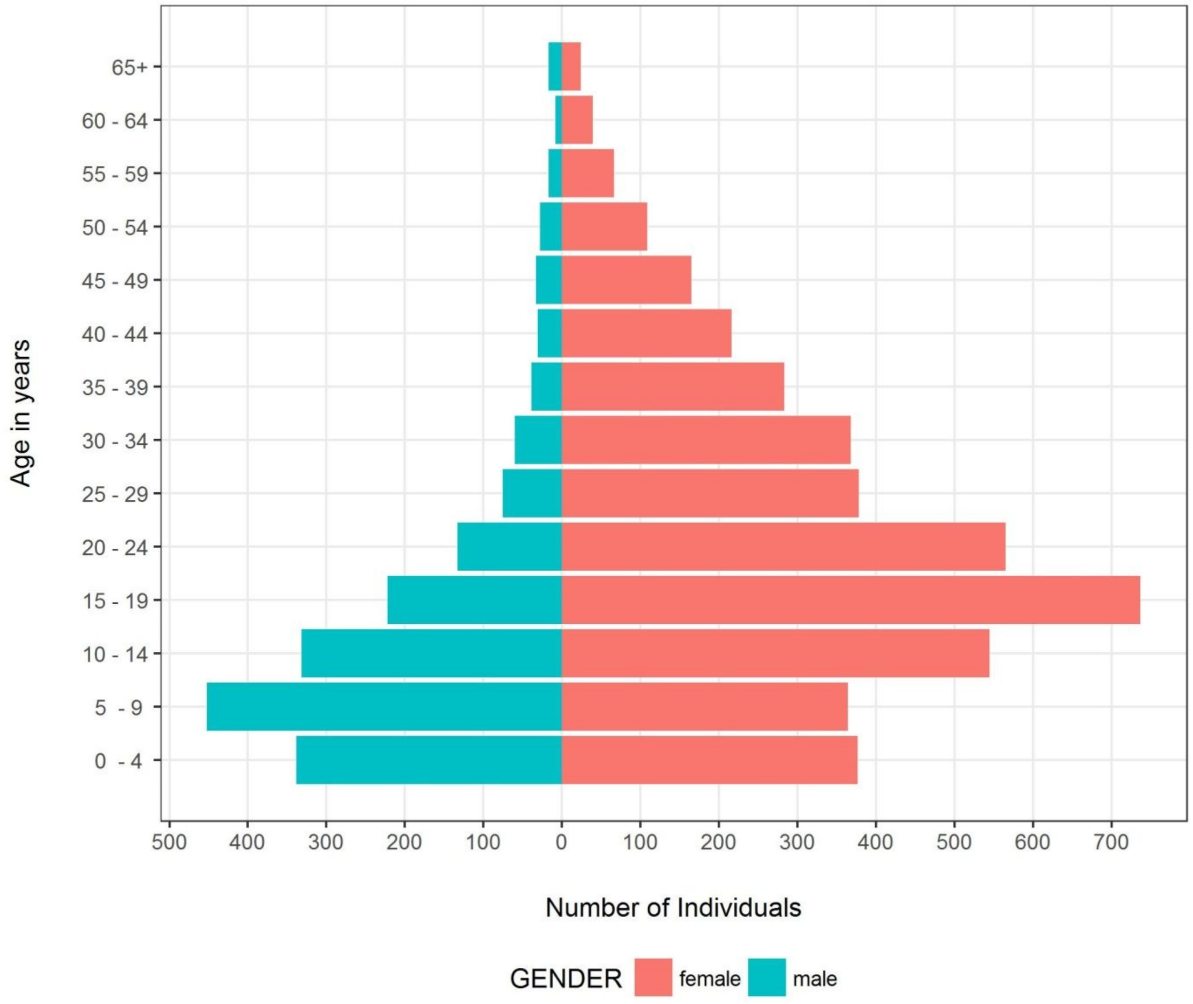

Figure 2: Age at first coded diagnosis of EDS/JHS by age group and gender

For peer review only - http://bmjopen.bmj.com/site/about/guidelines.xhtml

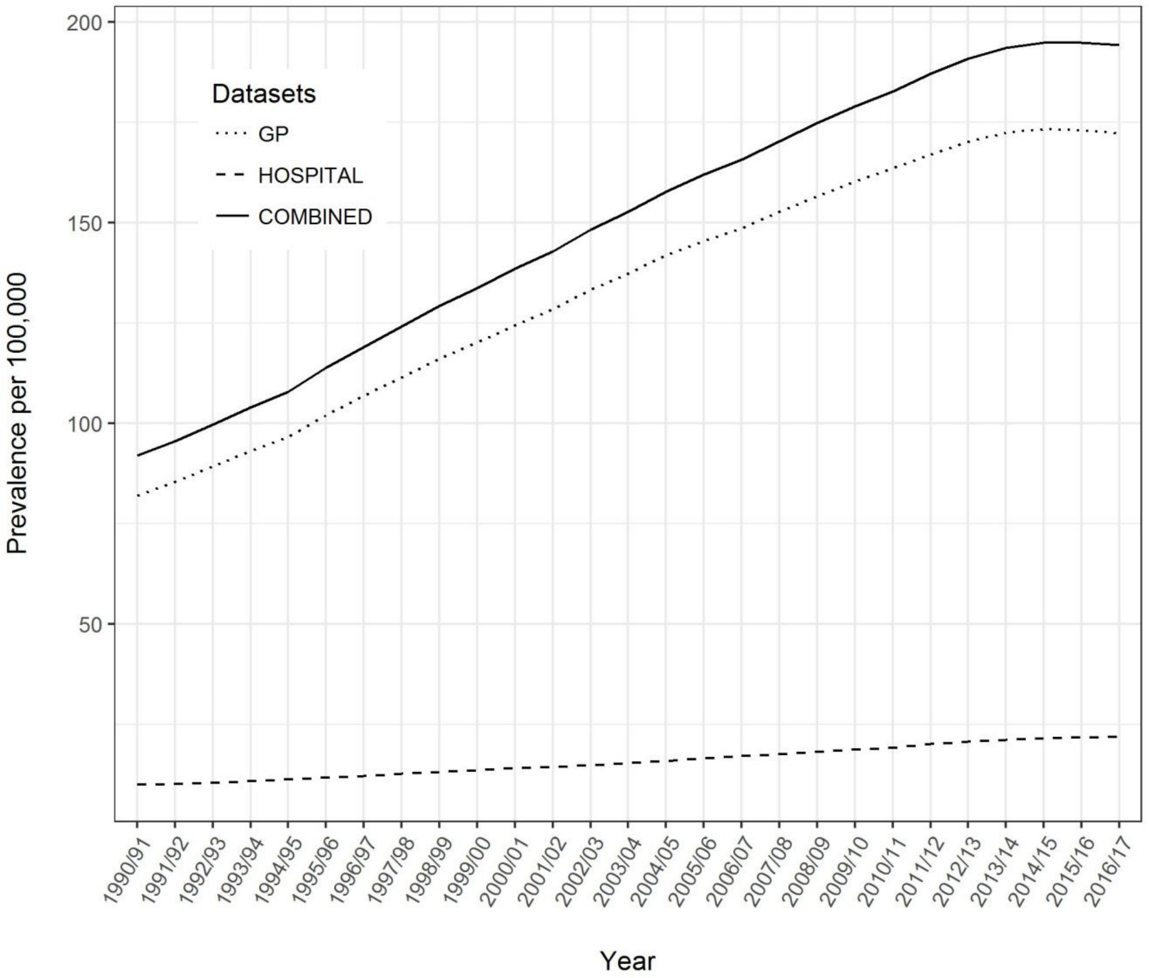

Figure 3: Prevalence of coded diagnosis of JHS/EDS in Primary Care, hospital inpatient and combined over time.

For peer review only - http://bmjopen.bmj.com/site/about/guidelines.xhtml

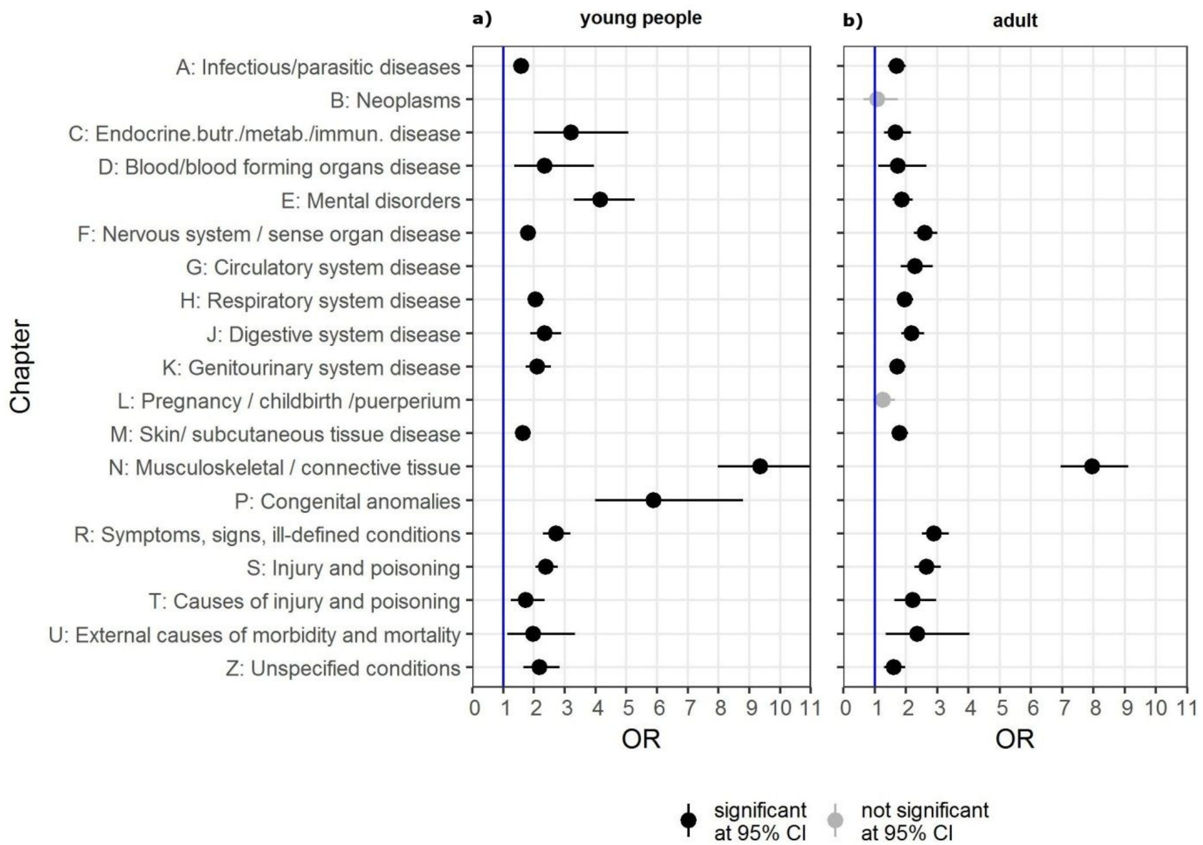

Figure 4: Odds ratios of Read chapter diagnoses for (a) young people (<18 years of age) and (b) adults (>= 18 years of age) within 12 months before and after EDS/JHS diagnosis. Presented are all results that affect at least 5 cases or 20 controls (perinatal conditions, chapter Q, are not shown as neither young people nor adults had the required minimum number of cases/controls).

For peer review only - http://bmjopen.bmj.com/site/about/guidelines.xhtml

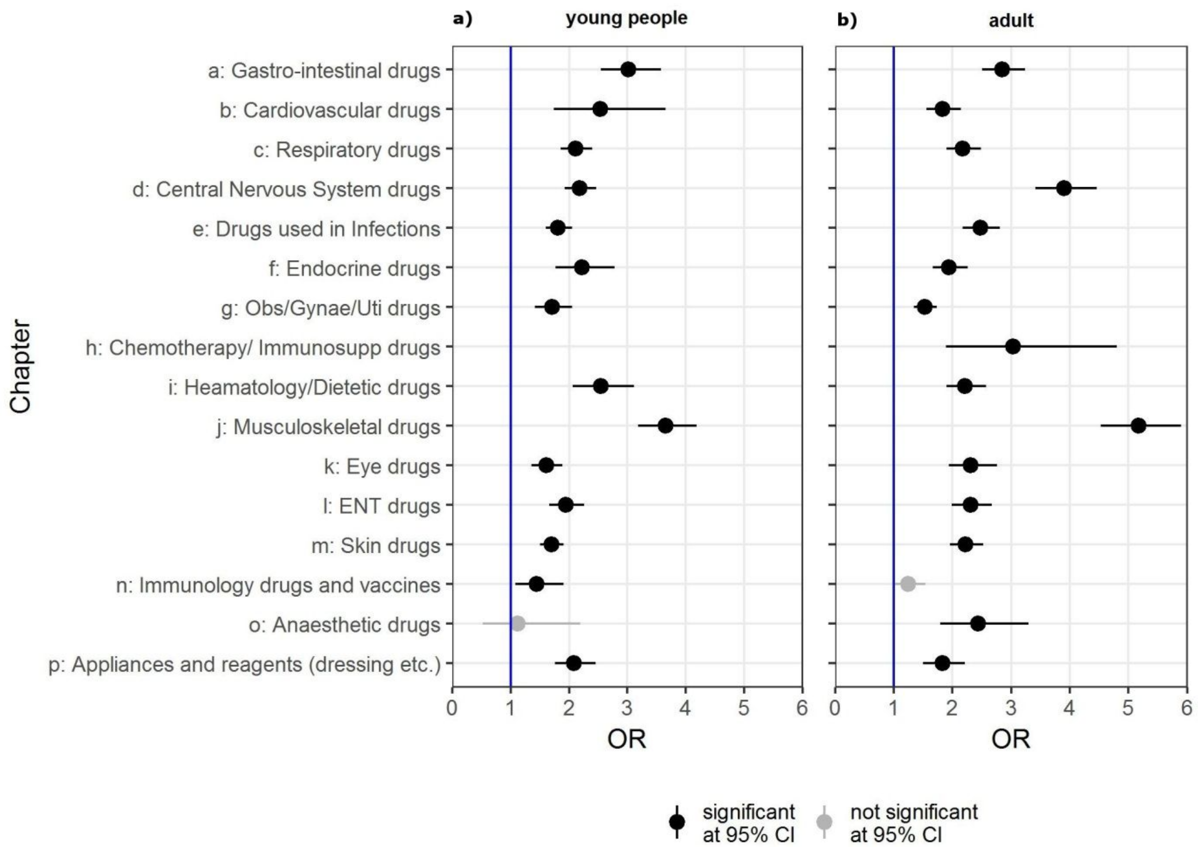

Figure 5: Odds ratios of Read chapter prescriptions for young people (<18 years of age) and adults (>= 18 years of age) within 12 months before and after EDS/JHS diagnosis. Presented are all results that affect at least 5 cases or 20 controls (incontinence and stoma appliances, chapters q and s, are not shown as neither young people nor adults had the required minimum number of cases/results).

For peer review only - http://bmjopen.bmj.com/site/about/guidelines.xhtml

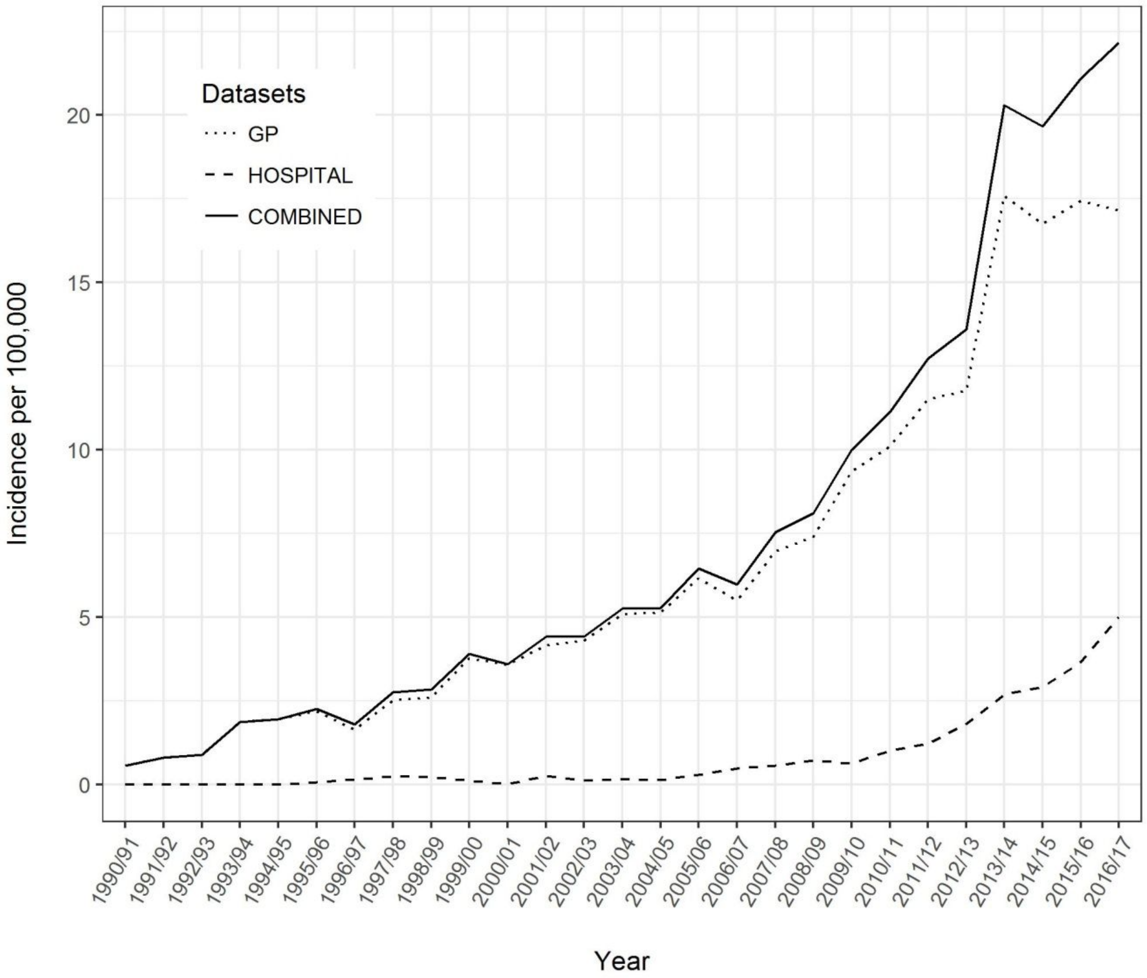

Supplement Figure 1: Incidence of diagnosis of JHS/EDS in GP, hospital inpatient and combined data over time.

For peer review only - http://bmjopen.bmj.com/site/about/guidelines.xhtml

Textbox 1

Ehlers Danlos Syndrome Nomenclature

- Joint hypermobility *per se* is reasonably common and thought to be present in around 10% of the general UK population [39].
- The Brighton criteria were used to diagnose Joint Hypermobility Syndrome from 1998 [40],
- The Villefranche criteria were applied to confirm EDS-Hypermobility Type from 1997 [41].
- Prior to the Villefranche criteria, the diagnosis EDS III was used to denote the hypermobile subtype of EDS.
- It was recognised over a number of years that JHS and EDS-HT were not distinct from one another [42].
- In March 2017 the International Consortium on the Ehlers Danlos Syndromes published a revised classification [43] naming two syndromes:
    - Hypermobile EDS (hEDS) which has narrowly defined criteria
    - Hypermobility Spectrum Disorder (HSD) for those with some but not all of the features of hEDS
- Patients who have a diagnosis of EDS-HT or JHS will fall into one of these two new categories.
- Castori et al showed that patients may move from the HSD category into hEDS over time: they also emphasised that the approach to management and the prognosis in terms of disability are the same [44]. One may therefore conclude that health needs across these groups are similar.

For peer review only

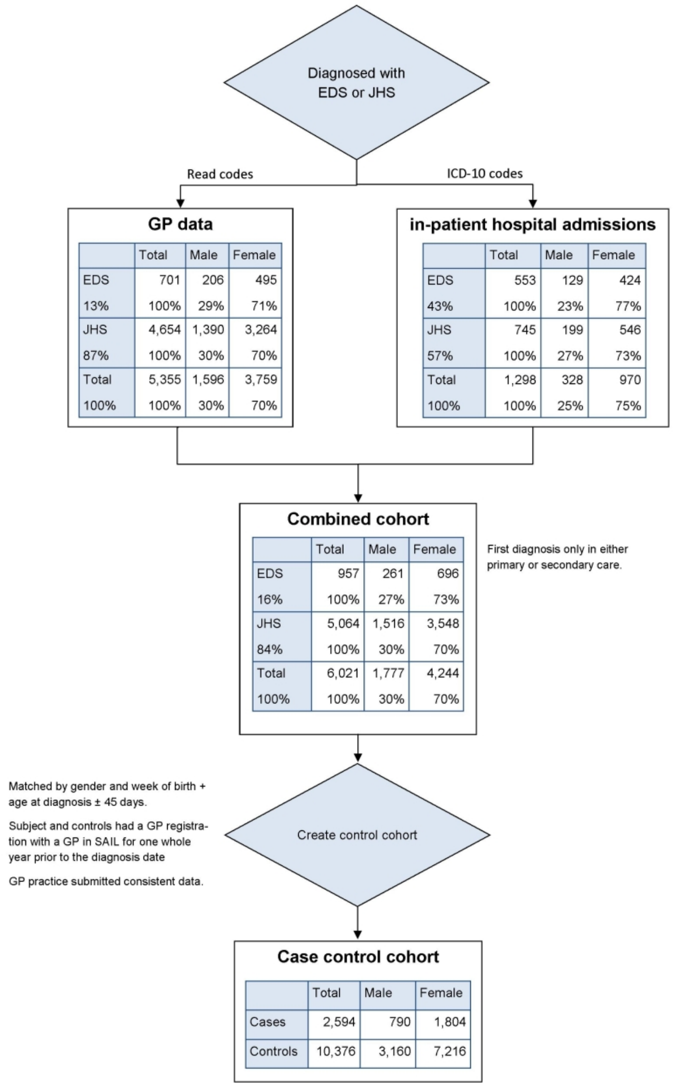

Flow diagram of EDS/JHS cohort and case-control cohort creation

209x297mm (300 x 300 DPI)

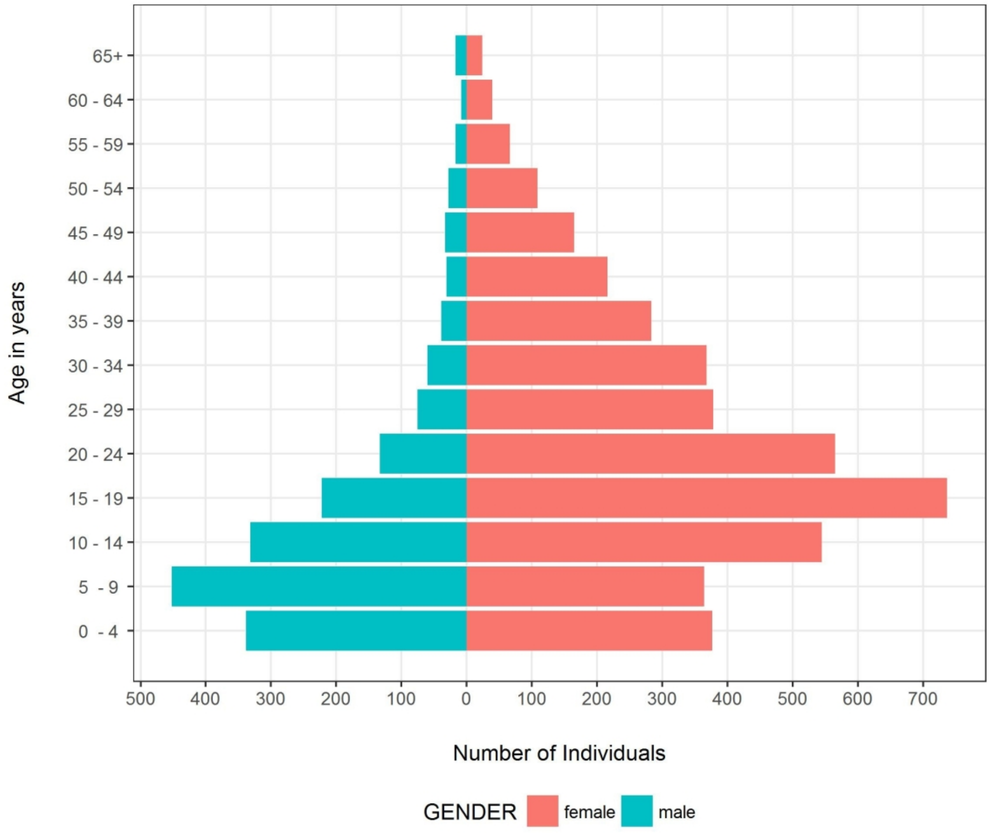

Age at first coded diagnosis of EDS/JHS by age group and gender

177x152mm (300 x 300 DPI)

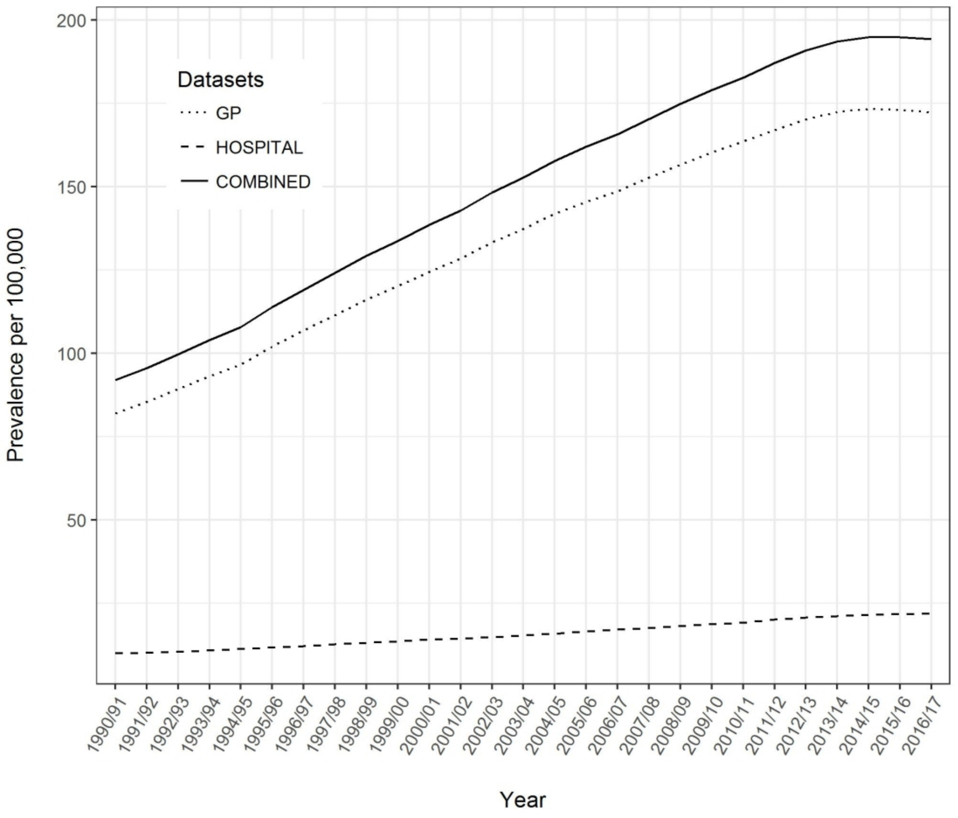

Prevalence of coded diagnosis of JHS/EDS in Primary Care, hospital inpatient and combined over time

177x152mm (300 x 300 DPI)

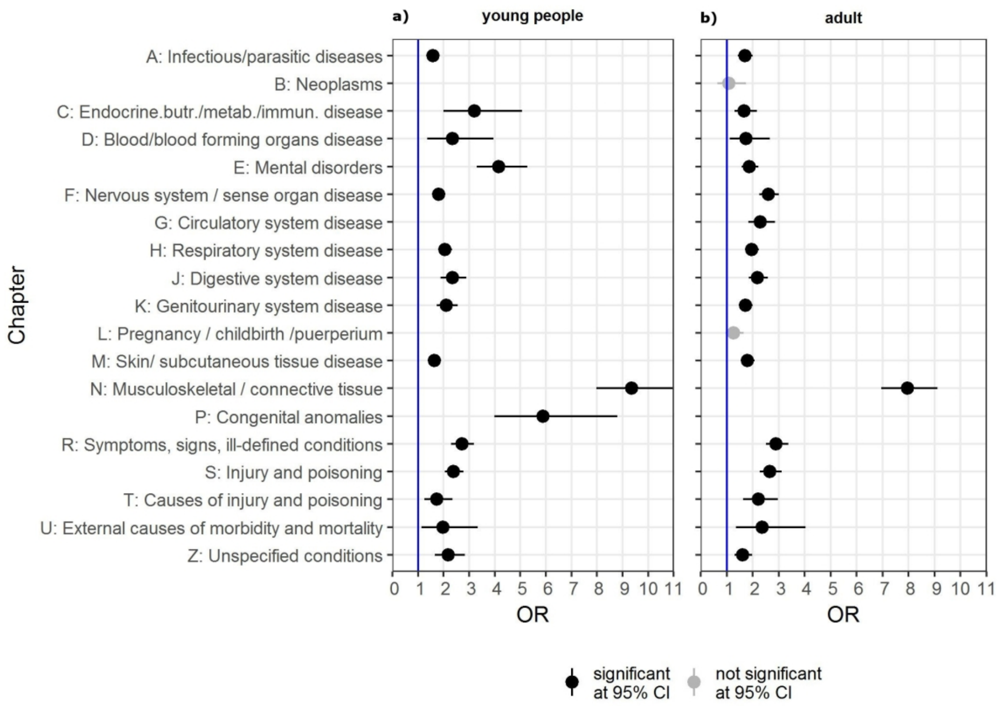

Odds ratios of Read chapter diagnoses for (a) young people (<18 years of age) and (b) adults (>= 18 years of age) within 12 months before and after EDS/JHS diagnosis. Presented are all results that affect at least 5 cases or 20 controls (perinatal conditions, chapter Q, are not shown as neither young people nor adults had the required minimum number of cases/controls)

177x127mm (300 x 300 DPI)

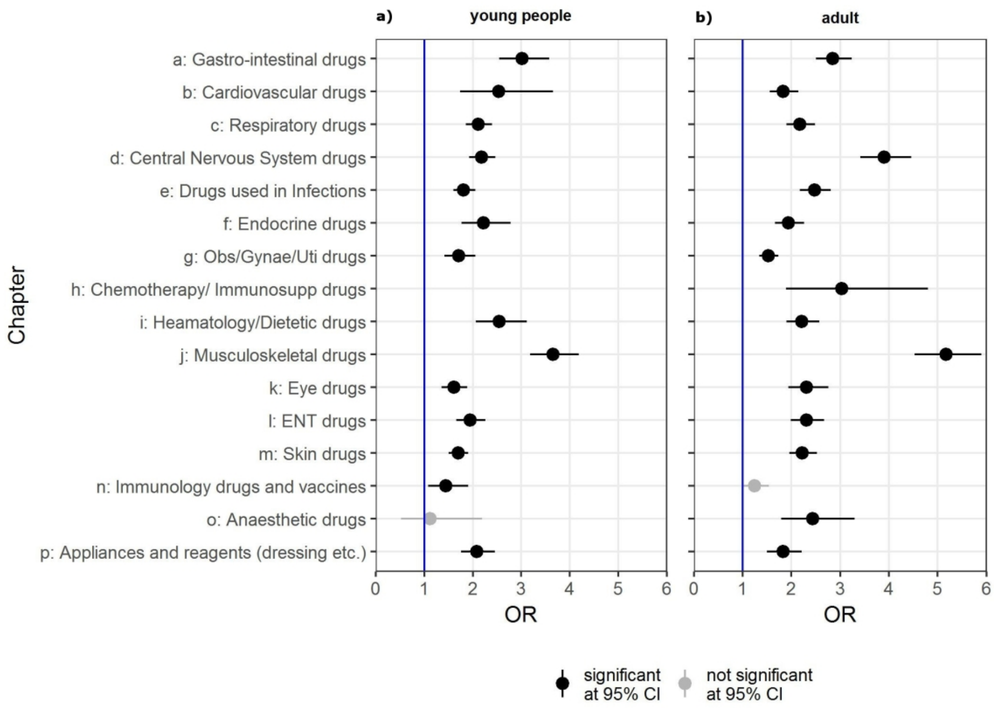

Odds ratios of Read chapter prescriptions for young people (<18 years of age) and adults (>= 18 years of age) within 12 months before and after EDS/JHS diagnosis. Presented are all results that affect at least 5 cases or 20 controls (incontinence and stoma appliances, chapters q and s, are not shown as neither young people nor adults had the required minimum number of cases/results)

177x127mm (300 x 300 DPI)

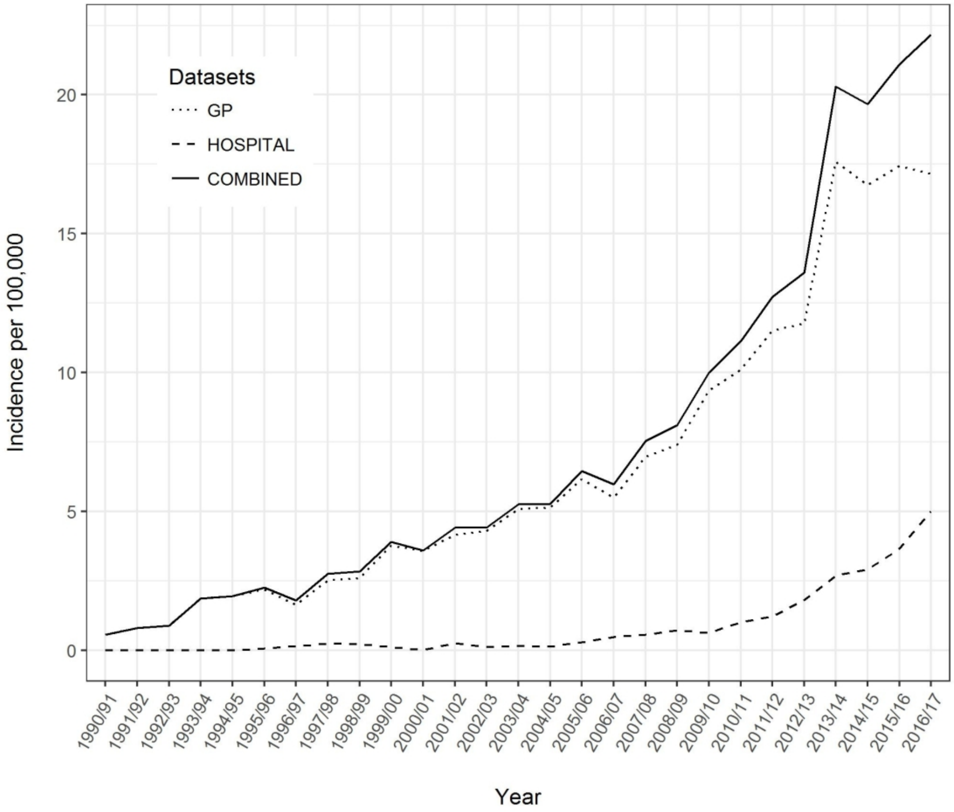

177x152mm (300 x 300 DPI)

The RECORD statement – checklist of items, extended from the STROBE statement, that should be reported in observational studies using routinely collected health data.

	Item No.	STROBE items	Location in manuscript where items are reported	RECORD items	Location in manuscript where items are reported
**Title and abstract**					
	1	(a) Indicate the study's design with a commonly used term in the title or the abstract (b) Provide in the abstract an informative and balanced summary of what was done and what was found	Title and abstract	RECORD 1.1: The type of data used should be specified in the title or abstract. When possible, the name of the databases used should be included.<br><br>RECORD 1.2: If applicable, the geographic region and timeframe within which the study took place should be reported in the title or abstract.<br><br>RECORD 1.3: If linkage between databases was conducted for the study, this should be clearly stated in the title or abstract.	Abstract p. 2<br><br>Abstract p. 2<br><br>Abstract p. 2
**Introduction**					
Background rationale	2	Explain the scientific background and rationale for the investigation being reported			Introduction p. 5-6
Objectives	3	State specific objectives, including any prespecified hypotheses			Introduction p. 6
**Methods**					
Study Design	4	Present key elements of study design early in the paper			Methods p. 7-9
Setting	5	Describe the setting, locations, and relevant dates, including periods of recruitment, exposure, follow-up, and data collection			Methods p. 7-9
Participants	6	*(a) Cohort study* - Give the eligibility criteria, and the		RECORD 6.1: The methods of study population selection (such as codes or	Methods p. 7-9<br>Table 1

For peer review only

		sources and methods of selection of participants. Describe methods of follow-up<br>*Case-control study* - Give the eligibility criteria, and the sources and methods of case ascertainment and control selection. Give the rationale for the choice of cases and controls<br>*Cross-sectional study* - Give the eligibility criteria, and the sources and methods of selection of participants<br><br>*(b) Cohort study* - For matched studies, give matching criteria and number of exposed and unexposed<br>*Case-control study* - For matched studies, give matching criteria and the number of controls per case		algorithms used to identify subjects) should be listed in detail. If this is not possible, an explanation should be provided.<br><br>RECORD 6.2: Any validation studies of the codes or algorithms used to select the population should be referenced. If validation was conducted for this study and not published elsewhere, detailed methods and results should be provided.<br><br>RECORD 6.3: If the study involved linkage of databases, consider use of a flow diagram or other graphical display to demonstrate the data linkage process, including the number of individuals with linked data at each stage.	 <br><br>NA, used standard clinical codes<br><br><br><br><br>Figure 1
Variables	7	Clearly define all outcomes, exposures, predictors, potential confounders, and effect modifiers. Give diagnostic criteria, if applicable.		RECORD 7.1: A complete list of codes and algorithms used to classify exposures, outcomes, confounders, and effect modifiers should be provided. If these cannot be reported, an explanation should be provided.	Methods p. 7-9 [calculated odds ratios]
Data sources/ measurement	8	For each variable of interest, give sources of data and details of methods of assessment (measurement).<br>Describe comparability of assessment methods if there is more than one group			Methods p. 7-9<br>Read codes in GP data<br>ICD-10 codes in PEDW data
Bias	9	Describe any efforts to address potential sources of bias			Methods p. 7-9<br>Case-control comparison only

For peer review only - http://bmjopen.bmj.com/site/about/guidelines.xhtml

					for years and GP practices with good data coverage
Study size	10	Explain how the study size was arrived at			Methods p. 7-9 Combined first diagnoses in GP and PEDW
Quantitative variables	11	Explain how quantitative variables were handled in the analyses. If applicable, describe which groupings were chosen, and why			Methods p. 7-9
Statistical methods	12	(a) Describe all statistical methods, including those used to control for confounding<br>(b) Describe any methods used to examine subgroups and interactions<br>(c) Explain how missing data were addressed<br>(d) *Cohort study* - If applicable, explain how loss to follow-up was addressed<br>*Case-control study* - If applicable, explain how matching of cases and controls was addressed<br>*Cross-sectional study* - If applicable, describe analytical methods taking account of sampling strategy<br>(e) Describe any sensitivity analyses			Methods p. 7-9<br>a) Simple odds ratios<br>b) Simple counts<br>c) NA (based on diagnoses)<br>d) Cohort – NA Case-control: week of birth and gender, dependant on registration with GP<br>e) NA
Data access and cleaning methods		..		RECORD 12.1: Authors should describe the extent to which the investigators had access to the database population used to create the study	Methods p 7-9

For peer review only

				population.<br><br>RECORD 12.2: Authors should provide information on the data cleaning methods used in the study.	Methods p. 7-9
Linkage		..		RECORD 12.3: State whether the study included person-level, institutional-level, or other data linkage across two or more databases. The methods of linkage and methods of linkage quality evaluation should be provided.	Methods p. 7-9
**Results**					
Participants	13	(a) Report the numbers of individuals at each stage of the study (*e.g.*, numbers potentially eligible, examined for eligibility, confirmed eligible, included in the study, completing follow-up, and analysed)<br>(b) Give reasons for non-participation at each stage.<br>(c) Consider use of a flow diagram		RECORD 13.1: Describe in detail the selection of the persons included in the study (*i.e.,* study population selection) including filtering based on data quality, data availability and linkage. The selection of included persons can be described in the text and/or by means of the study flow diagram.	Results p. 9-12<br>Figure 1
Descriptive data	14	(a) Give characteristics of study participants (*e.g.*, demographic, clinical, social) and information on exposures and potential confounders<br>(b) Indicate the number of participants with missing data for each variable of interest<br>(c) *Cohort study* - summarise follow-up time (*e.g.*, average and total amount)			a) p. 9-12<br>b) only exact matches, cannot identify missing data<br>c) NA
Outcome data	15	*Cohort study* - Report numbers of outcome events or summary measures over time<br>*Case-control study* - Report			p. 9-12<br>cohort: total number of people diagnosed in

For peer review only

		numbers in each exposure category, or summary measures of exposure<br>*Cross-sectional study* - Report numbers of outcome events or summary measures			either GP or PEDW<br><br>case-control: odds ratios
Main results	16	(a) Give unadjusted estimates and, if applicable, confounder-adjusted estimates and their precision (e.g., 95% confidence interval). Make clear which confounders were adjusted for and why they were included<br>(b) Report category boundaries when continuous variables were categorized<br>(c) If relevant, consider translating estimates of relative risk into absolute risk for a meaningful time period			p. 9-12<br>a) Simple odds ratios<br>b) Based on Read chapters<br>c) NA
Other analyses	17	Report other analyses done—e.g., analyses of subgroups and interactions, and sensitivity analyses			Young people vs. adults<br>Results p. 11
**Discussion**					
Key results	18	Summarise key results with reference to study objectives			p. 12
Limitations	19	Discuss limitations of the study, taking into account sources of potential bias or imprecision. Discuss both direction and magnitude of any potential bias		RECORD 19.1: Discuss the implications of using data that were not created or collected to answer the specific research question(s). Include discussion of misclassification bias, unmeasured confounding, missing data, and changing eligibility over time, as they pertain to the study being reported.	p. 12-13
Interpretation	20	Give a cautious overall interpretation of results considering objectives,			p. 12-13

For peer review only

		limitations, multiplicity of analyses, results from similar studies, and other relevant evidence			
Generalisability	21	Discuss the generalisability (external validity) of the study results			Discussion p. 12
**Other Information**					
Funding	22	Give the source of funding and the role of the funders for the present study and, if applicable, for the original study on which the present article is based			p. 14
Accessibility of protocol, raw data, and programming code		..		RECORD 22.1: Authors should provide information on how to access any supplemental information such as the study protocol, raw data, or programming code.	p. 16 data sharing statement

*Reference: Benchimol EI, Smeeth L, Guttmann A, Harron K, Moher D, Petersen I, Sørensen HT, von Elm E, Langan SM, the RECORD Working Committee. The REporting of studies Conducted using Observational Routinely-collected health Data (RECORD) Statement. *PLoS Medicine* 2015; in press.

*Checklist is protected under Creative Commons Attribution (CC BY) license.

For peer review only

BMJ Open

Diagnosed prevalence of Ehlers-Danlos syndrome and Hypermobility Spectrum Disorder in Wales, UK: a national electronic cohort study and case-control comparison

Journal:	*BMJ Open*
Manuscript ID	bmjopen-2019-031365.R1
Article Type:	Original research
Date Submitted by the Author:	23-Jul-2019
Complete List of Authors:	Demmler, Joanne; Swansea University, Swansea University Medical School<br>Atkinson, Mark; Swansea University, Swansea University Medical School<br>Reinhold, Emma; Royal College of General Practitioners<br>Choy, Ernest; Cardiff University, School of Medicine<br>Lyons, Ronan; Swansea University, Swansea University Medical School<br>Brophy, Sinead; Swansea University, Swansea University Medical School
**Primary Subject Heading**:	Public health
Secondary Subject Heading:	General practice / Family practice, Health informatics
Keywords:	Heritable Disorders of Connective Tissue, Ehlers-Danlos Syndromes, Joint Hypermobility Syndrome, Hypermobility Spectrum Disorder, Health Data Linkage, Prevalence

SCHOLARONE™
Manuscripts

Diagnosed prevalence of Ehlers-Danlos syndrome and Hypermobility Spectrum Disorder in Wales, UK: a national electronic cohort study and case-control comparison

Joanne C Demmler, Mark D Atkinson, Emma J Reinhold, Ernest Choy, Ronan A Lyons, Sinead T Brophy

Joanne C Demmler, Lecturer, Health Data Research UK, Swansea University, Singleton Park, Swansea SA2 8PP, UK. j.demmler@swansea.ac.uk.

Mark D Atkinson, Research Officer, Secure Anonymised Information Linkage (SAIL) Databank, Swansea University, Singleton Park, Swansea SA2 8PP, UK. m.atkinson@swansea.ac.uk.

Emma J Reinhold, RCGP Clinical Champion for the Ehlers-Danlos Syndromes, Clinical Innovation and Research Centre, Royal College of General Practitioners, 30 Euston Square, London, NW1 2FB. e.reinhold@doctors.org.uk.

Ernest Choy, Professor, Arthritis Research UK CREATE Centre, Section of Rheumatology, Division of Infection and Immunity, Cardiff University, Cardiff CF14 4YS, UK. ChoyEH@cardiff.ac.uk.

Ronan A Lyons, Professor, Health Data Research UK, Swansea University, Singleton Park, Swansea SA2 8PP, UK. r.a.lyons@swansea.ac.uk.

Sinead T Brophy, Professor, Health Data Research UK, Swansea University, Singleton Park, Swansea SA2 8PP, UK. s.brophy@swansea.ac.uk.

Correspondence to: JC Demmler j.demmler@swansea.ac.uk. Tel: 44 (0) 1792 295674

For peer review only

Abstract

Objectives: To describe the epidemiology of diagnosed Hypermobility Spectrum Disorder (HSD) and Ehlers-Danlos Syndromes (EDS) using linked electronic medical records. To examine whether these conditions remain rare and primarily affect the musculoskeletal system.

Design: Nationwide linked electronic cohort and nested case-control study

Setting: Routinely collected data from primary care and hospital admissions in Wales, UK.

Participants: People within the primary care or hospital data systems with a coded diagnosis of Ehlers-Danlos syndrome (EDS) or Joint Hypermobility Syndrome (JHS) between 01/07/1990 and 30/06/2017.

Main outcome measures: Combined prevalence of JHS and EDS in Wales. Additional diagnosis and prescription data in those diagnosed with EDS or JHS compared with matched controls.

Results: We found 6,021 individuals (male: 30%, female: 70%) with a diagnostic code of either EDS or JHS. This gives a diagnosed point prevalence of 194.2 per 100,000 in 2016/17 or roughly 10 cases in a practice of 5000 patients. There was a pronounced gender difference of 8.5 years (95% CI: 7.70 to 9.22) in the mean age at diagnosis. EDS or JHS was not only associated with high odds for other musculoskeletal diagnoses and drug prescriptions, but also with significantly higher odds of a diagnosis in other disease categories (e.g. mental health, nervous and digestive systems) and higher odds of a prescription in most disease categories (e.g. gastro-intestinal and cardiovascular drugs) within the 12 months before and after the first recorded diagnosis.

Conclusions: EDS and JHS (since March 2017 classified as EDS or HSD) have historically been considered rare diseases only affecting the musculoskeletal system and soft tissues. These data demonstrate that both of these assertions should be reconsidered.

For peer review only - http://bmjopen.bmj.com/site/about/guidelines.xhtml

Key Words: Heritable Disorders of Connective Tissue, Ehlers-Danlos Syndromes, Joint Hypermobility Syndrome, Hypermobility Spectrum Disorder, Health Data Linkage, Prevalence

Word Count: 3687

Strengths and Limitations

- Large cohort and nested case-control studies based on whole population routinely collected health data from primary and secondary care
- We are unable to quantify how many people are suffering from hypermobile EDS (hEDS) or HSD but remain undiagnosed, nor can we make any statement on the reliability of the diagnoses
- Although we only compared codes at Read chapter level all diagnoses and prescriptions can be matched to conditions found in the EDS/JHS literature

For peer review only

Introduction

The Ehlers-Danlos Syndromes (EDS) are a group of hereditary connective tissue disorders which mainly affect collagen. The nomenclature of these conditions has undergone a number of iterations which makes discussion of their prevalence complicated (see Textbox 1).

For many decades, studies have quoted a prevalence rate of 1 in 5000 for EDS, although the origin of this figure is unclear, seeming to appear first in a medical textbook [1 2] as an unreferenced "reasonable estimate". Thus, these syndromes have long been categorised as rare diseases, defined in the European Union as those affecting fewer than 50 in 100,000 people [3]. Kulas Søborg et al. [4] recently reported a prevalence of 20 per 100,000 for EDS in a nationwide Danish cohort based on secondary health care data up to 2012, but importantly, this data did not include patients who had received the considerably more common JHS diagnosis, now included in the latest revised classification. It is possible to extrapolate a combined population prevalence figure for JHS and EDS for Sweden [5] of around 120 per 100,000 from a study focussing on comorbid mental health issues, but no investigators have thus far set out to investigate the combined diagnosed prevalence of JHS/EDS within a population.

Although common features of these conditions are arthralgia, soft tissue injury and joint instability [6], over the last two decades it has become clear that their clinical features are not limited to musculoskeletal and cutaneous involvement, but are multisystemic [7-9]. In the special edition of the American Journal of Medical Genetics dedicated to EDS in March 2017, papers covered links to cardiovascular autonomic [10] and gastrointestinal dysfunction [11] as well as psychiatric and neurodevelopmental disorders [5 12]. Chronic disabling fatigue [13] and pain syndromes [14] were also recognised as common and multifactorial issues. Gynaecological [15 16] and obstetric [17] issues are also reported in this population. There is also an emerging link with the potentially life-threatening condition of Mast Cell Activation

For peer review only - http://bmjopen.bmj.com/site/about/guidelines.xhtml

Syndrome [18 19]. There is some emerging evidence hinting that nutritional deficiencies [20 21] may play a key role, both seeming to be more prevalent in these patients and possibly implicated in the development of some of the complications.

Early diagnosis is found to be crucial to patients [22] to enable the provision of appropriate treatment, as well as to prevent later onset complications [7]. Establishing the diagnosis of EDS/HSD is often problematic for patients, which interferes with the early detection, treatment and prevention of further escalations of recognised symptoms, disability and more elaborate complications. A mean of 14 years elapses between the first clinical manifestations and the actual diagnosis [23]. For 25% of patients this delay lasts over 28 years [23]. “A misdiagnosis was given to 56% of patients [resulting in] inappropriate treatment in 70% of the patients… For 86% of the patients, the delay in diagnosis was considered responsible for deleterious consequences.” [23(p.137)]

It is possible that some of these difficulties arise from the widespread belief amongst clinicians that EDS is rare. It is therefore of clinical importance to establish better estimates of current prevalence. Conventional studies tend to be based in restricted clinical settings, such as rheumatology clinics, and are therefore limited by the number of recruited patients and biased by severity/type of patients referred. It has been shown that using linked health data is an economic and effective alternative to performing *de novo* longitudinal studies, including rare conditions [24 25]. We used routinely held data from primary and secondary care sources to examine the epidemiology of people with a diagnostic code for EDS/JHS in Wales. We then conducted a nested case- control study to study the number of diagnoses across all body/disease systems and prescription usage in order to test the widespread belief that these conditions are primarily musculoskeletal in nature, rather than multi-system disorders.

Methods

For peer review only - http://bmjopen.bmj.com/site/about/guidelines.xhtml

Study design: Nationwide electronic cohort study

Anonymised record linkage and hosting is carried out in the Secure Anonymised Information Linkage (SAIL) databank [26] on routinely collected data held in health and social care datasets. All data within the SAIL gateway are treated in accordance with the Data Protection Act 2018 and complies with GDPR.

We used data from a variety of datasets between 01/07/1990 and 30/06/2017 to create the anonymised e-cohort and case control studies. The Primary Care data covers about 80% of all coded information held by General Practitioners (GPs) in Wales. The Welsh Demographics Service (WDS) contains key statistics, such as gender, week of birth, date of death and practice migration status for everyone in Wales registered with a GP. The Patient Episode Database for Wales (PEDW) contains all inpatient hospital admissions to a Welsh hospital. WDS and PEDW data are available for the whole of Wales. The SAIL databank enables the anonymised matching of individuals across these different datasets using a person level anonymised linkage field (ALF) [26].

Cohort preparation

We identified Welsh residents with a Read Version 2 [27] diagnostic code of EDS or JHS in primary care data or ICD-10 diagnostic codes [28] in secondary care data (hospital admissions) between 01/07/1990 (or the start of the dataset if later) and 30/06/2017. This date marks the end of maximum data coverage across all datasets. The EDS sub-classification in Read Version 2 contains some, but not all, of the subtypes which were in use prior to 1997 and as a result, the reliability of any subtype data must be highly questionable (see Table 1). Due to the lack of available correct sub-codes for EDS subtypes, the fact that the overwhelming majority of patients simply had the header code (86% of those coded as EDS, with a further 12% coded as hEDS), and that other EDS types are genuinely rare, all codes for EDS were combined. ICD-10 codes do not distinguish between any subtypes of EDS (see Table 1). Only ALF's with good matching status were included in the

For peer review only - http://bmjopen.bmj.com/site/about/guidelines.xhtml

study, i.e. direct match on either NHS number or on surname, first name, postcode, date of birth and gender; or fuzzy matching with a probability of >= 90%.

We created one dataset for diagnoses in the GP data and another for diagnoses in the hospital data. Both datasets were linked to the week of birth, gender and date of death information in WDS on their ALF and then combined to create a cohort of people with EDS/JHS in either GP or hospital data, identifying any duplications and keeping the earliest diagnosis date for any individual appearing in both datasets.

Analysis

Data linkage and data preparation within the SAIL databank were conducted using IBM DB2 10.5 SQL. Data were then imported into R (Version 3.4.1) [29], which was used for all statistical analyses. The mean age at first diagnosis between male and female subjects was compared and confidence intervals of the difference calculated.

The denominator of the diagnosed prevalence and incidence of EDS and JHS in secondary care was calculated based on the total number of individuals with recorded gender, registered and living in Wales between 01/07/1990 and 30/06/2017 for each full year of the study respectively. The prevalence and incidence in primary care denominator was further adjusted to include only people living in Wales and whose GP practice was contributing data to SAIL. The prevalence and incidence in primary and secondary care was then added together to create an overall estimate of the prevalence and incidence in Wales.

Case-Control Comparison

A nested case control method was used. Each case was matched to 4 controls with the same gender and similar age profiles (within 45 days of the week of birth). We implemented strict criteria for selection to the case-control cohort. Both cases and controls had to (a) have uninterrupted GP registrations for 1 year before and 1 year after the date of the relevant diagnosis (or died during follow-up); (b) be registered with a GP submitting data to SAIL either at the matching date or afterwards; (c) have been registered with a GP that

For peer review only - http://bmjopen.bmj.com/site/about/guidelines.xhtml

consistently recorded data across their patient profile. The latter avoids diagnoses that were retrospectively entered for a time period when the GP practice did not fully implement the use of electronic records (less than 10% of the data they recorded during 2009). Although this reduced the number of cases and controls we were able to analyse, it avoids data quality bias, especially during the early years of this study, when GPs were converting to the use of computer systems and databases. Controls with any type of diagnosed hereditary connective tissue disorder were excluded. Preliminary analysis of the combined cohort indicated that adjustment for deprivation was not necessary (i.e. equal distribution of people across deprivation quintiles). We then calculated odds ratios between cases and controls using Read chapters (excluding the Read codes for EDS and JHS). All results that affected at least 5 cases or 20 controls were visualised using forest plots.

Ethical approval

The study design uses anonymised data and therefore the need for ethical approval and participant consent was waived by the approving Institutional Review Board, the UK National Health Service Research Ethics Committee. The SAIL independent Information Governance Review Panel (IGRP) approved the study.

Patient and Public Involvement

Two of the authors of this paper have been diagnosed with symptomatic joint hypermobility disorders. This study used routinely collected data, we were not able to involve members of the public but will be disseminating our findings widely, including directly to patients via social media and through our links with patient organisations.

Results

EDS/JHS in Primary Care data

5,355 individuals with a diagnosis of either EDS or JHS with valid birth and gender information were identified. Of these, 4,654 (87%) had a diagnosis of JHS and 701 (13%) of

For peer review only - http://bmjopen.bmj.com/site/about/guidelines.xhtml

EDS. The Read code for the EDS subtype was only used for 136 (19%) individuals with 114 (16%) identified as EDS-Hypermobility Type and 22 (3%) as other EDS subtypes. 3,759 (70%) of those diagnosed with EDS/JHS were female (see Figure 1).

EDS/JHS in Hospital Data

A total of 1,298 individuals were found in the hospital data of whom 970 (75%) were female: 745 (57%) had a diagnosis of JHS and 553 (43%) EDS (see Figure 1).

Demographics of combined EDS/JHS cohort

5,355 (89%) of the cases could be found in the primary care data with the remainder in the hospital cohort. Combining the results from primary and secondary care led to a cohort of 6,021 distinct individuals. 5,064 (84%) were coded with JHS and 957 (16%) with EDS. 4,244 (70%) of patients were female. The age at first diagnosis peaked in the age group 5-9 years for males and 15-19 years for females (see Figure 2). There was a significant difference of 8.5 years in the mean age of diagnosis between males and females (95% CI: 7.70 to 9.22): 9.6 years in EDS (95% CI: 6.85 to 12.31) and 8.3 years in JHS (95% CI: 7.58 to 9.11). 72% of males were diagnosed during childhood (age < 18 years) in contrast to only 41% of females.

2016/17 is the latest year for which we have complete data and could therefore derive prevalence. During this year, 2,668,902 people were registered with a GP in Wales submitting data to SAIL, of whom 4,598 had a diagnostic code of EDS/JHS which first appeared in the primary care data (172 in 100,000). A further 711 people out of the 3,239,153 registered with any GP in Wales during 2016/17 had an EDS/JHS diagnosis which first appears in secondary care data (22 in 100,000). There is an increasing rate of coded diagnoses throughout the period. Assuming that the GP data is representative of the whole of Wales this leads to a combined point prevalence of 194 in 100,000 at the end of the study period. This corresponds to about 10 cases in a practice of 5000 patients (see Figure 3). The incidence of EDS/JHS over this time period is shown in Supplement Figure 1.

For peer review only - http://bmjopen.bmj.com/site/about/guidelines.xhtml

Factors associated with JHS/EDS

2,597 cases had good GP data coverage at the age of diagnosis and could be matched by age and gender with controls (see Figure 1). 1,340 cases (male: 561; female: 779) were first diagnosed before the age of 18 years and 1,254 cases (male: 229; female: 1,025) above this age. The people in the nested case-control cohort were slightly older than the overall cohort (data not shown here).

Looking at the time frame of 1 year either side of the first coded diagnosis of EDS/JHS amongst young people (age < 18 years) there were significantly more additional diagnoses in 16 out of 20 Read code disease categories compared with their controls (see Figure 4a). The top three Read diagnosis chapters with increased odds for the EDS/JHS cohort were for musculoskeletal conditions (OR 9.36, 95% CI: 7.98 to 11.00), congenital anomalies (OR 5.89; 95% CI: 3.98 to 8.80) and mental disorders (OR 4.16; 95% CI: 3.29 to 5.27).

People that were diagnosed as adults (age >= 18 years) had also significantly more diagnoses in 16 out of 20 Read code categories than their controls (see Figure 4b). The top three Read diagnosis chapters for adults with higher odds in the EDS/JHS cohort were musculoskeletal disorders (OR 7.95; 95% CI: 6.95 to 9.12), congenital anomalies (OR 5.18; 95% CI: 2.78 to 9.78) and symptoms, signs and ill-defined conditions (OR 2.9; 95% CI: 2.5 to 3.37). Circulatory system disease (OR 2.29; 95% CI: 1.83 to 2.86) and mental disorders remained significant (OR 1.87; 95% CI: 1.57 to 2.22), but not to the same extend as they were for young people.

Young people showed significantly higher odds for prescriptions in 14 out of 17 Read code categories then their controls (see Figure 5a). The top three prescriptions Read chapters with increased odds for the EDS/JHS cohort were for (i) musculoskeletal drugs (OR 3.65; 95% CI: 3.18 to 4.18), (ii) gastro-intestinal drugs (OR 3.02; 95% CI: 2.54 to 3.58) and (iii) haematology/dietetic drugs (OR 2.54; 95% CI: 2.06 to 3.11).

For peer review only - http://bmjopen.bmj.com/site/about/guidelines.xhtml

Adults had significantly higher odds of prescriptions for 15 out of 17 Read code categories (see Figure 5b). The top three prescriptions with higher odds for EDS/JHS people were for (i) musculoskeletal drugs (OR 5.17; 95% CI: 4.53 to 5.9), (ii) central nervous system drugs (OR 3.9; 95% CI: 3.41 to 4.46) and (iii) chemotherapy/immunosuppressant drugs (OR 3.03; 95% CI: 1.89 to 4.8). Gastro-intestinal drugs (OR 2.85; 95% CI: 2.5 to 3.24) and haematology/dietetic drugs (OR 2.21; 95% CI: 1.9 to 2.57) remain significant, but at slightly lower levels than in the young EDS/JHS population.

Discussion

This work examined the epidemiology of EDS and JHS and found a combined diagnosed prevalence of 194.2 per 100,000 (0.19%), or 1 in 500 people in Wales; hEDS or HSD within the 2017 classification. We found a steadily increasing rate of diagnosis over the past 27 years (see Supplement Figure 1), as well as higher rates of diagnoses for other conditions and prescriptions within 12 months (pre and post) of the recorded first diagnosis in most categories. This suggests that hEDS/HSD, when considered together, do not meet the definition of rare conditions [23], and have widespread effects across multiple body systems. It is well-known that EDS is poorly recognised in children [30 31] and initial symptoms and EDS-associated diagnoses can appear to be simply a 'normal' pattern of childhood illness when taken as an isolated event. Furthermore, children with hEDS often present with symptoms that can lead to a misdiagnosis of mental illness or consideration of child abuse [12 32]. Suspicion of abuse has been shown to be extremely damaging to the mental health of the parent(s) and can lead to an avoidance of accessing health care or other public services, such as schools [33]. The prolonged and sometimes traumatic diagnosis and/or misdiagnosis process in EDS can lead to further disengagement with services [34]. The lack of a timely diagnosis has great implications for disease management and progression and impedes the appropriate consideration of surgical interventions [7 35-38] as well as pregnancy and birth planning [17]. It is perhaps only in stepping back to look at the pattern of effects across

For peer review only - http://bmjopen.bmj.com/site/about/guidelines.xhtml

multiple body systems that practitioners might begin to consider a connective tissue disorder.

Strengths and Limitations

The strength of this study is that we were able to combine diagnostic codes from several primary and secondary health care providers to create a large cohort of individuals with EDS/JHS. We have 27 years of data with at least 11 years of very good data coverage in the key datasets, which further improves with each data update of the SAIL databank, however data coverage for the first couple of years is less comprehensive.

The majority of subjects were identified via their primary care data, which is a strength and a weakness. As 89% of cases were identified through primary care data studies not using primary care data may underestimate the prevalence of hEDS/HSD. We are unable to quantify how many people are suffering from hEDS or HSD but remain undiagnosed. However, we cannot comment on the reliability of the diagnoses in the primary care dataset. It is also likely that the majority of cases were not actually diagnosed in primary care, but their entries were created through secondary care contacts, such as outpatient appointments or musculoskeletal assessment clinics, but coded data are lacking from these sources.

Although a snapshot of Read chapters codes that are more prevalent in our JHS/EDS cohort does not allow us to look at specific diagnoses and prescriptions, they can all be matched to conditions associated with EDS/JHS in the literature, for instance pain, fatigue, cardiovascular, gastrointestinal and gynaecological disorders, dysautonomia, mast cell activation as well as urinary tract infections [7]. We hope in future work to examine in greater detail these findings of significant differences between people with hEDS/HSD and others in order that we can better understand the nature of this condition, as well as potentially improving diagnostic recognition. Having created this case-control cohort, further examination is made simpler as this first step has already been made.

For peer review only - http://bmjopen.bmj.com/site/about/guidelines.xhtml

We conclude that EDS/HSD are not rare conditions and are associated with significantly increased odds of additional diagnoses and use of medications across many body systems. There is a large gender difference in the age of diagnosis, with many women not diagnosed until adulthood. Early diagnosis, however, is crucial to patients, the administration of preventive therapies, the investigation of comorbid conditions and the overall management process. Further research is needed to understand patient pathways, comorbidities and progression of associated symptoms and diseases. Health services should be aware of these findings for the provision of training, diagnostic and treatment services for the many tens of thousands of patients living with these life-changing conditions throughout the United Kingdom and beyond.

Author contributions

JD conceived the project. SB and MA contributed to the study design and analysis plan. RAL validated clinical codes in primary care. EC validated clinical codes in secondary care. JD undertook the analysis. JD and ER carried out the literature reviews and drafted the manuscript. All authors reviewed the manuscript and approved the final version for submission.

Funding

This work was supported by The Farr Institute. The Farr Institute was supported by a 10-funder consortium: Arthritis Research UK, the British Heart Foundation, Cancer Research UK, the Economic and Social Research Council, the Engineering and Physical Sciences Research Council, the Medical Research Council, the National Institute of Health Research, the National Institute for Social Care and Health Research (Welsh Assembly Government), the Chief Scientist Office (Scottish Government Health Directorates), the Wellcome Trust, (MRC Grant No: MR/K006525/1).

This work was also supported by Health Data Research UK which receives its funding from HDR UK Ltd (NIWA1) funded by the UK Medical Research Council, Engineering and

For peer review only - http://bmjopen.bmj.com/site/about/guidelines.xhtml

Physical Sciences Research Council, Economic and Social Research Council, Department of Health and Social Care (England), Chief Scientist Office of the Scottish Government Health and Social Care Directorates, Health and Social Care Research and Development Division (Welsh Government), Public Health Agency (Northern Ireland), British Heart Foundation (BHF) and the Wellcome Trust.

The above funders played no role in the study design, in the collection, analysis, or interpretation of data; in the writing of the report; or in the decision to submit the article for publication. The researchers are independent from the funders and all authors, external and internal, had full access to all of the data in the study and take responsibility for the integrity of the data and the accuracy of the data analysis.

<u>Competing interests:</u> All authors have completed the ICMJE uniform disclosure form at www.icmje.org/coi_disclosure.pdf and declare: no support from any organisation for the submitted work; no financial relationships with any organisations that might have an interest in the submitted work in the previous three years; no other relationships or activities that could appear to have influenced the submitted work.

<u>Exclusive licence:</u> The Corresponding Author has the right to grant on behalf of all authors and does grant on behalf of all authors, a worldwide licence (http://www.bmj.com/sites/default/files/BMJ%20Author%20Licence%20March%202013.doc) to the Publishers and its licensees in perpetuity, in all forms, formats and media (whether known now or created in the future), to i) publish, reproduce, distribute, display and store the Contribution, ii) translate the Contribution into other languages, create adaptations, reprints, include within collections and create summaries, extracts and/or, abstracts of the Contribution and convert or allow conversion into any format including without limitation audio, iii) create any other derivative work(s) based in whole or part on the on the Contribution, iv) to exploit all subsidiary rights to exploit all subsidiary rights that currently

For peer review only - http://bmjopen.bmj.com/site/about/guidelines.xhtml

exist or as may exist in the future in the Contribution, v) the inclusion of electronic links from the Contribution to third party material wherever it may be located; and, vi) licence any third party to do any or all of the above. All research articles will be made available on an open access basis (with authors being asked to pay an open access fee—see http://www.bmj.com/about-bmj/resources-authors/forms-policies-and-checklists/copyright-open-access-and-permission-reuse). The terms of such open access shall be governed by a Creative Commons licence—details as to which Creative Commons licence will apply to the research article are set out in our worldwide licence referred to above.

Transparency declaration: The lead author affirms that this manuscript is an honest, accurate, and transparent account of the study being reported; that no important aspects of the study have been omitted; and that any discrepancies from the study as planned (and, if relevant, registered) have been explained.

Dissemination statement: We are planning to disseminate our results to patient groups using social media.

Data sharing statement: The data used in this study are available in the SAIL databank at Swansea University, Swansea, UK. All proposals to use SAIL data are subject to review by an independent Information Governance Review Panel (IGRP). Before any data can be accessed, approval must be given by the IGRP.

The IGRP gives careful consideration to each project to ensure proper and appropriate use of SAIL data. When access has been granted, it is gained through a privacy-protecting safe haven and remote access system referred to as the SAIL Gateway. SAIL has established an application process to be followed by anyone who would like to access data via SAIL https://www.saildatabank.com/application-process.

For peer review only

Additional resources: RCGP Clinical Toolkit on the Ehlers-Danlos Syndromes

www.rcgp.org.uk/eds; RCGP Podcast "Introduction to Ehlers-Danlos Syndromes"

https://audioboom.com/posts/6896541-introduction-to-ehlers-danlos-syndromes

References

1. Cecil RLF, Goldman L, Bennett JC. Cecil Textbook of Medicine: W.B. Saunders 2000.
2. Mulvey MR, Macfarlane GJ, Beasley M, et al. Modest Association of Joint Hypermobility With Disabling and Limiting Musculoskeletal Pain: Results From a Large-Scale General Population-Based Survey. *Arthritis Care & Research* 2013;65(8):1325-33. doi: 10.1002/acr.21979
3. Commission of the European Communities Communication from the Commission to the European Parliament, the Council, the European Economic and Social Committee and the Committee of the regions on Rare Diseases: Europe's challenges. Brussels: Commission of the European Communities, 2008.
4. Søborg M-LK, Leganger J, Mortensen LQ, et al. Establishment and baseline characteristics of a nationwide Danish cohort of patients with Ehlers–Danlos syndrome. *Rheumatology (Oxford)* 2017;56(5):763-67. doi: 10.1093/rheumatology/kew478
5. Cederlöf M, Larsson H, Lichtenstein P, et al. Nationwide population-based cohort study of psychiatric disorders in individuals with Ehlers-Danlos syndrome or hypermobility syndrome and their siblings. *Bmc Psychiatry* 2016;16 doi: 10.1186/s12888-016-0922-6
6. Hakim AJ, Grahame R. A simple questionnaire to detect hypermobility: An adjunct to the assessment of patients with diffuse musculoskeletal pain. *International Journal of Clinical Practice* 2003;57(3):163-66.
7. Tinkle B, Castori M, Berglund B, et al. Hypermobile Ehlers-Danlos Syndrome (a.k.a. Ehlers-Danlos Syndrome Type III and Ehlers-Danlos Syndrome Hypermobility Type): Clinical Description and Natural History. *American Journal of Medical Genetics Part C-Seminars in Medical Genetics* 2017;175(1):48-69. doi: 10.1002/ajmg.c.31538
8. Gazit Y, Jacob G, Grahame R. Ehlers-Danlos Syndrome-Hypermobility Type: A Much Neglected Multisystemic Disorder. *Rambam Maimonides Medical Journal* 2016;7(4) doi: 10.5041/rmmj.10261
9. De Paepe A, Malfait F. The Ehlers-Danlos syndrome, a disorder with many faces. *Clinical Genetics* 2012;82(1):1-11. doi: 10.1111/j.1399-0004.2012.01858.x
10. Hakim A, O'Callaghan C, De Wandele I, et al. Cardiovascular Autonomic Dysfunction in Ehlers-Danlos Syndrome-Hypermobile Type. *American Journal of Medical Genetics Part C-Seminars in Medical Genetics* 2017;175(1):168-74. doi: 10.1002/ajmg.c.31543
11. Fikree A, Chelimsky G, Collins H, et al. Gastrointestinal Involvement in the Ehlers-Danlos Syndromes. *American Journal of Medical Genetics Part C-Seminars in Medical Genetics* 2017;175(1):181-87. doi: 10.1002/ajmg.c.31546
12. Bulbena A, Baeza-Velasco C, Bulbena-Cabre A, et al. Psychiatric and Psychological Aspects in the Ehlers-Danlos Syndromes. *American Journal of Medical Genetics Part C-Seminars in Medical Genetics* 2017;175(1):237-45. doi: 10.1002/ajmg.c.31544

For peer review only - http://bmjopen.bmj.com/site/about/guidelines.xhtml

13. Hakim A, De Wandele I, O'Callaghan C, et al. Chronic Fatigue in Ehlers-Danlos Syndrome-Hypermobile Type. *American Journal of Medical Genetics Part C-Seminars in Medical Genetics* 2017;175(1):175-80. doi: 10.1002/ajmg.c.31542
14. Chopra P, Tinkle B, Hamonet C, et al. Pain Management in the Ehlers-Danlos Syndromes. *American Journal of Medical Genetics Part C-Seminars in Medical Genetics* 2017;175(1):212-19. doi: 10.1002/ajmg.c.31554
15. Veit-Rubin N, Cartwright R, Singh AU, et al. Association between joint hypermobility and pelvic organ prolapse in women: a systematic review and meta-analysis. *International Urogynecology Journal* 2016;27(10):1469-78. doi: 10.1007/s00192-015-2896-1
16. Hugon-Rodin J, Lebegue G, Becourt S, et al. Gynecologic symptoms and the influence on reproductive life in 386 women with hypermobility type ehlers-danlos syndrome: a cohort study. *Orphanet Journal of Rare Diseases* 2016;11 doi: 10.1186/s13023-016-0511-2
17. Pezaro S, Pearce G, Reinhold E. Hypermobile Ehlers-Danlos Syndrome during pregnancy, birth and beyond. *British Journal of Midwifery* 2018;26(4):217-23. doi: 10.12968/bjom.2018.26.4.217
18. Seneviratne SL, Maitland A, Afrin L. Mast Cell Disorders in Ehlers-Danlos Syndrome. *American Journal of Medical Genetics Part C-Seminars in Medical Genetics* 2017;175(1):226-36. doi: 10.1002/ajmg.c.31555
19. Cheung I, Vadas P. A New Disease Cluster: Mast Cell Activation Syndrome, Postural Orthostatic Tachycardia Syndrome, and Ehlers-Danlos Syndrome. *Journal of Allergy and Clinical Immunology* 2015;135(2):AB65-AB65.
20. Mantle D, Wilkins RM, Preedy V. A novel therapeutic strategy for Ehlers-Dantos syndrome based on nutritional supplements. *Medical Hypotheses* 2005;64(2):279-83. doi: 10.1016/j.mehy.2004.07.023
21. Castori M, Morlino S, Pascolini G, et al. Gastrointestinal and Nutritional Issues in Joint Hypermobility Syndrome/Ehlers-Danlos Syndrome, Hypermobility Type. *American Journal of Medical Genetics Part C-Seminars in Medical Genetics* 2015;169(1):54-75. doi: 10.1002/ajmg.c.31431
22. Terry RH, Palmer ST, Rimes KA, et al. Living with joint hypermobility syndrome: patient experiences of diagnosis, referral and self-care. *Family Practice* 2015;32(3):354-58. doi: 10.1093/fampra/cmv026
23. EURORDIS AKFF. The Voice of 12,000 Patients. Experiences and Expectations of Rare Disease Patients on Diagnosis and Care in Europe: Eurordis 2009.
24. Holman CDJ, Bass AJ, Rosman DL, et al. A decade of data linkage in Western Australia: strategic design, applications and benefits of the WA data linkage system. *Australian Health Review* 2008;32(4):766-77.
25. Cooksey R, Husain MJ, Brophy S, et al. The Cost of Ankylosing Spondylitis in the UK Using Linked Routine and Patient-Reported Survey Data. *Plos One* 2015;10(7) doi: 10.1371/journal.pone.0126105
26. Lyons RA, Jones KH, John G, et al. The SAIL databank: linking multiple health and social care datasets. *Bmc Medical Informatics and Decision Making* 2009;9 doi: 10.1186/1472-6947-9-3
27. Chisholm J. The READ Clinical Classification. *Brit Med J* 1990;300(6732):1092-92.
28. World Health Organization. ICD-10: International statistical classification of diseases and related health problems: World Health Organization 2016.
29. R: A Language and Environment for Statistical Computing [program]. Vienna, Austria: R Foundation for Statistical Computing, 2017.
30. Adib N, Davies K, Grahame R, et al. Joint hypermobility syndrome in childhood. A not so benign multisystem disorder? *Rheumatology* 2005;44(6):744-50. doi: 10.1093/rheumatology/keh557
31. Grahame R, Hakim AJ. Joint Hypermobility Syndrome is highly prevalent in general rheumatology clinics, its occurrence and clinical presentation being gender, age and race-related. *Annals of the Rheumatic Diseases* 2006;65:263-63.

For peer review only - http://bmjopen.bmj.com/site/about/guidelines.xhtml

32. Castori M. Ehlers-Danlos Syndrome(s) Mimicking Child Abuse: Is There an Impact on Clinical Practice? (vol 169, pg 289, 2015). *American Journal of Medical Genetics Part A* 2016;170(7):1947-47. doi: 10.1002/ajmg.a.37733
33. Association for Improvements in Maternity Services (AIMS). The Child Protection System in England: Written evidence submitted by the Association for Improvements in the Maternity Services (AIMS) 2011 [Available from: http://www.publications.parliament.uk/pa/cm201012/cmselect/cmeduc/writev/1514/cp29.htm accessed 14/02/2019.
34. Berglund B, Anne-Cathrine M, Randers I. Dignity not fully upheld when seeking health care: Experiences expressed by individuals suffering from Ehlers–Danlos syndrome. *Disability and Rehabilitation* 2010;32(1):1-7. doi: 10.3109/09638280903178407
35. Scheper MC, Nicholson LL, Adams RD, et al. The natural history of children with joint hypermobility syndrome and Ehlers-Danlos hypermobility type: a longitudinal cohort study. *Rheumatology* 2017;56(12):2073-83. doi: 10.1093/rheumatology/kex148
36. Engelbert RHH, Juul-Kristensen B, Pacey V, et al. The Evidence-Based Rationale for Physical Therapy Treatment of Children, Adolescents, and Adults Diagnosed With Joint Hypermobility Syndrome/Hypermobile Ehlers Danlos Syndrome. *American Journal of Medical Genetics Part C-Seminars in Medical Genetics* 2017;175(1):158-67. doi: 10.1002/ajmg.c.31545
37. Ericson WB, Wolman R. Orthopaedic Management of the Ehlers-Danlos Syndromes. *American Journal of Medical Genetics Part C-Seminars in Medical Genetics* 2017;175(1):188-94. doi: 10.1002/ajmg.c.31551
38. Soborg MLK, Leganger J, Rosenberg J, et al. Increased Need for Gastrointestinal Surgery and Increased Risk of Surgery-Related Complications in Patients with Ehlers-Danlos Syndrome: A Systematic Review. *Digestive Surgery* 2017;34(2):161-70. doi: 10.1159/000449106
39. Sobey G. Ehlers-Danlos syndrome: how to diagnose and when to perform genetic tests. *Archives of Disease in Childhood* 2015;100(1):57-61. doi: 10.1136/archdischild-2013-304822
40. Grahame R, Bird HA, Child A, et al. The revised (Brighton 1998) criteria for the diagnosis of benign joint hypermobility syndrome (BJHS). *Journal of Rheumatology* 2000;27(7):1777-79.
41. Beighton P, De Paepe A, Steinmann B, et al. Ehlers-Danlos syndromes: Revised nosology, Villefranche, 1997. *American Journal of Medical Genetics* 1998;77(1):31-37. doi: 10.1002/(sici)1096-8628(19980428)77:1<31::aid-ajmg8>3.0.co;2-o
42. Grahame R. Joint hypermobility and genetic collagen disorders: are they related? *Archives of Disease in Childhood* 1999;80(2):188-91.
43. Malfait F, Francomano C, Byers P, et al. The 2017 International Classification of the Ehlers-Danlos Syndromes. *American Journal of Medical Genetics Part C-Seminars in Medical Genetics* 2017;175(1):8-26. doi: 10.1002/ajmg.c.31552
44. Castori M, Dordoni C, Morlino S, et al. Spectrum of Mucocutaneous Manifestations in 277 Patients with Joint Hypermobility Syndrome/Ehlers-Danlos Syndrome, Hypermobility Type. *American Journal of Medical Genetics Part C-Seminars in Medical Genetics* 2015;169(1):43-53. doi: 10.1002/ajmg.c.31425

For peer review only - http://bmjopen.bmj.com/site/about/guidelines.xhtml

Table 1: Clinical coding for Ehlers-Danlos Syndromes and Joint Hypermobility Syndrome.

Read code descriptions (based on pre-1997 nomenclature)	EDS type according to the Villefranche Criteria	EDS Type according to the March 2017 Criteria	Read code version 2	ICD10 code
Ehlers-Danlos syndrome			PGy2.	Q79.6
Ehlers-Danlos syndrome type I	Classical type	Classical EDS	PGy20	
Ehlers-Danlos syndrome type II			PGy21	
Ehlers-Danlos syndrome type III	Hypermobility type	Hypermobile EDS or Hypermobility Spectrum Disorder	PGy22	
Ehlers-Danlos syndrome type IV	Vascular type	Vascular EDS	PGy23	
Ehlers-Danlos syndrome type V	X-linked type	No longer classified as EDS	PGy24	
Ehlers-Danlos syndrome type VI	Kyphoscoliotic type	Kyphoscoliotic EDS	PGy25	
Ehlers-Danlos syndrome type VII	Arthrochalasia type Dermatosparaxis type	Arthrochalasia EDS Dermatosparaxis EDS	PGy26	
Ehlers-Danlos syndrome type VIII	Periodontitis type	Periodontal EDS	PGy27	
Hypermobility Syndrome (JHS according to the Brighton Criteria)	(Hypermobility type)	Hypermobile EDS or Hypermobility Spectrum Disorder	N235.	728.5

For peer review only

For peer review only - http://bmjopen.bmj.com/site/about/guidelines.xhtml

Textbox 1

Ehlers Danlos Syndrome Nomenclature

- Joint hypermobility *per se* is reasonably common and thought to be present in around 10% of the general UK population [39].
- The Brighton criteria were used to diagnose Joint Hypermobility Syndrome from 1998 [40],
- The Villefranche criteria were applied to confirm EDS-Hypermobility Type from 1997 [41].
- Prior to the Villefranche criteria, the diagnosis EDS III was used to denote the hypermobile subtype of EDS.
- It was recognised over a number of years that JHS and EDS-HT were not distinct from one another [42].
- In March 2017 the International Consortium on the Ehlers Danlos Syndromes published a revised classification [43] naming two syndromes:
    - Hypermobile EDS (hEDS) which has narrowly defined criteria
    - Hypermobility Spectrum Disorder (HSD) for those with some but not all of the features of hEDS
- Patients who have a diagnosis of EDS-HT or JHS will fall into one of these two new categories.
- Castori et al showed that patients may move from the HSD category into hEDS over time: they also emphasised that the approach to management and the prognosis in terms of disability are the same [44]. One may therefore conclude that health needs across these groups are similar.

Figures

Figure 1: Flow diagram of EDS/JHS cohort and case-control cohort creation.Figure 2: Age at first coded diagnosis of EDS/JHS by age group and gender.

Figure 3: Prevalence of coded diagnosis of JHS/EDS in Primary Care, hospital inpatient and combined over time.

Figure 4: Odds ratios of Read chapter diagnoses for (a) young people (<18 years of age) and (b) adults (>= 18 years of age) within 12 months before and after EDS/JHS diagnosis. Presented are all results that affect at least 5 cases or 20 controls (perinatal conditions,

For peer review only - http://bmjopen.bmj.com/site/about/guidelines.xhtml

chapter Q, are not shown as neither young people nor adults had the required minimum number of cases/controls).

Figure 5: Odds ratios of Read chapter prescriptions for young people (<18 years of age) and adults (>= 18 years of age) within 12 months before and after EDS/JHS diagnosis. Presented are all results that affect at least 5 cases or 20 controls (incontinence and stoma appliances, chapters q and s, are not shown as neither young people nor adults had the required minimum number of cases/results).

Supplement Figures

Supplement Figure 1: Incidence of diagnosis of JHS/EDS in GP, hospital inpatient and combined data over time.

For peer review only

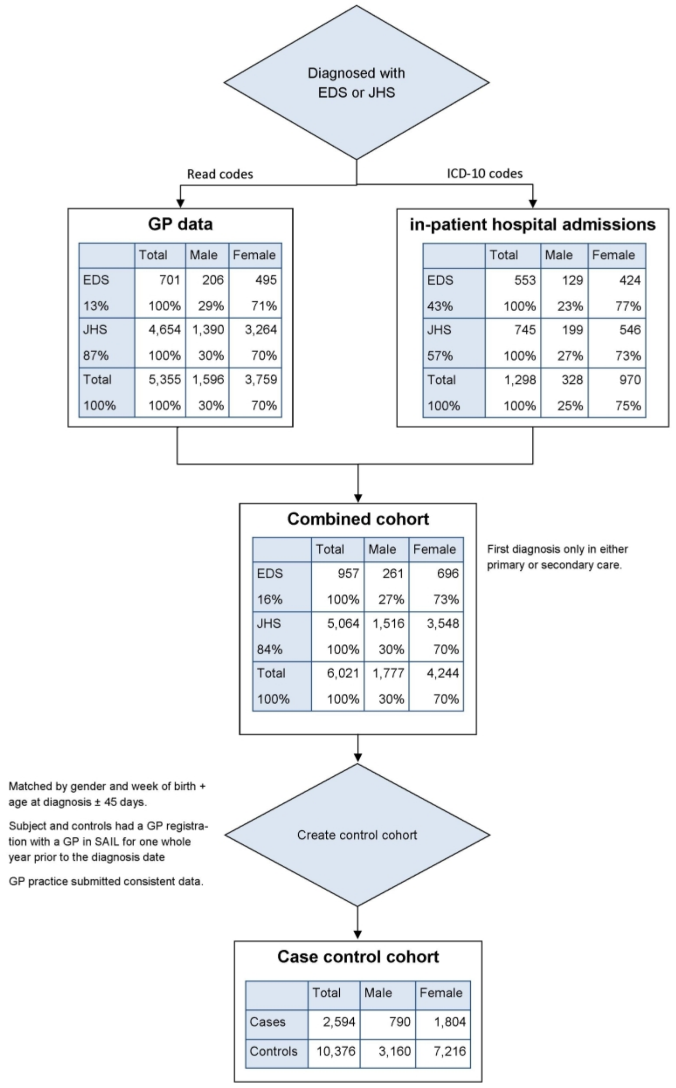

Flow diagram of EDS/JHS cohort and case-control cohort creation

209x297mm (300 x 300 DPI)

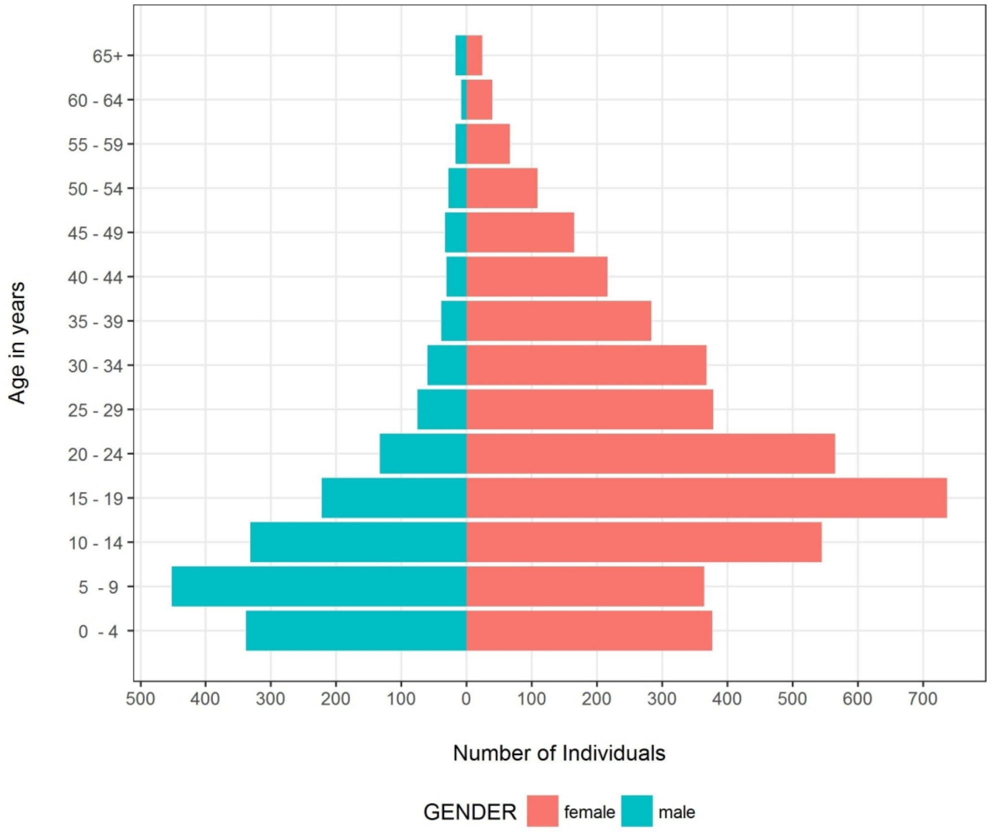

Age at first coded diagnosis of EDS/JHS by age group and gender

177x152mm (300 x 300 DPI)

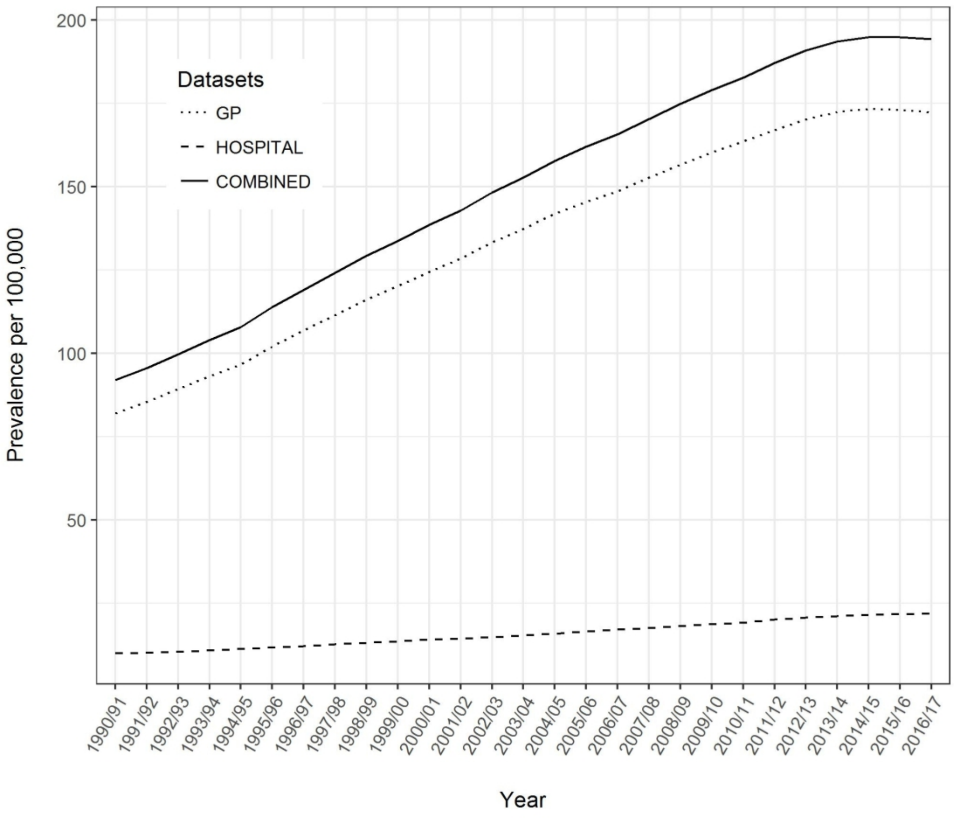

Prevalence of coded diagnosis of JHS/EDS in Primary Care, hospital inpatient and combined over time

177x152mm (300 x 300 DPI)

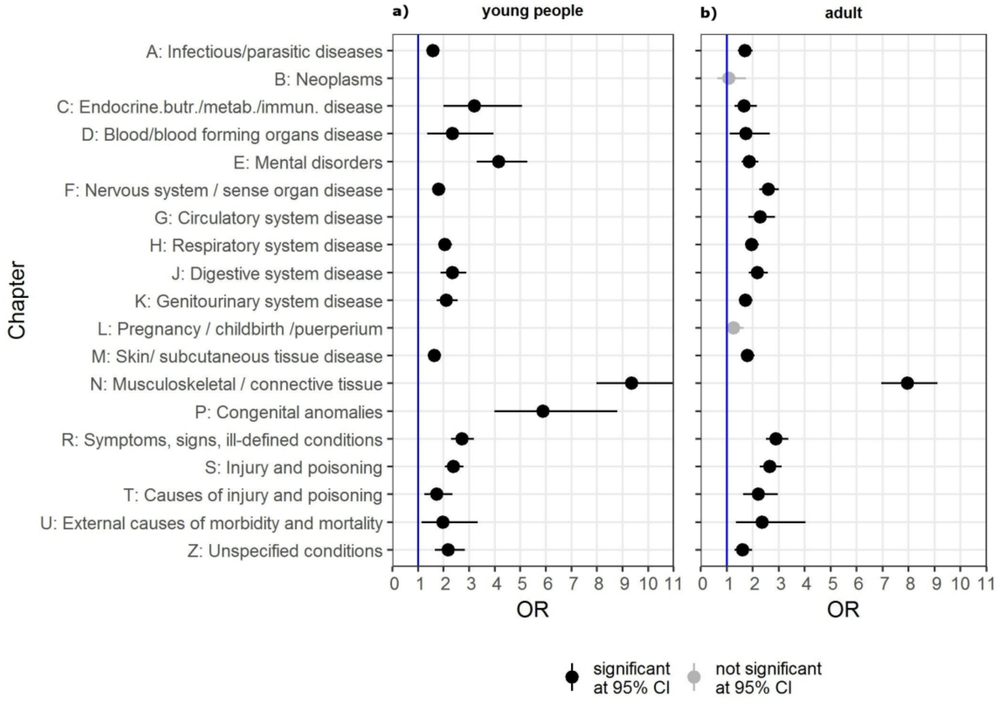

Odds ratios of Read chapter diagnoses for (a) young people (<18 years of age) and (b) adults (>= 18 years of age) within 12 months before and after EDS/JHS diagnosis. Presented are all results that affect at least 5 cases or 20 controls (perinatal conditions, chapter Q, are not shown as neither young people nor adults had the required minimum number of cases/controls)

177x127mm (300 x 300 DPI)

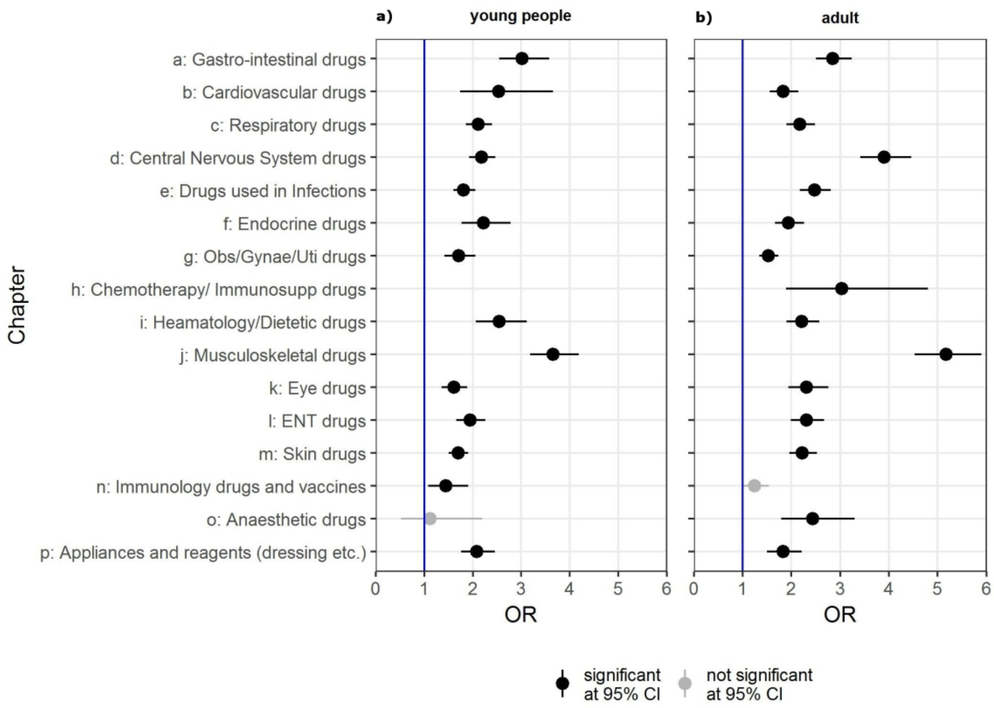

Odds ratios of Read chapter prescriptions for young people (<18 years of age) and adults (>= 18 years of age) within 12 months before and after EDS/JHS diagnosis. Presented are all results that affect at least 5 cases or 20 controls (incontinence and stoma appliances, chapters q and s, are not shown as neither young people nor adults had the required minimum number of cases/results)

177x127mm (300 x 300 DPI)

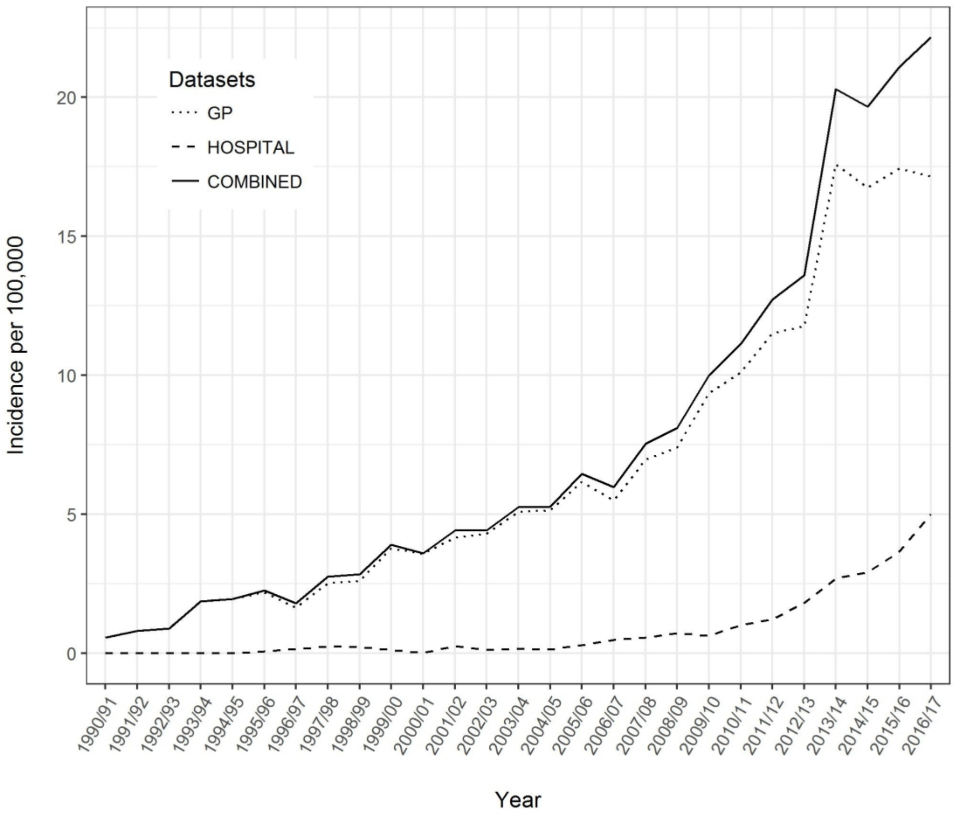

177x152mm (300 x 300 DPI)

The RECORD statement – checklist of items, extended from the STROBE statement, that should be reported in observational studies using routinely collected health data.

	Item No.	STROBE items	Location in manuscript where items are reported	RECORD items	Location in manuscript where items are reported
**Title and abstract**					
	1	(a) Indicate the study's design with a commonly used term in the title or the abstract (b) Provide in the abstract an informative and balanced summary of what was done and what was found	Title and abstract	RECORD 1.1: The type of data used should be specified in the title or abstract. When possible, the name of the databases used should be included.<br><br>RECORD 1.2: If applicable, the geographic region and timeframe within which the study took place should be reported in the title or abstract.<br><br>RECORD 1.3: If linkage between databases was conducted for the study, this should be clearly stated in the title or abstract.	Abstract p. 2<br><br>Abstract p. 2<br><br>Abstract p. 2
**Introduction**					
Background rationale	2	Explain the scientific background and rationale for the investigation being reported			Introduction p. 5-6
Objectives	3	State specific objectives, including any prespecified hypotheses			Introduction p. 6
**Methods**					
Study Design	4	Present key elements of study design early in the paper			Methods p. 7-9
Setting	5	Describe the setting, locations, and relevant dates, including periods of recruitment, exposure, follow-up, and data collection			Methods p. 7-9
Participants	6	*(a) Cohort study* - Give the eligibility criteria, and the		RECORD 6.1: The methods of study population selection (such as codes or	Methods p. 7-9 Table 1

For peer review only

		sources and methods of selection of participants. Describe methods of follow-up<br>*Case-control study* - Give the eligibility criteria, and the sources and methods of case ascertainment and control selection. Give the rationale for the choice of cases and controls<br>*Cross-sectional study* - Give the eligibility criteria, and the sources and methods of selection of participants<br><br>*(b) Cohort study* - For matched studies, give matching criteria and number of exposed and unexposed<br>*Case-control study* - For matched studies, give matching criteria and the number of controls per case		algorithms used to identify subjects) should be listed in detail. If this is not possible, an explanation should be provided.<br><br>RECORD 6.2: Any validation studies of the codes or algorithms used to select the population should be referenced. If validation was conducted for this study and not published elsewhere, detailed methods and results should be provided.<br><br>RECORD 6.3: If the study involved linkage of databases, consider use of a flow diagram or other graphical display to demonstrate the data linkage process, including the number of individuals with linked data at each stage.	 <br><br><br>NA, used standard clinical codes<br><br><br><br><br>Figure 1
Variables	7	Clearly define all outcomes, exposures, predictors, potential confounders, and effect modifiers. Give diagnostic criteria, if applicable.		RECORD 7.1: A complete list of codes and algorithms used to classify exposures, outcomes, confounders, and effect modifiers should be provided. If these cannot be reported, an explanation should be provided.	Methods p. 7-9 [calculated odds ratios]
Data sources/ measurement	8	For each variable of interest, give sources of data and details of methods of assessment (measurement).<br>Describe comparability of assessment methods if there is more than one group			Methods p. 7-9 Read codes in GP data<br>ICD-10 codes in PEDW data
Bias	9	Describe any efforts to address potential sources of bias			Methods p. 7-9 Case-control comparison only

For peer review only

					for years and GP practices with good data coverage
Study size	10	Explain how the study size was arrived at			Methods p. 7-9 Combined first diagnoses in GP and PEDW
Quantitative variables	11	Explain how quantitative variables were handled in the analyses. If applicable, describe which groupings were chosen, and why			Methods p. 7-9
Statistical methods	12	(a) Describe all statistical methods, including those used to control for confounding<br>(b) Describe any methods used to examine subgroups and interactions<br>(c) Explain how missing data were addressed<br>(d) *Cohort study* - If applicable, explain how loss to follow-up was addressed<br>*Case-control study* - If applicable, explain how matching of cases and controls was addressed<br>*Cross-sectional study* - If applicable, describe analytical methods taking account of sampling strategy<br>(e) Describe any sensitivity analyses			Methods p. 7-9<br>a) Simple odds ratios<br>b) Simple counts<br>c) NA (based on diagnoses)<br>d) Cohort – NA Case-control: week of birth and gender, dependant on registration with GP<br>e) NA
Data access and cleaning methods		..		RECORD 12.1: Authors should describe the extent to which the investigators had access to the database population used to create the study	Methods p 7-9

For peer review only

				population.<br><br>RECORD 12.2: Authors should provide information on the data cleaning methods used in the study.	 <br>Methods p. 7-9
Linkage		..		RECORD 12.3: State whether the study included person-level, institutional-level, or other data linkage across two or more databases. The methods of linkage and methods of linkage quality evaluation should be provided.	Methods p. 7-9
**Results**					
Participants	13	(a) Report the numbers of individuals at each stage of the study (*e.g.*, numbers potentially eligible, examined for eligibility, confirmed eligible, included in the study, completing follow-up, and analysed)<br>(b) Give reasons for non-participation at each stage.<br>(c) Consider use of a flow diagram		RECORD 13.1: Describe in detail the selection of the persons included in the study (*i.e.,* study population selection) including filtering based on data quality, data availability and linkage. The selection of included persons can be described in the text and/or by means of the study flow diagram.	Results p. 9-12<br>Figure 1
Descriptive data	14	(a) Give characteristics of study participants (*e.g.*, demographic, clinical, social) and information on exposures and potential confounders<br>(b) Indicate the number of participants with missing data for each variable of interest<br>(c) *Cohort study* - summarise follow-up time (*e.g.*, average and total amount)			a) p. 9-12<br>b) only exact matches, cannot identify missing data<br>c) NA
Outcome data	15	*Cohort study* - Report numbers of outcome events or summary measures over time<br>*Case-control study* - Report			p. 9-12<br>cohort: total number of people diagnosed in

For peer review only

		numbers in each exposure category, or summary measures of exposure<br>*Cross-sectional study* - Report numbers of outcome events or summary measures			either GP or PEDW<br><br>case-control: odds ratios
Main results	16	(a) Give unadjusted estimates and, if applicable, confounder-adjusted estimates and their precision (e.g., 95% confidence interval). Make clear which confounders were adjusted for and why they were included<br>(b) Report category boundaries when continuous variables were categorized<br>(c) If relevant, consider translating estimates of relative risk into absolute risk for a meaningful time period			p. 9-12<br>a) Simple odds ratios<br>b) Based on Read chapters<br>c) NA
Other analyses	17	Report other analyses done—e.g., analyses of subgroups and interactions, and sensitivity analyses			Young people vs. adults<br>Results p. 11
**Discussion**					
Key results	18	Summarise key results with reference to study objectives			p. 12
Limitations	19	Discuss limitations of the study, taking into account sources of potential bias or imprecision. Discuss both direction and magnitude of any potential bias		RECORD 19.1: Discuss the implications of using data that were not created or collected to answer the specific research question(s). Include discussion of misclassification bias, unmeasured confounding, missing data, and changing eligibility over time, as they pertain to the study being reported.	p. 12-13
Interpretation	20	Give a cautious overall interpretation of results considering objectives,			p. 12-13

For peer review only

		limitations, multiplicity of analyses, results from similar studies, and other relevant evidence			
Generalisability	21	Discuss the generalisability (external validity) of the study results			Discussion p. 12
**Other Information**					
Funding	22	Give the source of funding and the role of the funders for the present study and, if applicable, for the original study on which the present article is based			p. 14
Accessibility of protocol, raw data, and programming code		..		RECORD 22.1: Authors should provide information on how to access any supplemental information such as the study protocol, raw data, or programming code.	p. 16 data sharing statement

*Reference: Benchimol EI, Smeeth L, Guttmann A, Harron K, Moher D, Petersen I, Sørensen HT, von Elm E, Langan SM, the RECORD Working Committee. The REporting of studies Conducted using Observational Routinely-collected health Data (RECORD) Statement. *PLoS Medicine* 2015; in press.

*Checklist is protected under Creative Commons Attribution (CC BY) license.

For peer review only

BMJ Open

Diagnosed prevalence of Ehlers-Danlos syndrome and Hypermobility Spectrum Disorder in Wales, UK: a national electronic cohort study and case-control comparison

Journal:	*BMJ Open*
Manuscript ID	bmjopen-2019-031365.R2
Article Type:	Original research
Date Submitted by the Author:	23-Sep-2019
Complete List of Authors:	Demmler, Joanne; Swansea University, Swansea University Medical School<br>Atkinson, Mark; Swansea University, Swansea University Medical School<br>Reinhold, Emma; Royal College of General Practitioners<br>Choy, Ernest; Cardiff University, School of Medicine<br>Lyons, Ronan; Swansea University, Swansea University Medical School<br>Brophy, Sinead; Swansea University, Swansea University Medical School
**Primary Subject Heading**:	Public health
Secondary Subject Heading:	General practice / Family practice, Health informatics
Keywords:	Heritable Disorders of Connective Tissue, Ehlers-Danlos Syndromes, Joint Hypermobility Syndrome, Hypermobility Spectrum Disorder, Health Data Linkage, Prevalence

SCHOLARONE™
Manuscripts

Diagnosed prevalence of Ehlers-Danlos syndrome and Hypermobility Spectrum Disorder in Wales, UK: a national electronic cohort study and case-control comparison

Joanne C Demmler, Mark D Atkinson, Emma J Reinhold, Ernest Choy, Ronan A Lyons, Sinead T Brophy

Joanne C Demmler, Lecturer, Health Data Research UK, Swansea University, Singleton Park, Swansea SA2 8PP, UK. j.demmler@swansea.ac.uk.

Mark D Atkinson, Research Officer, Secure Anonymised Information Linkage (SAIL) Databank, Swansea University, Singleton Park, Swansea SA2 8PP, UK. m.atkinson@swansea.ac.uk.

Emma J Reinhold, RCGP Clinical Champion for the Ehlers-Danlos Syndromes, Clinical Innovation and Research Centre, Royal College of General Practitioners, 30 Euston Square, London, NW1 2FB. e.reinhold@doctors.org.uk.

Ernest Choy, Professor, Arthritis Research UK CREATE Centre, Section of Rheumatology, Division of Infection and Immunity, Cardiff University, Cardiff CF14 4YS, UK. ChoyEH@cardiff.ac.uk.

Ronan A Lyons, Professor, Health Data Research UK, Swansea University, Singleton Park, Swansea SA2 8PP, UK. r.a.lyons@swansea.ac.uk.

Sinead T Brophy, Professor, Health Data Research UK, Swansea University, Singleton Park, Swansea SA2 8PP, UK. s.brophy@swansea.ac.uk.

Correspondence to: JC Demmler j.demmler@swansea.ac.uk. Tel: 44 (0) 1792 295674

For peer review only

Abstract

Objectives: To describe the epidemiology of diagnosed Hypermobility Spectrum Disorder (HSD) and Ehlers-Danlos Syndromes (EDS) using linked electronic medical records. To examine whether these conditions remain rare and primarily affect the musculoskeletal system.

Design: Nationwide linked electronic cohort and nested case-control study

Setting: Routinely collected data from primary care and hospital admissions in Wales, UK.

Participants: People within the primary care or hospital data systems with a coded diagnosis of Ehlers-Danlos syndrome (EDS) or Joint Hypermobility Syndrome (JHS) between 01/07/1990 and 30/06/2017.

Main outcome measures: Combined prevalence of JHS and EDS in Wales. Additional diagnosis and prescription data in those diagnosed with EDS or JHS compared with matched controls.

Results: We found 6,021 individuals (male: 30%, female: 70%) with a diagnostic code of either EDS or JHS. This gives a diagnosed point prevalence of 194.2 per 100,000 in 2016/17 or roughly 10 cases in a practice of 5000 patients. There was a pronounced gender difference of 8.5 years (95% CI: 7.70 to 9.22) in the mean age at diagnosis. EDS or JHS was not only associated with high odds for other musculoskeletal diagnoses and drug prescriptions, but also with significantly higher odds of a diagnosis in other disease categories (e.g. mental health, nervous and digestive systems) and higher odds of a prescription in most disease categories (e.g. gastro-intestinal and cardiovascular drugs) within the 12 months before and after the first recorded diagnosis.

Conclusions: EDS and JHS (since March 2017 classified as EDS or HSD) have historically been considered rare diseases only affecting the musculoskeletal system and soft tissues. These data demonstrate that both of these assertions should be reconsidered.

For peer review only

Key Words: Heritable Disorders of Connective Tissue, Ehlers-Danlos Syndromes, Joint Hypermobility Syndrome, Hypermobility Spectrum Disorder, Health Data Linkage, Prevalence

Word Count: 3973

Strengths and Limitations

- Large cohort and nested case-control studies based on whole population routinely collected health data from primary and secondary care
- We are unable to quantify how many people are suffering from hypermobile EDS (hEDS) or HSD but remain undiagnosed, nor can we make any statement on the reliability of the diagnoses
- Although we only compared codes at Read chapter level all diagnoses and prescriptions can be matched to conditions found in the EDS/JHS literature

For peer review only

Introduction

The Ehlers-Danlos Syndromes (EDS) are a group of hereditary connective tissue disorders which mainly affect collagen. The nomenclature of these conditions has undergone a number of iterations which makes discussion of their prevalence complicated (see Textbox 1).

For many decades, studies have quoted a prevalence rate of 1 in 5000 for EDS, although the origin of this figure is unclear, seeming to appear first in a medical textbook [1 2] as an unreferenced "reasonable estimate". Thus, these syndromes have long been categorised as rare diseases, defined in the European Union as those affecting fewer than 50 in 100,000 people [3]. Kulas Søborg et al. [4] recently reported a prevalence of 20 per 100,000 for EDS in a nationwide Danish cohort based on secondary health care data up to 2012, but importantly, this data did not include patients who had received the considerably more common JHS diagnosis, now included in the latest revised classification. It is possible to extrapolate a combined population prevalence figure for JHS and EDS for Sweden [5] of around 120 per 100,000 from a study focussing on comorbid mental health issues, but no investigators have thus far set out to investigate the combined diagnosed prevalence of JHS/EDS within a population.

Although common features of these conditions are arthralgia, soft tissue injury and joint instability [6], over the last two decades it has become clear that their clinical features are not limited to musculoskeletal and cutaneous involvement, but are multisystemic [7-9]. In the special edition of the American Journal of Medical Genetics dedicated to EDS in March 2017, papers covered links to cardiovascular autonomic [10] and gastrointestinal dysfunction [11] as well as psychiatric and neurodevelopmental disorders [5 12]. Chronic disabling fatigue [13] and pain syndromes [14] were also recognised as common and multifactorial issues. Gynaecological [15 16] and obstetric [17] issues are also reported in this population. There is also an emerging link with the potentially life-threatening condition of Mast Cell Activation

For peer review only - http://bmjopen.bmj.com/site/about/guidelines.xhtml

Syndrome [18 19]. There is some emerging evidence hinting that nutritional deficiencies [20 21] may play a key role, both seeming to be more prevalent in these patients and possibly implicated in the development of some of the complications.

Early diagnosis is found to be crucial to patients [22] to enable the provision of appropriate treatment, as well as to prevent later onset complications [7]. Establishing the diagnosis of EDS/HSD is often problematic for patients, which interferes with the early detection, treatment and prevention of further escalations of recognised symptoms, disability and more elaborate complications. A mean of 14 years elapses between the first clinical manifestations and the actual diagnosis [23]. For 25% of patients this delay lasts over 28 years [23]. "A misdiagnosis was given to 56% of patients [resulting in] inappropriate treatment in 70% of the patients… For 86% of the patients, the delay in diagnosis was considered responsible for deleterious consequences." [23(p.137)]

It is possible that some of these difficulties arise from the widespread belief amongst clinicians that EDS is rare. It is therefore of clinical importance to establish better estimates of current prevalence. Conventional studies tend to be based in restricted clinical settings, such as rheumatology clinics, and are therefore limited by the number of recruited patients and biased by severity/type of patients referred. It has been shown that using linked health data is an economic and effective alternative to performing *de novo* longitudinal studies, including rare conditions [24 25]. We used routinely held data from primary and secondary care sources to examine the epidemiology of people with a diagnostic code for EDS/JHS in Wales. We then conducted a nested case- control study to study the number of diagnoses across all body/disease systems and prescription usage in order to test the widespread belief that these conditions are primarily musculoskeletal in nature, rather than multi-system disorders.

Methods

For peer review only - http://bmjopen.bmj.com/site/about/guidelines.xhtml

Study design: Nationwide electronic cohort study

Anonymised record linkage and hosting is carried out in the Secure Anonymised Information Linkage (SAIL) databank [26] on routinely collected data held in health and social care datasets. All data within the SAIL gateway are treated in accordance with the Data Protection Act 2018 and complies with GDPR.

We used data from a variety of datasets between 01/07/1990 and 30/06/2017 to create the anonymised e-cohort and case control studies. The Primary Care data covers about 80% of all coded information held by General Practitioners (GPs) in Wales. The Welsh Demographics Service (WDS) contains key statistics, such as gender, week of birth, date of death and practice migration status for everyone in Wales registered with a GP. The Patient Episode Database for Wales (PEDW) contains all inpatient hospital admissions to a Welsh hospital. WDS and PEDW data are available for the whole of Wales. The SAIL databank enables the anonymised matching of individuals across these different datasets using a person level anonymised linkage field (ALF) [26].

Cohort preparation

We identified Welsh residents with a Read Version 2 [27] diagnostic code of EDS or JHS in primary care data or ICD-10 diagnostic codes [28] in secondary care data (hospital admissions) between 01/07/1990 (or the start of the dataset if later) and 30/06/2017. This date marks the end of maximum data coverage across all datasets. The EDS sub-classification in Read Version 2 contains some, but not all, of the subtypes which were in use prior to 1997 and as a result, the reliability of any subtype data must be highly questionable (see Table 1). Due to the lack of available correct sub-codes for EDS subtypes, the fact that the overwhelming majority of patients simply had the header code (86% of those coded as EDS, with a further 12% coded as hEDS), and that other EDS types are genuinely rare, all codes for EDS were combined. ICD-10 codes do not distinguish between any subtypes of EDS (see Table 1). Only ALF's with good matching status were included in the

For peer review only - http://bmjopen.bmj.com/site/about/guidelines.xhtml

study, i.e. direct match on either NHS number or on surname, first name, postcode, date of birth and gender; or fuzzy matching with a probability of >= 90%.

We created one dataset for diagnoses in the GP data and another for diagnoses in the hospital data. Both datasets were linked to the week of birth, gender and date of death information in WDS on their ALF and then combined to create a cohort of people with EDS/JHS in either GP or hospital data, identifying any duplications and keeping the earliest diagnosis date for any individual appearing in both datasets.

Analysis

Data linkage and data preparation within the SAIL databank were conducted using IBM DB2 10.5 SQL. Data were then imported into R (Version 3.4.1) [29], which was used for all statistical analyses. The mean age at first diagnosis between male and female subjects was compared and confidence intervals of the difference calculated.

The denominator of the diagnosed prevalence and incidence of EDS and JHS in secondary care was calculated based on the total number of individuals with recorded gender, registered and living in Wales between 01/07/1990 and 30/06/2017 for each full year of the study respectively. The prevalence and incidence in primary care denominator was further adjusted to include only people living in Wales and whose GP practice was contributing data to SAIL. The prevalence and incidence in primary and secondary care was then added together to create an overall estimate of the prevalence and incidence in Wales.

Case-Control Comparison

A nested case control method was used. Each case was matched to 4 controls with the same gender and similar age profiles (within 45 days of the week of birth). We implemented strict criteria for selection to the case-control cohort. Both cases and controls had to (a) have uninterrupted GP registrations for 1 year before and 1 year after the date of the relevant diagnosis (or died during follow-up); (b) be registered with a GP submitting data to SAIL either at the matching date or afterwards; (c) have been registered with a GP that

For peer review only - http://bmjopen.bmj.com/site/about/guidelines.xhtml

consistently recorded data across their patient profile. The latter avoids diagnoses that were retrospectively entered for a time period when the GP practice did not fully implement the use of electronic records (less than 10% of the data they recorded during 2009). Although this reduced the number of cases and controls we were able to analyse, it avoids data quality bias, especially during the early years of this study, when GPs were converting to the use of computer systems and databases. Controls with any type of diagnosed hereditary connective tissue disorder were excluded. Preliminary analysis of the combined cohort indicated that adjustment for deprivation was not necessary (i.e. equal distribution of people across deprivation quintiles). We then calculated odds ratios between cases and controls using Read chapters (excluding the Read codes for EDS and JHS). This method counts the number of people with a code in each category; multiple codes for the same person in the same category are therefore not included. All results that affected at least 5 cases or 20 controls were visualised using forest plots.

Ethical approval

The study design uses anonymised data and therefore the need for ethical approval and participant consent was waived by the approving Institutional Review Board, the UK National Health Service Research Ethics Committee. The SAIL independent Information Governance Review Panel (IGRP) approved the study.

Patient and Public Involvement

Two of the authors of this paper have been diagnosed with symptomatic joint hypermobility disorders. This study used routinely collected data, we were not able to involve members of the public but will be disseminating our findings widely, including directly to patients via social media and through our links with patient organisations.

Results

EDS/JHS in Primary Care data

For peer review only - http://bmjopen.bmj.com/site/about/guidelines.xhtml

5,355 individuals with a diagnosis of either EDS or JHS with valid birth and gender information were identified. Of these, 4,654 (87%) had a diagnosis of JHS and 701 (13%) of EDS. The Read code for the EDS subtype was only used for 136 (19%) individuals with 114 (16%) identified as EDS-Hypermobility Type and 22 (3%) as other EDS subtypes. 3,759 (70%) of those diagnosed with EDS/JHS were female (see Figure 1).

EDS/JHS in Hospital Data

A total of 1,298 individuals were found in the hospital data of whom 970 (75%) were female: 745 (57%) had a diagnosis of JHS and 553 (43%) EDS (see Figure 1).

Demographics of combined EDS/JHS cohort

5,355 (89%) of the cases could be found in the primary care data with the remainder in the hospital cohort. Combining the results from primary and secondary care led to a cohort of 6,021 distinct individuals. 5,064 (84%) were coded with JHS and 957 (16%) with EDS. 4,244 (70%) of patients were female. The age at first diagnosis peaked in the age group 5-9 years for males and 15-19 years for females (see Figure 2). There was a significant difference of 8.5 years in the mean age of diagnosis between males and females (95% CI: 7.70 to 9.22): 9.6 years in EDS (95% CI: 6.85 to 12.31) and 8.3 years in JHS (95% CI: 7.58 to 9.11). 72% of males were diagnosed during childhood (age < 18 years) in contrast to only 41% of females.

2016/17 is the latest year for which we have complete data and could therefore derive prevalence. During this year, 2,668,902 people were registered with a GP in Wales submitting data to SAIL, of whom 4,598 had a diagnostic code of EDS/JHS which first appeared in the primary care data (172 in 100,000). A further 711 people out of the 3,239,153 registered with any GP in Wales during 2016/17 had an EDS/JHS diagnosis which first appears in secondary care data (22 in 100,000). There is an increasing rate of coded diagnoses throughout the period. Assuming that the GP data is representative of the whole of Wales this leads to a combined point prevalence of 194 in 100,000 at the end of the

For peer review only - http://bmjopen.bmj.com/site/about/guidelines.xhtml

study period. This corresponds to about 10 cases in a practice of 5000 patients (see Figure 3). The incidence of EDS/JHS over this time period is shown in Supplement Figure 1.

Factors associated with JHS/EDS

2,597 cases had good GP data coverage at the age of diagnosis and could be matched by age and gender with controls (see Figure 1). 1,340 cases (male: 561; female: 779) were first diagnosed before the age of 18 years and 1,254 cases (male: 229; female: 1,025) above this age. The people in the nested case-control cohort were slightly older than the overall cohort (data not shown here).

Looking at the time frame of 1 year either side of the first coded diagnosis of EDS/JHS amongst young people (age < 18 years) there were significantly more additional diagnoses in 16 out of 20 Read code disease categories compared with their controls (see Figure 4a). The top three Read diagnosis chapters with increased odds for the EDS/JHS cohort were for musculoskeletal conditions (OR 9.36, 95% CI: 7.98 to 11.00), congenital anomalies (OR 5.89; 95% CI: 3.98 to 8.80) and mental disorders (OR 4.16; 95% CI: 3.29 to 5.27).

People that were diagnosed as adults (age >= 18 years) had also significantly more diagnoses in 16 out of 20 Read code categories than their controls (see Figure 4b). The top three Read diagnosis chapters for adults with higher odds in the EDS/JHS cohort were musculoskeletal disorders (OR 7.95; 95% CI: 6.95 to 9.12), congenital anomalies (OR 5.18; 95% CI: 2.78 to 9.78) and symptoms, signs and ill-defined conditions (OR 2.9; 95% CI: 2.5 to 3.37). Circulatory system disease (OR 2.29; 95% CI: 1.83 to 2.86) and mental disorders remained significant (OR 1.87; 95% CI: 1.57 to 2.22), but not to the same extend as they were for young people.

Young people showed significantly higher odds for prescriptions in 14 out of 17 Read code categories then their controls (see Figure 5a). The top three prescriptions Read chapters with increased odds for the EDS/JHS cohort were for (i) musculoskeletal drugs (OR 3.65;

For peer review only - http://bmjopen.bmj.com/site/about/guidelines.xhtml

95% CI: 3.18 to 4.18), (ii) gastro-intestinal drugs (OR 3.02; 95% CI: 2.54 to 3.58) and (iii) haematology/dietetic drugs (OR 2.54; 95% CI: 2.06 to 3.11).

Adults had significantly higher odds of prescriptions for 15 out of 17 Read code categories (see Figure 5b). The top three prescriptions with higher odds for EDS/JHS people were for (i) musculoskeletal drugs (OR 5.17; 95% CI: 4.53 to 5.9), (ii) central nervous system drugs (OR 3.9; 95% CI: 3.41 to 4.46) and (iii) chemotherapy/immunosuppressant drugs (OR 3.03; 95% CI: 1.89 to 4.8). Gastro-intestinal drugs (OR 2.85; 95% CI: 2.5 to 3.24) and haematology/dietetic drugs (OR 2.21; 95% CI: 1.9 to 2.57) remain significant, but at slightly lower levels than in the young EDS/JHS population.

Discussion

This work examined the epidemiology of EDS and JHS and found a combined diagnosed prevalence of 194.2 per 100,000 (0.19%), or 1 in 500 people in Wales; hEDS or HSD within the 2017 classification. We found a steadily increasing rate of diagnosis over the past 27 years (see Supplement Figure 1), as well as higher rates of diagnoses for other conditions and prescriptions within 12 months (pre and post) of the recorded first diagnosis in most categories. This suggests that hEDS/HSD, when considered together, do not meet the definition of rare conditions [23], and have widespread effects across multiple body systems. It is well-known that EDS is poorly recognised in children [30 31] and initial symptoms and EDS-associated diagnoses can appear to be simply a ‘normal’ pattern of childhood illness when taken as an isolated event. Furthermore, children with hEDS often present with symptoms that can lead to a misdiagnosis of mental illness or consideration of child abuse [12 32]. Suspicion of abuse has been shown to be extremely damaging to the mental health of the parent(s) and can lead to an avoidance of accessing health care or other public services, such as schools [33]. The prolonged and sometimes traumatic diagnosis and/or misdiagnosis process in EDS can lead to further disengagement with services [34]. The lack of a timely diagnosis has great implications for disease management and progression and impedes the

For peer review only - http://bmjopen.bmj.com/site/about/guidelines.xhtml

appropriate consideration of surgical interventions [7 35-38] as well as pregnancy and birth planning [17]. It is perhaps only in stepping back to look at the pattern of effects across multiple body systems that practitioners might begin to consider a connective tissue disorder.

Strengths and Limitations

The strength of this study is that we were able to combine diagnostic codes from several primary and secondary health care providers to create a large cohort of individuals with EDS/JHS. We have 27 years of data with at least 11 years of very good data coverage in the key datasets, which further improves with each data update of the SAIL databank, however data coverage for the first couple of years is less comprehensive.

The majority of subjects were identified via their primary care data, which is a strength and a weakness. As 89% of cases were identified through primary care data studies not using primary care data may underestimate the prevalence of hEDS/HSD. We are unable to quantify how many people are suffering from hEDS or HSD but remain undiagnosed. However, we cannot comment on the reliability of the diagnoses in the primary care dataset. It is also likely that the majority of cases were not actually diagnosed in primary care, but their entries were created through secondary care contacts, such as outpatient appointments or musculoskeletal assessment clinics, but coded data are lacking from these sources.

Although a snapshot of Read chapters codes that are more prevalent in our JHS/EDS cohort does not allow us to look at specific diagnoses and prescriptions, they can all be matched to conditions associated with EDS/JHS in the literature, for instance pain, fatigue, cardiovascular, gastrointestinal and gynaecological disorders, dysautonomia, mast cell activation as well as urinary tract infections [7]. It needs to be stressed that these results exclude codes for EDS/JHS and that these are not part of the results for congenital anomalies or musculoskeletal conditions. We hope in future work to examine in greater detail these findings of significant differences between people with hEDS/HSD and others in

For peer review only - http://bmjopen.bmj.com/site/about/guidelines.xhtml

order that we can better understand the nature of this condition, as well as potentially improving diagnostic recognition. For instance, immunosuppressant drugs are in the same Read chapter as chemotherapy drugs. Higher use of these prescriptions in the hEDS/HSD cohort could plausibly be linked to the known increased comorbid existence of disorders such as inflammatory bowel disease, inflammatory arthropathies, systemic lupus erythematosis and other autoimmune conditions [39], and is less likely to be due to a higher rate of use of chemotherapeutic agents.

We conclude that EDS/HSD are not rare conditions and are associated with significantly increased odds of additional diagnoses and use of medications across many body systems. There is a large gender difference in the age of diagnosis, with many women not diagnosed until adulthood. Early diagnosis, however, is crucial to patients, the administration of preventive therapies, the investigation of comorbid conditions and the overall management process. Further research is needed to understand patient pathways, comorbidities and progression of associated symptoms and diseases. Health services should be aware of these findings for the provision of training, diagnostic and treatment services for the many tens of thousands of patients living with these life-changing conditions throughout the United Kingdom and beyond.

Author contributions

JD conceived the project. SB and MA contributed to the study design and analysis plan. RAL validated clinical codes in primary care. EC validated clinical codes in secondary care. JD undertook the analysis. JD and ER carried out the literature reviews and drafted the manuscript. All authors reviewed the manuscript and approved the final version for submission.

Funding

This work was supported by The Farr Institute. The Farr Institute was supported by a 10-funder consortium: Arthritis Research UK, the British Heart Foundation, Cancer Research UK, the

For peer review only - http://bmjopen.bmj.com/site/about/guidelines.xhtml

Economic and Social Research Council, the Engineering and Physical Sciences Research Council, the Medical Research Council, the National Institute of Health Research, the National Institute for Social Care and Health Research (Welsh Assembly Government), the Chief Scientist Office (Scottish Government Health Directorates), the Wellcome Trust, (MRC Grant No: MR/K006525/1).

This work was also supported by Health Data Research UK which receives its funding from HDR UK Ltd (NIWA1) funded by the UK Medical Research Council, Engineering and Physical Sciences Research Council, Economic and Social Research Council, Department of Health and Social Care (England), Chief Scientist Office of the Scottish Government Health and Social Care Directorates, Health and Social Care Research and Development Division (Welsh Government), Public Health Agency (Northern Ireland), British Heart Foundation (BHF) and the Wellcome Trust.

The above funders played no role in the study design, in the collection, analysis, or interpretation of data; in the writing of the report; or in the decision to submit the article for publication. The researchers are independent from the funders and all authors, external and internal, had full access to all of the data in the study and take responsibility for the integrity of the data and the accuracy of the data analysis.

Competing interests: All authors have completed the ICMJE uniform disclosure form at www.icmje.org/coi_disclosure.pdf and declare: no support from any organisation for the submitted work; no financial relationships with any organisations that might have an interest in the submitted work in the previous three years; no other relationships or activities that could appear to have influenced the submitted work.

Exclusive licence: The Corresponding Author has the right to grant on behalf of all authors and does grant on behalf of all authors, a worldwide licence (http://www.bmj.com/sites/default/files/BMJ%20Author%20Licence%20March%202013.doc)

For peer review only - http://bmjopen.bmj.com/site/about/guidelines.xhtml

to the Publishers and its licensees in perpetuity, in all forms, formats and media (whether known now or created in the future), to i) publish, reproduce, distribute, display and store the Contribution, ii) translate the Contribution into other languages, create adaptations, reprints, include within collections and create summaries, extracts and/or, abstracts of the Contribution and convert or allow conversion into any format including without limitation audio, iii) create any other derivative work(s) based in whole or part on the on the Contribution, iv) to exploit all subsidiary rights to exploit all subsidiary rights that currently exist or as may exist in the future in the Contribution, v) the inclusion of electronic links from the Contribution to third party material wherever it may be located; and, vi) licence any third party to do any or all of the above. All research articles will be made available on an open access basis (with authors being asked to pay an open access fee—see http://www.bmj.com/about-bmj/resources-authors/forms-policies-and-checklists/copyright-open-access-and-permission-reuse). The terms of such open access shall be governed by a Creative Commons licence—details as to which Creative Commons licence will apply to the research article are set out in our worldwide licence referred to above.

For peer review only

Transparency declaration: The lead author affirms that this manuscript is an honest, accurate, and transparent account of the study being reported; that no important aspects of the study have been omitted; and that any discrepancies from the study as planned (and, if relevant, registered) have been explained.

Dissemination statement: We are planning to disseminate our results to patient groups using social media.

Data sharing statement: The data used in this study are available in the SAIL databank at Swansea University, Swansea, UK. All proposals to use SAIL data are subject to review by an independent Information Governance Review Panel (IGRP). Before any data can be accessed, approval must be given by the IGRP.

For peer review only - http://bmjopen.bmj.com/site/about/guidelines.xhtml

The IGRP gives careful consideration to each project to ensure proper and appropriate use of SAIL data. When access has been granted, it is gained through a privacy-protecting safe haven and remote access system referred to as the SAIL Gateway. SAIL has established an application process to be followed by anyone who would like to access data via SAIL https://www.saildatabank.com/application-process.

Additional resources: RCGP Clinical Toolkit on the Ehlers-Danlos Syndromes www.rcgp.org.uk/eds; RCGP Podcast “Introduction to Ehlers-Danlos Syndromes” https://audioboom.com/posts/6896541-introduction-to-ehlers-danlos-syndromes

References

1. Cecil RLF, Goldman L, Bennett JC. Cecil Textbook of Medicine: W.B. Saunders 2000.
2. Mulvey MR, Macfarlane GJ, Beasley M, et al. Modest Association of Joint Hypermobility With Disabling and Limiting Musculoskeletal Pain: Results From a Large-Scale General Population-Based Survey. *Arthritis Care & Research* 2013;65(8):1325-33. doi: 10.1002/acr.21979
3. Commission of the European Communities Communication from the Commission to the European Parliament, the Council, the European Economic and Social Committee and the Committee of the regions on Rare Diseases: Europe's challenges. Brussels: Commission of the European Communities, 2008.
4. Søborg M-LK, Leganger J, Mortensen LQ, et al. Establishment and baseline characteristics of a nationwide Danish cohort of patients with Ehlers–Danlos syndrome. *Rheumatology (Oxford)* 2017;56(5):763-67. doi: 10.1093/rheumatology/kew478
5. Cederlöf M, Larsson H, Lichtenstein P, et al. Nationwide population-based cohort study of psychiatric disorders in individuals with Ehlers-Danlos syndrome or hypermobility syndrome and their siblings. *Bmc Psychiatry* 2016;16 doi: 10.1186/s12888-016-0922-6
6. Hakim AJ, Grahame R. A simple questionnaire to detect hypermobility: An adjunct to the assessment of patients with diffuse musculoskeletal pain. *International Journal of Clinical Practice* 2003;57(3):163-66.
7. Tinkle B, Castori M, Berglund B, et al. Hypermobile Ehlers-Danlos Syndrome (a.k.a. Ehlers-Danlos Syndrome Type III and Ehlers-Danlos Syndrome Hypermobility Type): Clinical Description and Natural History. *American Journal of Medical Genetics Part C-Seminars in Medical Genetics* 2017;175(1):48-69. doi: 10.1002/ajmg.c.31538
8. Gazit Y, Jacob G, Grahame R. Ehlers-Danlos Syndrome-Hypermobility Type: A Much Neglected Multisystemic Disorder. *Rambam Maimonides Medical Journal* 2016;7(4) doi: 10.5041/rmmj.10261
9. De Paepe A, Malfait F. The Ehlers-Danlos syndrome, a disorder with many faces. *Clinical Genetics* 2012;82(1):1-11. doi: 10.1111/j.1399-0004.2012.01858.x
10. Hakim A, O'Callaghan C, De Wandele I, et al. Cardiovascular Autonomic Dysfunction in Ehlers-Danlos Syndrome-Hypermobile Type. *American Journal of Medical Genetics*

For peer review only - http://bmjopen.bmj.com/site/about/guidelines.xhtml

Part C-Seminars in Medical Genetics 2017;175(1):168-74. doi: 10.1002/ajmg.c.31543

11. Fikree A, Chelimsky G, Collins H, et al. Gastrointestinal Involvement in the Ehlers-Danlos Syndromes. *American Journal of Medical Genetics Part C-Seminars in Medical Genetics* 2017;175(1):181-87. doi: 10.1002/ajmg.c.31546
12. Bulbena A, Baeza-Velasco C, Bulbena-Cabre A, et al. Psychiatric and Psychological Aspects in the Ehlers-Danlos Syndromes. *American Journal of Medical Genetics Part C-Seminars in Medical Genetics* 2017;175(1):237-45. doi: 10.1002/ajmg.c.31544
13. Hakim A, De Wandele I, O'Callaghan C, et al. Chronic Fatigue in Ehlers-Danlos Syndrome-Hypermobile Type. *American Journal of Medical Genetics Part C-Seminars in Medical Genetics* 2017;175(1):175-80. doi: 10.1002/ajmg.c.31542
14. Chopra P, Tinkle B, Hamonet C, et al. Pain Management in the Ehlers-Danlos Syndromes. *American Journal of Medical Genetics Part C-Seminars in Medical Genetics* 2017;175(1):212-19. doi: 10.1002/ajmg.c.31554
15. Veit-Rubin N, Cartwright R, Singh AU, et al. Association between joint hypermobility and pelvic organ prolapse in women: a systematic review and meta-analysis. *International Urogynecology Journal* 2016;27(10):1469-78. doi: 10.1007/s00192-015-2896-1
16. Hugon-Rodin J, Lebegue G, Becourt S, et al. Gynecologic symptoms and the influence on reproductive life in 386 women with hypermobility type ehlers-danlos syndrome: a cohort study. *Orphanet Journal of Rare Diseases* 2016;11 doi: 10.1186/s13023-016-0511-2
17. Pezaro S, Pearce G, Reinhold E. Hypermobile Ehlers-Danlos Syndrome during pregnancy, birth and beyond. *British Journal of Midwifery* 2018;26(4):217-23. doi: 10.12968/bjom.2018.26.4.217
18. Seneviratne SL, Maitland A, Afrin L. Mast Cell Disorders in Ehlers-Danlos Syndrome. *American Journal of Medical Genetics Part C-Seminars in Medical Genetics* 2017;175(1):226-36. doi: 10.1002/ajmg.c.31555
19. Cheung I, Vadas P. A New Disease Cluster: Mast Cell Activation Syndrome, Postural Orthostatic Tachycardia Syndrome, and Ehlers-Danlos Syndrome. *Journal of Allergy and Clinical Immunology* 2015;135(2):AB65-AB65.
20. Mantle D, Wilkins RM, Preedy V. A novel therapeutic strategy for Ehlers-Dantos syndrome based on nutritional supplements. *Medical Hypotheses* 2005;64(2):279-83. doi: 10.1016/j.mehy.2004.07.023
21. Castori M, Morlino S, Pascolini G, et al. Gastrointestinal and Nutritional Issues in Joint Hypermobility Syndrome/Ehlers-Danlos Syndrome, Hypermobility Type. *American Journal of Medical Genetics Part C-Seminars in Medical Genetics* 2015;169(1):54-75. doi: 10.1002/ajmg.c.31431
22. Terry RH, Palmer ST, Rimes KA, et al. Living with joint hypermobility syndrome: patient experiences of diagnosis, referral and self-care. *Family Practice* 2015;32(3):354-58. doi: 10.1093/fampra/cmv026
23. EURORDIS AKFF. The Voice of 12,000 Patients. Experiences and Expectations of Rare Disease Patients on Diagnosis and Care in Europe: Eurordis 2009.
24. Holman CDJ, Bass AJ, Rosman DL, et al. A decade of data linkage in Western Australia: strategic design, applications and benefits of the WA data linkage system. *Australian Health Review* 2008;32(4):766-77.
25. Cooksey R, Husain MJ, Brophy S, et al. The Cost of Ankylosing Spondylitis in the UK Using Linked Routine and Patient-Reported Survey Data. *Plos One* 2015;10(7) doi: 10.1371/journal.pone.0126105
26. Lyons RA, Jones KH, John G, et al. The SAIL databank: linking multiple health and social care datasets. *Bmc Medical Informatics and Decision Making* 2009;9 doi: 10.1186/1472-6947-9-3
27. Chisholm J. The READ Clinical Classification. *Brit Med J* 1990;300(6732):1092-92.
28. World Health Organization. ICD-10: International statistical classification of diseases and related health problems: World Health Organization 2016.

For peer review only - http://bmjopen.bmj.com/site/about/guidelines.xhtml

29. R: A Language and Environment for Statistical Computing [program]. Vienna, Austria: R Foundation for Statistical Computing, 2017.
30. Adib N, Davies K, Grahame R, et al. Joint hypermobility syndrome in childhood. A not so benign multisystem disorder? *Rheumatology* 2005;44(6):744-50. doi: 10.1093/rheumatology/keh557
31. Grahame R, Hakim AJ. Joint Hypermobility Syndrome is highly prevalent in general rheumatology clinics, its occurrence and clinical presentation being gender, age and race-related. *Annals of the Rheumatic Diseases* 2006;65:263-63.
32. Castori M. Ehlers-Danlos Syndrome(s) Mimicking Child Abuse: Is There an Impact on Clinical Practice? (vol 169, pg 289, 2015). *American Journal of Medical Genetics Part A* 2016;170(7):1947-47. doi: 10.1002/ajmg.a.37733
33. Association for Improvements in Maternity Services (AIMS). The Child Protection System in England: Written evidence submitted by the Association for Improvements in the Maternity Services (AIMS) 2011 [Available from: http://www.publications.parliament.uk/pa/cm201012/cmselect/cmeduc/writev/1514/cp29.htm accessed 14/02/2019.
34. Berglund B, Anne-Cathrine M, Randers I. Dignity not fully upheld when seeking health care: Experiences expressed by individuals suffering from Ehlers–Danlos syndrome. *Disability and Rehabilitation* 2010;32(1):1-7. doi: 10.3109/09638280903178407
35. Scheper MC, Nicholson LL, Adams RD, et al. The natural history of children with joint hypermobility syndrome and Ehlers-Danlos hypermobility type: a longitudinal cohort study. *Rheumatology* 2017;56(12):2073-83. doi: 10.1093/rheumatology/kex148
36. Engelbert RHH, Juul-Kristensen B, Pacey V, et al. The Evidence-Based Rationale for Physical Therapy Treatment of Children, Adolescents, and Adults Diagnosed With Joint Hypermobility Syndrome/Hypermobile Ehlers Danlos Syndrome. *American Journal of Medical Genetics Part C-Seminars in Medical Genetics* 2017;175(1):158-67. doi: 10.1002/ajmg.c.31545
37. Ericson WB, Wolman R. Orthopaedic Management of the Ehlers-Danlos Syndromes. *American Journal of Medical Genetics Part C-Seminars in Medical Genetics* 2017;175(1):188-94. doi: 10.1002/ajmg.c.31551
38. Soborg MLK, Leganger J, Rosenberg J, et al. Increased Need for Gastrointestinal Surgery and Increased Risk of Surgery-Related Complications in Patients with Ehlers-Danlos Syndrome: A Systematic Review. *Digestive Surgery* 2017;34(2):161-70. doi: 10.1159/000449106
39. Rodgers KR, Gui J, Dinulos MBP, et al. Ehlers-Danlos syndrome hypermobility type is associated with rheumatic diseases. *Scientific Reports* 2017;7 doi: 10.1038/srep39636
40. Sobey G. Ehlers-Danlos syndrome: how to diagnose and when to perform genetic tests. *Archives of Disease in Childhood* 2015;100(1):57-61. doi: 10.1136/archdischild-2013-304822
41. Grahame R, Bird HA, Child A, et al. The revised (Brighton 1998) criteria for the diagnosis of benign joint hypermobility syndrome (BJHS). *Journal of Rheumatology* 2000;27(7):1777-79.
42. Beighton P, De Paepe A, Steinmann B, et al. Ehlers-Danlos syndromes: Revised nosology, Villefranche, 1997. *American Journal of Medical Genetics* 1998;77(1):31-37. doi: 10.1002/(sici)1096-8628(19980428)77:1<31::aid-ajmg8>3.0.co;2-o
43. Grahame R. Joint hypermobility and genetic collagen disorders: are they related? *Archives of Disease in Childhood* 1999;80(2):188-91.
44. Malfait F, Francomano C, Byers P, et al. The 2017 International Classification of the Ehlers-Danlos Syndromes. *American Journal of Medical Genetics Part C-Seminars in Medical Genetics* 2017;175(1):8-26. doi: 10.1002/ajmg.c.31552
45. Castori M, Dordoni C, Morlino S, et al. Spectrum of Mucocutaneous Manifestations in 277 Patients with Joint Hypermobility Syndrome/Ehlers-Danlos Syndrome, Hypermobility Type. *American Journal of Medical Genetics Part C-Seminars in Medical Genetics* 2015;169(1):43-53. doi: 10.1002/ajmg.c.31425

For peer review only - http://bmjopen.bmj.com/site/about/guidelines.xhtml

For peer review only

For peer review only - http://bmjopen.bmj.com/site/about/guidelines.xhtml

Table 1: Clinical coding for Ehlers-Danlos Syndromes and Joint Hypermobility Syndrome.

Read code descriptions (based on pre-1997 nomenclature)	EDS type according to the Villefranche Criteria	EDS Type according to the March 2017 Criteria	Read code version 2	ICD10 code
Ehlers-Danlos syndrome			PGy2.	Q79.6
Ehlers-Danlos syndrome type I	Classical type	Classical EDS	PGy20	
Ehlers-Danlos syndrome type II			PGy21	
Ehlers-Danlos syndrome type III	Hypermobility type	Hypermobile EDS or Hypermobility Spectrum Disorder	PGy22	
Ehlers-Danlos syndrome type IV	Vascular type	Vascular EDS	PGy23	
Ehlers-Danlos syndrome type V	X-linked type	No longer classified as EDS	PGy24	
Ehlers-Danlos syndrome type VI	Kyphoscoliotic type	Kyphoscoliotic EDS	PGy25	
Ehlers-Danlos syndrome type VII	Arthrochalasia type Dermatosparaxis type	Arthrochalasia EDS Dermatosparaxis EDS	PGy26	
Ehlers-Danlos syndrome type VIII	Periodontitis type	Periodontal EDS	PGy27	
Hypermobility Syndrome (JHS according to the Brighton Criteria)	(Hypermobility type)	Hypermobile EDS or Hypermobility Spectrum Disorder	N235.	728.5

For peer review only

Textbox 1: An overview of the Ehlers-Danlos Nomenclature

Ehlers Danlos Syndrome Nomenclature

- Joint hypermobility *per se* is reasonably common and thought to be present in around 10% of the general UK population [40].
- The Brighton criteria were used to diagnose Joint Hypermobility Syndrome from 1998 [41],
- The Villefranche criteria were applied to confirm EDS-Hypermobility Type from 1997 [42].
- Prior to the Villefranche criteria, the diagnosis EDS III was used to denote the hypermobile subtype of EDS.
- It was recognised over a number of years that JHS and EDS-HT were not distinct from one another [43].
- In March 2017 the International Consortium on the Ehlers Danlos Syndromes published a revised classification [44] naming two syndromes:
    - Hypermobile EDS (hEDS) which has narrowly defined criteria
    - Hypermobility Spectrum Disorder (HSD) for those with some but not all of the features of hEDS
- Patients who have a diagnosis of EDS-HT or JHS will fall into one of these two new categories.
- Castori et al showed that patients may move from the HSD category into hEDS over time: they also emphasised that the approach to management and the prognosis in terms of disability are the same [45]. One may therefore conclude that health needs across these groups are similar.

Figures

Figure 1: Flow diagram of EDS/JHS cohort and case-control cohort creation.Figure 2: Age at first coded diagnosis of EDS/JHS by age group and gender.

Figure 3: Prevalence of coded diagnosis of JHS/EDS in Primary Care, hospital inpatient and combined over time.

Figure 4: Odds ratios of Read chapter diagnoses for (a) young people (<18 years of age) and (b) adults (>= 18 years of age) within 12 months before and after EDS/JHS diagnosis. Presented are all results that affect at least 5 cases or 20 controls (perinatal conditions,

For peer review only - http://bmjopen.bmj.com/site/about/guidelines.xhtml

chapter Q, are not shown as neither young people nor adults had the required minimum number of cases/controls).

Figure 5: Odds ratios of Read chapter prescriptions for young people (<18 years of age) and adults (>= 18 years of age) within 12 months before and after EDS/JHS diagnosis. Presented are all results that affect at least 5 cases or 20 controls (incontinence and stoma appliances, chapters q and s, are not shown as neither young people nor adults had the required minimum number of cases/results).

Supplement Figures

Supplement Figure 1: Incidence of diagnosis of JHS/EDS in GP, hospital inpatient and combined data over time.

For peer review only

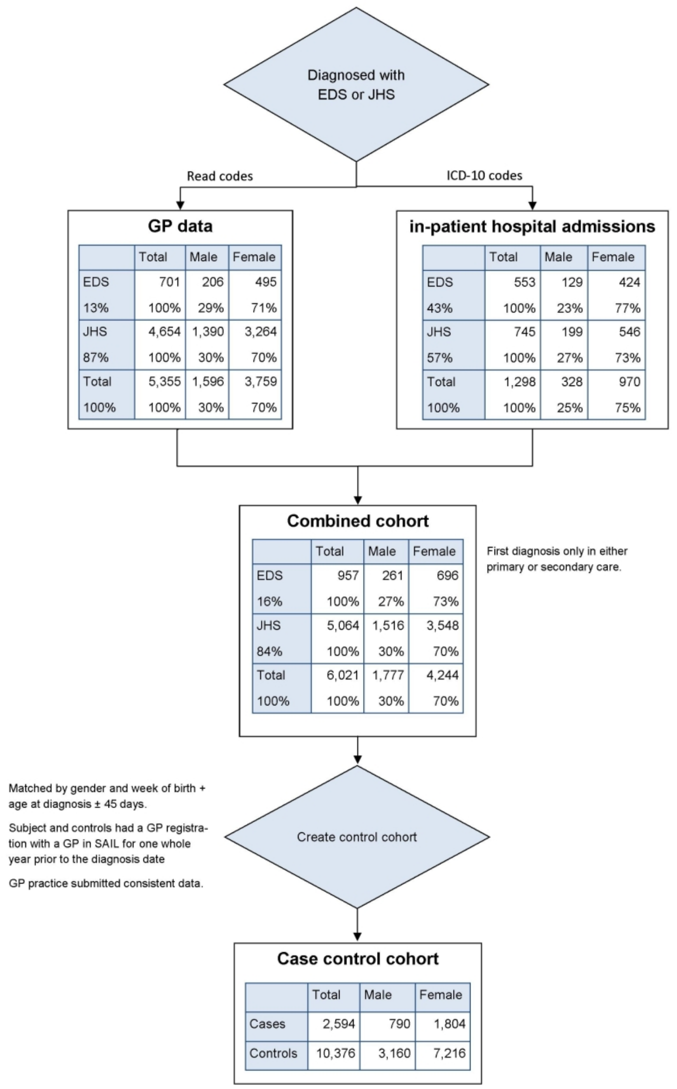

Flow diagram of EDS/JHS cohort and case-control cohort creation

209x297mm (300 x 300 DPI)

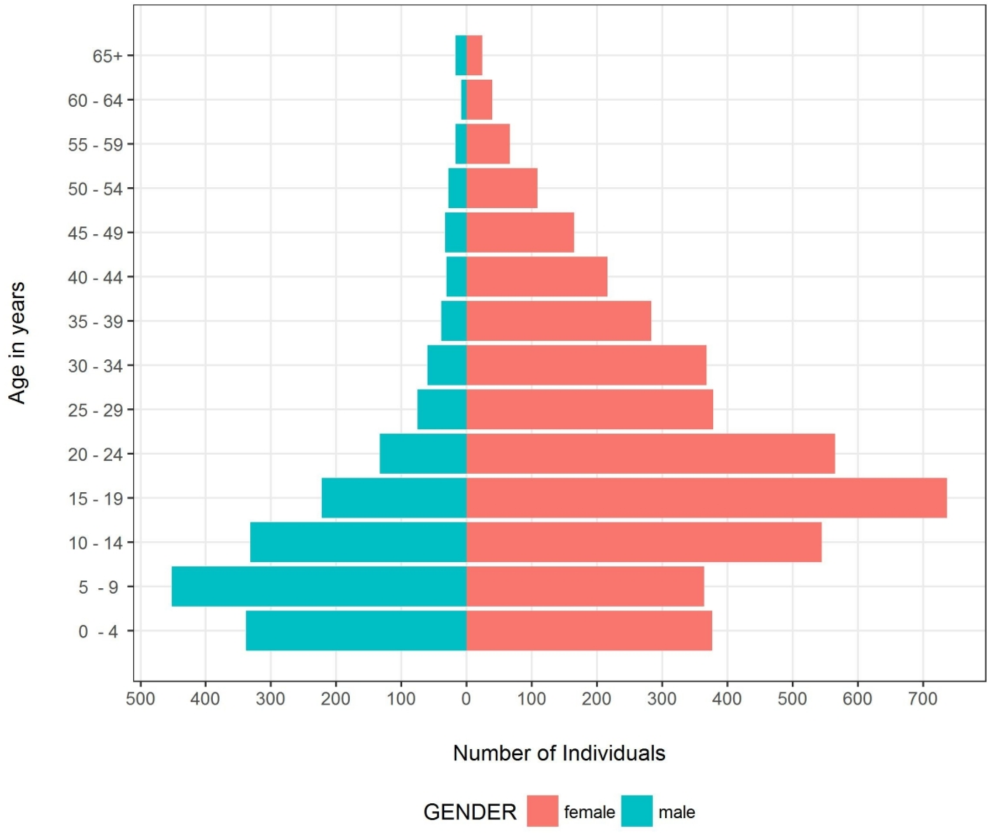

Age at first coded diagnosis of EDS/JHS by age group and gender

177x152mm (300 x 300 DPI)

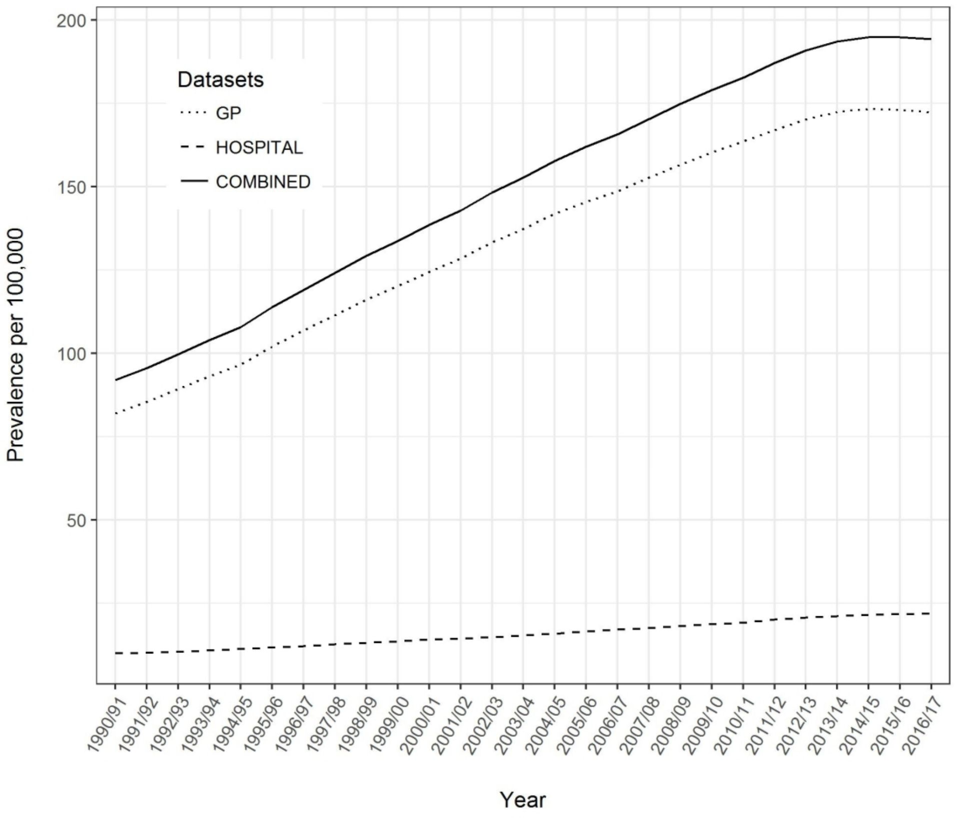

Prevalence of coded diagnosis of JHS/EDS in Primary Care, hospital inpatient and combined over time

177x152mm (300 x 300 DPI)

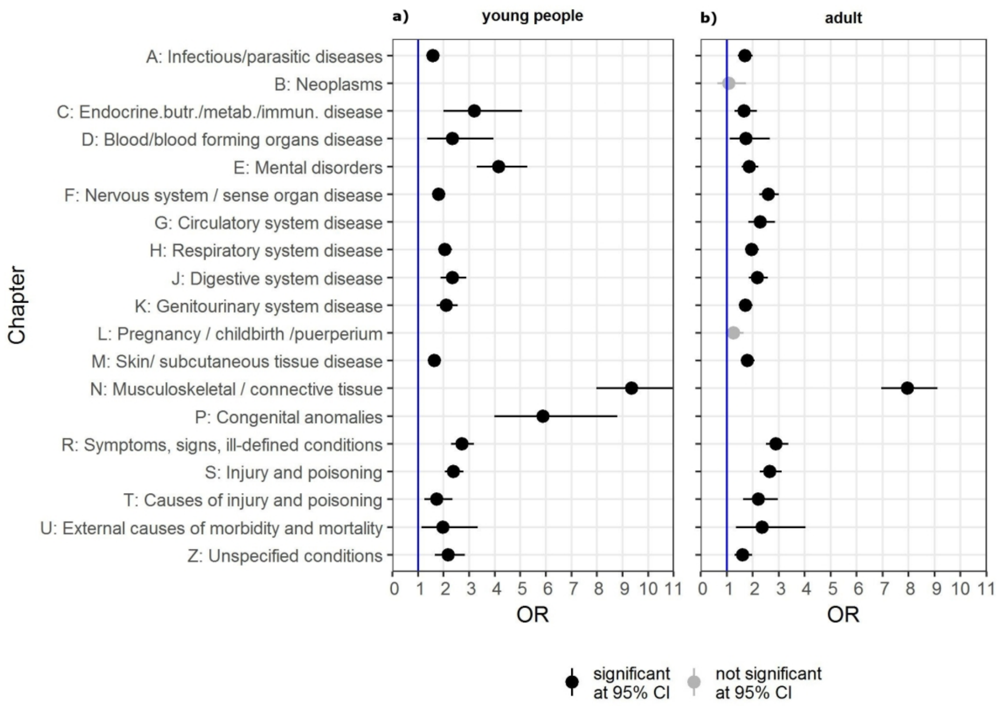

Odds ratios of Read chapter diagnoses for (a) young people (<18 years of age) and (b) adults (>= 18 years of age) within 12 months before and after EDS/JHS diagnosis. Presented are all results that affect at least 5 cases or 20 controls (perinatal conditions, chapter Q, are not shown as neither young people nor adults had the required minimum number of cases/controls)

177x127mm (300 x 300 DPI)

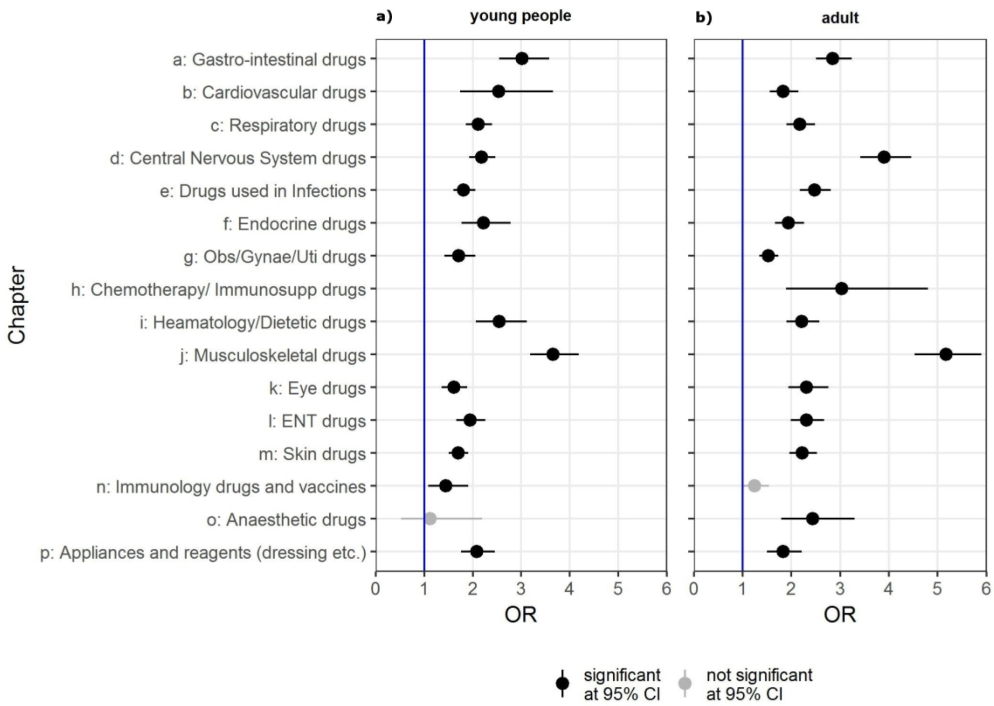

Odds ratios of Read chapter prescriptions for young people (<18 years of age) and adults (>= 18 years of age) within 12 months before and after EDS/JHS diagnosis. Presented are all results that affect at least 5 cases or 20 controls (incontinence and stoma appliances, chapters q and s, are not shown as neither young people nor adults had the required minimum number of cases/results)

177x127mm (300 x 300 DPI)

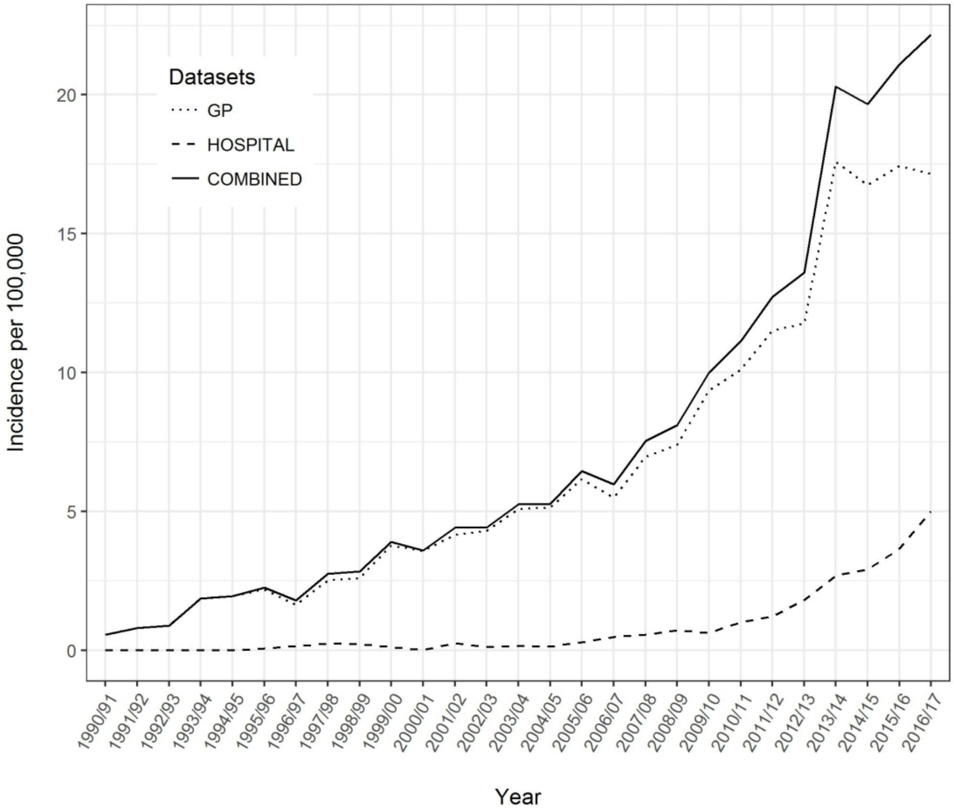

177x152mm (300 x 300 DPI)

The RECORD statement – checklist of items, extended from the STROBE statement, that should be reported in observational studies using routinely collected health data.

	Item No.	STROBE items	Location in manuscript where items are reported	RECORD items	Location in manuscript where items are reported
**Title and abstract**					
	1	(a) Indicate the study's design with a commonly used term in the title or the abstract (b) Provide in the abstract an informative and balanced summary of what was done and what was found	Title and abstract	RECORD 1.1: The type of data used should be specified in the title or abstract. When possible, the name of the databases used should be included.<br><br>RECORD 1.2: If applicable, the geographic region and timeframe within which the study took place should be reported in the title or abstract.<br><br>RECORD 1.3: If linkage between databases was conducted for the study, this should be clearly stated in the title or abstract.	Abstract p. 2<br><br>Abstract p. 2<br><br>Abstract p. 2
**Introduction**					
Background rationale	2	Explain the scientific background and rationale for the investigation being reported			Introduction p. 5-6
Objectives	3	State specific objectives, including any prespecified hypotheses			Introduction p. 6
**Methods**					
Study Design	4	Present key elements of study design early in the paper			Methods p. 7-9
Setting	5	Describe the setting, locations, and relevant dates, including periods of recruitment, exposure, follow-up, and data collection			Methods p. 7-9
Participants	6	*(a) Cohort study* - Give the eligibility criteria, and the		RECORD 6.1: The methods of study population selection (such as codes or	Methods p. 7-9 Table 1

For peer review only

		sources and methods of selection of participants. Describe methods of follow-up<br>*Case-control study* - Give the eligibility criteria, and the sources and methods of case ascertainment and control selection. Give the rationale for the choice of cases and controls<br>*Cross-sectional study* - Give the eligibility criteria, and the sources and methods of selection of participants<br><br>*(b) Cohort study* - For matched studies, give matching criteria and number of exposed and unexposed<br>*Case-control study* - For matched studies, give matching criteria and the number of controls per case		algorithms used to identify subjects) should be listed in detail. If this is not possible, an explanation should be provided.<br><br>RECORD 6.2: Any validation studies of the codes or algorithms used to select the population should be referenced. If validation was conducted for this study and not published elsewhere, detailed methods and results should be provided.<br><br>RECORD 6.3: If the study involved linkage of databases, consider use of a flow diagram or other graphical display to demonstrate the data linkage process, including the number of individuals with linked data at each stage.	 <br><br><br>NA, used standard clinical codes<br><br><br><br><br><br>Figure 1
Variables	7	Clearly define all outcomes, exposures, predictors, potential confounders, and effect modifiers. Give diagnostic criteria, if applicable.		RECORD 7.1: A complete list of codes and algorithms used to classify exposures, outcomes, confounders, and effect modifiers should be provided. If these cannot be reported, an explanation should be provided.	Methods p. 7-9 [calculated odds ratios]
Data sources/ measurement	8	For each variable of interest, give sources of data and details of methods of assessment (measurement).<br>Describe comparability of assessment methods if there is more than one group			Methods p. 7-9<br>Read codes in GP data<br>ICD-10 codes in PEDW data
Bias	9	Describe any efforts to address potential sources of bias			Methods p. 7-9<br>Case-control comparison only

For peer review only

					for years and GP practices with good data coverage
Study size	10	Explain how the study size was arrived at			Methods p. 7-9 Combined first diagnoses in GP and PEDW
Quantitative variables	11	Explain how quantitative variables were handled in the analyses. If applicable, describe which groupings were chosen, and why			Methods p. 7-9
Statistical methods	12	(a) Describe all statistical methods, including those used to control for confounding<br>(b) Describe any methods used to examine subgroups and interactions<br>(c) Explain how missing data were addressed<br>(d) *Cohort study* - If applicable, explain how loss to follow-up was addressed<br>*Case-control study* - If applicable, explain how matching of cases and controls was addressed<br>*Cross-sectional study* - If applicable, describe analytical methods taking account of sampling strategy<br>(e) Describe any sensitivity analyses			Methods p. 7-9<br>a) Simple odds ratios<br>b) Simple counts<br>c) NA (based on diagnoses)<br>d) Cohort – NA Case-control: week of birth and gender, dependant on registration with GP<br>e) NA
Data access and cleaning methods		..		RECORD 12.1: Authors should describe the extent to which the investigators had access to the database population used to create the study	Methods p 7-9

For peer review only

				population.<br><br>RECORD 12.2: Authors should provide information on the data cleaning methods used in the study.	 <br>Methods p. 7-9
Linkage		..		RECORD 12.3: State whether the study included person-level, institutional-level, or other data linkage across two or more databases. The methods of linkage and methods of linkage quality evaluation should be provided.	Methods p. 7-9
**Results**					
Participants	13	(a) Report the numbers of individuals at each stage of the study (*e.g.*, numbers potentially eligible, examined for eligibility, confirmed eligible, included in the study, completing follow-up, and analysed)<br>(b) Give reasons for non-participation at each stage.<br>(c) Consider use of a flow diagram		RECORD 13.1: Describe in detail the selection of the persons included in the study (*i.e.,* study population selection) including filtering based on data quality, data availability and linkage. The selection of included persons can be described in the text and/or by means of the study flow diagram.	Results p. 9-12<br>Figure 1
Descriptive data	14	(a) Give characteristics of study participants (*e.g.*, demographic, clinical, social) and information on exposures and potential confounders<br>(b) Indicate the number of participants with missing data for each variable of interest<br>(c) *Cohort study* - summarise follow-up time (*e.g.*, average and total amount)			a) p. 9-12<br>b) only exact matches, cannot identify missing data<br>c) NA
Outcome data	15	*Cohort study* - Report numbers of outcome events or summary measures over time<br>*Case-control study* - Report			p. 9-12<br>cohort: total number of people diagnosed in

For peer review only

		numbers in each exposure category, or summary measures of exposure<br>*Cross-sectional study* - Report numbers of outcome events or summary measures			either GP or PEDW<br><br>case-control: odds ratios
Main results	16	(a) Give unadjusted estimates and, if applicable, confounder-adjusted estimates and their precision (e.g., 95% confidence interval). Make clear which confounders were adjusted for and why they were included<br>(b) Report category boundaries when continuous variables were categorized<br>(c) If relevant, consider translating estimates of relative risk into absolute risk for a meaningful time period			p. 9-12<br>a) Simple odds ratios<br>b) Based on Read chapters<br>c) NA
Other analyses	17	Report other analyses done—e.g., analyses of subgroups and interactions, and sensitivity analyses			Young people vs. adults<br>Results p. 11
**Discussion**					
Key results	18	Summarise key results with reference to study objectives			p. 12
Limitations	19	Discuss limitations of the study, taking into account sources of potential bias or imprecision. Discuss both direction and magnitude of any potential bias		RECORD 19.1: Discuss the implications of using data that were not created or collected to answer the specific research question(s). Include discussion of misclassification bias, unmeasured confounding, missing data, and changing eligibility over time, as they pertain to the study being reported.	p. 12-13
Interpretation	20	Give a cautious overall interpretation of results considering objectives,			p. 12-13

For peer review only

		limitations, multiplicity of analyses, results from similar studies, and other relevant evidence			
Generalisability	21	Discuss the generalisability (external validity) of the study results			Discussion p. 12
**Other Information**					
Funding	22	Give the source of funding and the role of the funders for the present study and, if applicable, for the original study on which the present article is based			p. 14
Accessibility of protocol, raw data, and programming code		..		RECORD 22.1: Authors should provide information on how to access any supplemental information such as the study protocol, raw data, or programming code.	p. 16 data sharing statement

*Reference: Benchimol EI, Smeeth L, Guttmann A, Harron K, Moher D, Petersen I, Sørensen HT, von Elm E, Langan SM, the RECORD Working Committee. The REporting of studies Conducted using Observational Routinely-collected health Data (RECORD) Statement. *PLoS Medicine* 2015; in press.

*Checklist is protected under Creative Commons Attribution (CC BY) license.

For peer review only
